# Analytical expressions for the time evolution of spin systems affected by two or more interactions

**DOI:** 10.5194/mr-6-77-2025

**Published:** 2025-02-27

**Authors:** Günter Hempel

**Affiliations:** 1 Martin-Luther-Universität Halle-Wittenberg, Institut für Physik – NMR, Betty-Heimann-Str. 7, 06120 Halle, Germany

## Abstract

Analytical expressions for the description of the time evolution of spin systems beyond product–operator formalism (POF) can be obtained if a low-dimensional subspace of the Liouville space has been found in which the time evolution of the spin system takes place completely. This can be achieved using a procedure that consists of repeated application of the commutator of the Hamiltonian with the density operator. This iteration continues as long as the result of such a commutator operation contains a term that is linearly independent of all the operators appearing in the previous commutator operations. The coefficients of the resulting system of commutator relations can be immediately inserted into the generic propagation formulae given in this article if the system contains two, three, or four equations. In cases where the validity conditions of any of these propagation formulae are not met, the coefficients are used as intermediate steps to obtain both the Liouvillian and propagator matrices of the system. Several application examples are given where an analytical equation can be obtained for the description of the time evolution of small spin systems under the influence of two or more interactions. This procedure for finding the Liouvillian matrix is not limited to time-independent interactions. Some examples illustrate the treatment of time-dependent problems using this method.

## 1 Introduction

Considerable progress has been made in predicting the time evolution of spin systems. Numerical calculations or simulations are possible in large systems of coupled spins [Bibr bib1.bibx12], and the evolution during an arbitrarily long sequence of pulses can be simulated with the help of software [Bibr bib1.bibx24], including the effect of thermal motion.

However, there may be situations where the analytical representation of the time evolution is advantageous, providing more physical intuition than a numerical procedure, which can seem like a black box. The desire or need to take a look inside this black box could be a motivation to deal with analytical contexts. In many cases, product–operator formalism (POF) [Bibr bib1.bibx20] has been used for this purpose. This is a rather intuitive scheme where the states of the spin system are represented by spin operators. Such a tool can be very useful for doing short calculations without access to fundamental quantum mechanics or without a simulation program. For demonstrations in discussions and lectures, e.g., on the effects of pulse sequences like INEPT or HSQC [Bibr bib1.bibx22], an illustrative explanation can be given here.

As an example, consider the POF description of the propagation of transversal magnetization of spin I = 1/2 which is coupled by a scalar interaction to another spin S = 1/2 (coupling constant J ):

1 $${\hat{I}}_{x}\overset{2\pi J{\hat{I}}_{z}{\hat{S}}_{z}\cdot t}{⟶}{\hat{I}}_{x}\mathrm{cos}\pi Jt+2{\hat{I}}_{y}{\hat{S}}_{z}\mathrm{sin}\pi Jt.$$

This means that the spin system oscillates between two states characterized by the operators ${\hat{I}}_{z}$ and $2{\hat{I}}_{z}{\hat{S}}_{z}$ . Thus, the evolution takes place in a 2-D subspace of the total operator space. In contrast, to describe a system with two spins 1/2 , we need a 4×4 density matrix. This means that the Liouville–von Neumann equation is a system of 16 scalar differential equations for each of the matrix elements. However, obviously it is possible in this example to reduce the 16-D problem to the 2-D one described by Eq. ([Disp-formula Ch1.E1]).

A question arises: are there other situations where dimensionality reduction is possible? Candidates for such situations are, for example, the evolution of a spin system under dipoledipole interaction and simultaneous rf irradiation, or cross-polarization with respect to the finite RF power.

$$\begin{array}{c} {\hat{I}}_{z}\overset{\text{dipole–dipole interaction}\;+\;\text{rf irradiation}}{⟶}?\\ {\hat{I}}_{x}\overset{\text{cross-polarization under a finite rf power}}{⟶}? \end{array}$$

Are there others? For numerical calculations, any reduction in dimensionality leads to a reduction in computation time, but to enable analytical calculations, which is the goal here, this may be essential. The aims of this paper are (i) to introduce a procedure for reducing the dimension of the problem, (ii) to show examples of the application of this procedure to obtain analytical equations, and (iii) to show the resulting generic propagation rules as templates for cases where the problem could be reduced to a 2-D, 3-D, or 4-D problem. The latter can be seen as an extension of the product–operator formalism to somewhat more complex situations. (i) and (ii) can be useful for simplifying some calculations through dimension reduction without any approximation. Even for numerical calculations, this can be useful if it helps us to work on low-dimensional systems. As an example, a system of two spins I and S is mentioned here, which is subject to dipolar interaction but which is decoupled by rf irradiation, and at the same time magic-angle sample spinning (MAS) modulating the dipolar oscillation takes place. Its time evolution can be described by a system of three differential equations using the method presented here. The application of the Shirley–Floquet method will be greatly simplified here. On the other hand, the application of the Liouville–von Neumann equation in the 4-D wave-function space leads to a system of 16 differential equations, even if some of its coefficients can be 0.

In Sect. 2, the mathematical background is investigated, which makes it possible to reduce the dimension to the value 2 in cases where the POF is applicable. The results of this calculation are applied to more complex structures in Sect. 3. Finally, in Sect. 4, some template formulae are given, together with examples of cases where a reduction to 3-D, 4-D, 5-D, and 6-D problems is possible.

As usual, in this paper operators are denoted by a hat ( $\hat{A}$ ) and superoperators by a double hat ( $\hat{\hat{B}}$ ), while the vector or matrix associated with an operator is denoted by the same symbol but in italics and in bold roman, respectively, and without a hat ( A and B ). Scalar variables are written in italics.

## 2 Dimension reduction through POF

### 2.1 Condition for validity: commutator relations

As shown in the references cited above, the generic scheme of the POF is as follows. The time evolution can be predicted by the propagation rules

2 $$\begin{array}{l} \hat{A}\overset{\hat{H}\;t}{⟶}\hat{A}\mathrm{cos}\lambda t+\hat{B}\mathrm{sin}\lambda t\;\text{and}\\ \hat{B}\overset{\hat{H}\;t}{⟶}\hat{B}\mathrm{cos}\lambda t-\hat{A}\mathrm{sin}\lambda t \end{array}$$

if and only if

3 $$\begin{array}{l} \left[\hat{H},\hat{A}\right]=\;\;i\lambda \hat{B}\;\;\text{and}\\ \left[\hat{H},\hat{B}\right]=-i\lambda \hat{A}, \end{array}$$

where $\left[\hat{A},\hat{B}\right]\equiv \hat{A}\hat{B}-\hat{B}\hat{A}$ denotes the commutator between the operators $\hat{A}$ and $\hat{B}$ . That is, Eq. ([Disp-formula Ch1.E2]) describes the motion of the density operator in a 2-D subspace of the total Liouville space of the current spin system, although the Liouville space has a much larger dimension.

However, Eq. ([Disp-formula Ch1.E3]) is often not satisfied when more than one interaction must be considered. This applies, for example, to an rf irradiation with strength $\omega_{1}$ in an ensemble of spins I = 1/2 which are coupled to another spin ensemble S = 1/2 (coupling frequency $D_{\mathrm{IS}}$ ). This situation can be described by the Hamiltonian $\hat{H}=-2D_{\mathrm{IS}}{\hat{I}}_{z}{\hat{S}}_{z}-\omega_{1}{\hat{I}}_{x}$ and an initial state $\rho_{0}={\hat{I}}_{z}$ . The double calculation of the commutator of the Hamiltonian with ${\hat{I}}_{z}$ results not only in ${\hat{I}}_{z}$ , but also an additional term. In this case, after the third application of this commutator operation, the result will contain only the operators already used:

4 $$\begin{array}{l} \left[\hat{H},{\hat{I}}_{z}\right]=\;\;i\omega_{1}\;{\hat{I}}_{y},\\ \left[\hat{H},{\hat{I}}_{y}\right]=-iD_{\mathrm{IS}}\cdot 2{\hat{I}}_{x}{\hat{S}}_{z}-i\omega_{1}\;{\hat{I}}_{z},\\ \left[\hat{H},2{\hat{I}}_{x}{\hat{S}}_{z}\right]=\;\;iD_{\mathrm{IS}}\cdot {\hat{I}}_{y}. \end{array}$$

The connection between the commutator Eq. ([Disp-formula Ch1.E3]) and the propagation Eq. ([Disp-formula Ch1.E2]) becomes much clearer if we reformulate both sets of equations in matrix form,

5 $$\left(\begin{array}{c} \hat{A}\\ \hat{B} \end{array}\right)\overset{\hat{H}\cdot t}{⟶}\left(\begin{array}{cc} \mathrm{cos}\lambda t & \mathrm{sin}\lambda t\\ -\mathrm{sin}\lambda t & \mathrm{cos}\lambda t \end{array}\right)\left(\begin{array}{c} \hat{A}\\ \hat{B} \end{array}\right),$$

if and only if

6 $$\left(\begin{array}{c} \left[\hat{H},\hat{A}\right]\\ \left[\hat{H},\hat{B}\right] \end{array}\right)=\left(\begin{array}{cc} 0 & i\lambda \\ -i\lambda & 0 \end{array}\right)\left(\begin{array}{c} \hat{A}\\ \hat{B} \end{array}\right).$$

For this analysis, it is important to note that the 2×2 matrix in Eq. ([Disp-formula Ch1.E5]) is the exponential of the 2×2 matrix in Eq. ([Disp-formula Ch1.E6]) multiplied by -it (see Sect. S2 in the Supplement):

7 $$\left(\begin{array}{cc} \mathrm{cos}\lambda t & \mathrm{sin}\lambda t\\ -\mathrm{sin}\lambda t & \mathrm{cos}\lambda t \end{array}\right)=\mathrm{exp}\left(\begin{array}{cc} 0 & \lambda t\\ -\lambda t & 0 \end{array}\right).$$

Obviously, for this case, it is possible to take a particular parameter (λ) of a system of commutator equations and insert it into the template Eq. ([Disp-formula Ch1.E2]), which is formulated as a set of two propagation formulae. Then the following questions arise:

1. Is it possible to apply this procedure to more complex cases, such as that of Eq. ([Disp-formula Ch1.E4])?
2. To what dimension can we reduce a given problem?
3. Are there any template propagation formulae for the case where a reduction to a 3-D or 4-D case is possible?

As a final remark in this subsection, we note that we have performed these calculations in the operator space (Liouville space). This seems advantageous because it is the natural way of treating this topic. We describe states in terms of operators rather than wave functions (see above). Some properties of the Liouville space that are important for this work are listed in Sect. [Sec Ch1.S2.SS2].

### 2.2 Properties of the Liouville space that are important in this article

In some papers, the space of wave functions is denoted as the Hilbert space in order to have a contrast with the Liouville space. However, this does not seem appropriate since the space of linear operators also satisfies the conditions of being a Hilbert space [Bibr bib1.bibx9].

- Definition of the *scalar product* of two operators $\hat{A}$ and $\hat{B}$ : $\left(\hat{A},\hat{B}\right)\;:=\text{Tr}\left(A^{†}\cdot B\right)$ , where A and B are the matrix representations of $\hat{A}$ and $\hat{B}$ in the wave-function space and the superscript † means the Hermitian conjugate of the corresponding matrix.
- Two operators $\hat{A}$ and $\hat{B}$ are said to be *orthogonal* if their scalar product is 0: $\hat{A}⟂\hat{B}\;\Leftrightarrow \;\left(\hat{A},\hat{B}\right)=0$ .
- The Euclidean *norm* of an operator is defined as the square root of the scalar product of the operator with itself: $∥\hat{A}∥=\sqrt{\left(\hat{A},\hat{A}\right)}=\sqrt{\text{Tr}\left(A^{†}\cdot A\right)}$ . For the sake of brevity, the term “norm” is used throughout this paper to refer to the Euclidean norm. The rules for calculating the norm values for some of the operators used in this article are given in Appendix A. The norm of an operator is invariant with respect to a unitary transformation, but it depends on the relevant space, which is different for different numbers of spins. Therefore, the table in Appendix A contains different norm equations for the same operator but in different spaces.
- Each operator of this space can be *expanded* into a series of basis operators. In particular, for the density operator ρ , we have
8 $$\hat{\rho }=\rho_{1}{\hat{u}}_{1}+\ldots +\rho_{d}{\hat{u}}_{d}=u^{T}\cdot \rho ,$$
  where d is the dimension of the particular Liouville space, ρ is a column matrix which contains the expansion coefficients $\rho_{i}\;(i\in \{1\ldots d\})$ of the density operator, u is a column matrix whose elements are the basis operators, and $A^{T}$ is the transpose of the matrix A . If all the basis operators are pairwise orthogonal, $\rho_{i}$ can be calculated as follows:
9 $$\rho_{i}=\left(\hat{\rho },{\hat{u}}_{i}\right)/∥{\hat{u}}_{i}∥\;\;\left(i\in \{1\ldots d\}\right).$$
- Mappings between operators are described by *superoperators*:
  i. Liouville superoperator (or simply “Liouvillian”) for forming the commutator with the Hamiltonian: $\hat{\hat{L}}$ : $\hat{A}\mapsto \left[\hat{H},\hat{A}\right]$ , also written as $\hat{\hat{L}}\hat{A}=\left[\hat{H},\hat{A}\right]$ ,and
  ii. propagation superoperator (or simply “superpropagator”) for describing the time evolution: $\hat{\hat{U}}$ : $\hat{\rho }(0)\mapsto \hat{\rho }(t)$ , also written as
10 $$\hat{\rho }(t)=\hat{\hat{U}}\hat{\rho }(0).$$
- The time evolution of the density operator is governed by the *Liouville–von Neumann equation*. It is formulated in the Liouville space as
11 $$\frac{d}{dt}\hat{\rho }=-i\hat{\hat{L}}\hat{\rho }.$$
  If $\hat{\hat{L}}$ does not depend on time, the formal solution is
12 $$\hat{\rho }(t)=\hat{\hat{U}}(t)\hat{\rho }(0)\;\;\text{with}\;\;\hat{\hat{U}}(t)=\mathrm{exp}\left(-i\hat{\hat{L}}t\right).$$
  Here we find a similarity to the matrix formulation of the POF (Eqs. [Disp-formula Ch1.E5]–[Disp-formula Ch1.E7]). In fact, the coefficient matrix of the system of commutator equations is the transposition of the Liouvillian matrix, and the coefficient matrix of the POF (Eq. [Disp-formula Ch1.E5]) is the transposition of the superpropagator. This is proven in the Supplement (Sect. S1.1).
- The norm of the density matrix is time-invariant. This can be proven by multiplying the Liouville–von Neumann Eq. ([Disp-formula Ch1.E11]) by $\hat{\rho }$ :
13 $$\left(\hat{\rho }\;.\;\frac{d}{dt}\hat{\rho }\right)=\frac{1}{2}\frac{d}{dt}\left(\hat{\rho }\;.\;\hat{\rho }\right)=-i\left(\hat{\rho }\;.\;\hat{\hat{L}}\hat{\rho }\right)=0.$$
  The scalar product of a Hermitian operator ( $\hat{\rho }$ ) and its commutator with another Hermitian operator ( $\hat{H}$ ) is 0 (Sect. S1.2).

The Liouville space formulation objective of this article is to find a subspace of the total Liouville space that contains all possible states occurring in the current problem and that has the smallest possible dimension. An analogous procedure is not possible in the wave-function space, where the time evolution has to be calculated using $\hat{U}(t)\hat{\rho }(0){\hat{U}}^{-1}(t)$ . In the following section, commutator equations representing the action of the Liouvillian are established. Their coefficients are needed for further calculation of the propagation formulae.

### 2.3 Two forms and two bases for symbolic description of the time evolution

For practical calculations, Eq. ([Disp-formula Ch1.E10]) is usually transformed into a matrix equation. In the literature this is usually done in two different forms, both of which can be found, e.g., in [Bibr bib1.bibx5] (Sect. 2.1.4).

*Form 1*:

14 ρ(t)=U(t)⋅ρ(0).

The time evolution is described here in the space of N -dimensional column matrices: ρ(t) is the density matrix according to Eq. ([Disp-formula Ch1.E8]), i.e., a column containing the coefficients of the expansion of the density operator $\hat{\rho }(t)$ into basis operators. That is, we follow the propagation into the space $C^{N}$ , i.e., the space of N -row column matrices with the basis $\left\{(1,0,\ldots ,0{)}^{T},(0,1,\ldots ,0{)}^{T},\ldots ,(0,0,\ldots ,1{)}^{T}\right\}$ . The superpropagator $\hat{\hat{U}}$ is an isomorphism in this space and can be represented by the N×N matrix U .

*Form 2* (denoted here as the “propagation formula”):

15 $$\begin{array}{l} {\hat{A}}_{1}\overset{\hat{H}t}{⟶}V_{11}{\hat{A}}_{1}+V_{12}{\hat{A}}_{2}+\ldots +V_{1N}{\hat{A}}_{N},\\ {\hat{A}}_{2}\overset{\hat{H}t}{⟶}V_{21}{\hat{A}}_{1}+V_{22}{\hat{A}}_{2}+\ldots +V_{2N}{\hat{A}}_{N},\\ \ldots \\ {\hat{A}}_{N}\overset{\hat{H}t}{⟶}V_{N1}{\hat{A}}_{1}+V_{N2}{\hat{A}}_{2}+\ldots +V_{NN}{\hat{A}}_{N}. \end{array}$$

This means that the density operator, which is ${\hat{A}}_{1}$ at t = 0, evolves under the influence of the Hamiltonian $\hat{H}$ during time t into a linear combination of independent operators ${\hat{A}}_{1},\;{\hat{A}}_{2},\ldots ,{\hat{A}}_{N}$ . Unlike form 1, here we work in the operator space with the basis $\left\{{\hat{A}}_{1},{\hat{A}}_{2},\ldots ,{\hat{A}}_{N}\right\}$ .

*Connection between both forms.* $U=V^{T}$ if the basis column matrices (form 1) are assigned to the corresponding basis operators of the second basis. For proof, see Sect. S1.1.

This means that we can rewrite Eq. ([Disp-formula Ch1.E15]) as

16 $$\begin{array}{l} {\hat{A}}_{1}\overset{\hat{H}t}{⟶}U_{11}{\hat{A}}_{1}+U_{21}{\hat{A}}_{2}+\ldots +U_{N1}{\hat{A}}_{N},\\ {\hat{A}}_{2}\overset{\hat{H}t}{⟶}U_{12}{\hat{A}}_{1}+U_{22}{\hat{A}}_{2}+\ldots +U_{N2}{\hat{A}}_{N},\\ \ldots \\ {\hat{A}}_{N}\overset{\hat{H}t}{⟶}U_{1N}{\hat{A}}_{1}+U_{2N}{\hat{A}}_{2}+\ldots +U_{NN}{\hat{A}}_{N}. \end{array}$$

The set of all U values is the dual space of the set of all V . Similarly, the coefficient matrix of the commutator equations is the transposed Liouvillian matrix (see Sect. S1.1).

## 3 Procedure for finding propagation formulae

### 3.1 Requirements for a suitable subspace

To be sure that a given subspace S contains the whole evolution of a spin system, we have to check that the action of the propagator $\hat{\hat{U}}$ on each operator $\hat{A}$ of this subspace also results in an element of the subspace:

17 $$\forall \hat{A}\in S\;:\;\hat{\hat{U}}\;\hat{A}\in S.$$

A subspace with this property is said to be propagator-invariant [Bibr bib1.bibx9]. This is equivalent to the Liouvillian invariance of the subspace, i.e.,

18 $$\forall \hat{A}\in S\;:\;\hat{\hat{L}}\;\hat{A}\in S,$$

because the propagator is the sum of repeated $\hat{\hat{L}}$ actions:

19 $$\hat{\hat{U}}\hat{A}=\mathrm{exp}\left(-i\hat{\hat{L}}t\right)\hat{A}=\sum_{n=0}^{\infty }\frac{(-it{)}^{n}}{n!}{\hat{\hat{L}}}^{n}\;\hat{A}\;\in S.$$

A sufficient condition for a subspace spanned by ${\hat{u}}_{1}\;\ldots \;{\hat{u}}_{N}$ (Eq. [Disp-formula Ch1.E8]) to be Liouvillian-invariant is that the action of the Liouvillian of any of the ${\hat{u}}_{i}$ values also results in an element of that subspace, i.e., $\hat{\hat{L}}{\hat{u}}_{i}\in S\;\forall i\in \{1\ldots N\}$ . A Liouvillian-invariant subspace which is of interest here should at least contain the initial density operator. Furthermore, all multiple actions of the Liouvillian on the density operator must result in elements of that subspace: ${\hat{\hat{L}}}^{n}\;\hat{\rho }(0)\in S\;\forall n\in N$ .

In principle we can construct such a subspace as the set of all linear combinations of $\hat{\rho }(0)$ , $\hat{\hat{L}}\hat{\rho }(0)$ , ${\hat{\hat{L}}}^{2}\hat{\rho }(0),\ldots ,{\hat{\hat{L}}}^{N}\hat{\rho }(0)$ , where N is the largest number for which this operator set is linearly independent. This means that ${\hat{\hat{L}}}^{N+1}\hat{\rho }(0)$ can be represented as a linear combination of all lower powers. Then, all further applications of $\hat{\hat{L}}$ lead to operators which are also linearly dependent.

This procedure is reminiscent of the formation of Krylov subspaces in matrix spaces [Bibr bib1.bibx26]. For further considerations in this article, in particular the calculation of matrices, it is convenient to have basis operators that are pairwise orthogonal. This can be achieved by combining the Krylov-like procedure with the Gram–Schmidt orthogonalization, which is analogous to the Arnoldi procedure for matrix spaces [Bibr bib1.bibx26]. The details of the procedure are presented in the next subsection.

### 3.2 First step: creating a closed system of commutator equations

According to the result of the previous subsection, the application of the Liouvillian to the initial density operator is repeated as long as the result contains a component that is linearly independent of all previous results. The action of the Liouvillian consists in forming the commutator of the Hamiltonian with the considered operator. Consequently, the search for this Liouvillian-invariant minimum subspace is performed by repeatedly calculating the commutator of the Hamiltonian using the operator representing the initial state of the spin system, as shown in detail below.

Let us assume that the system under consideration is characterized by the Hamiltonian $\hat{H}$ and the initial state density operator by ${\hat{A}}_{1}$ .

*Evaluation of the first commutator*: $\left[\hat{H},{\hat{A}}_{1}\right]$

If the result is 0, the state of the spin system is constant in time; see the Supplement (examples 0D-1 and 0D-2). In the case where this commutator does not vanish, it can be decomposed into a term which is proportional to ${\hat{A}}_{1}$ and another term ${\hat{A}}_{12}$ which is orthogonal to the first one:

20 $$\left[\hat{H},{\hat{A}}_{1}\right]=\lambda_{11}{\hat{A}}_{1}+{\hat{A}}_{12}\;\;\text{with}\;\;{\hat{A}}_{12}⟂{\hat{A}}_{1}.$$

The commutator of two Hermitian operators is always orthogonal to both, as proven in the Supplement. This means that, for a Hermitian ${\hat{A}}_{1}$ , $\left[\hat{H},{\hat{A}}_{1}\right]$ cannot have a component that contains ${\hat{A}}_{1}$ itself. This is different for non-Hermitian operators (see Sect. [Sec Ch1.S4.SS1]).

We replace ${\hat{A}}_{12}\to \lambda_{12}{\hat{A}}_{2}$ , choosing the scalar $\lambda_{12}$ so that ${\hat{A}}_{2}$ has the same norm as ${\hat{A}}_{1}$ . The corresponding table in Appendix A can be used to determine the norms of the operators.

*Evaluation of the second commutator*: $\left[\hat{H},{\hat{A}}_{2}\right]$

This will be decomposed as

21 $$\left[\hat{H},{\hat{A}}_{2}\right]=\lambda_{21}{\hat{A}}_{1}+\lambda_{22}{\hat{A}}_{2}+\lambda_{23}{\hat{A}}_{3},$$

with the condition that ${\hat{A}}_{3}$ is orthogonal to both ${\hat{A}}_{1}$ and ${\hat{A}}_{2}$ . The coefficient $\lambda_{23}$ is chosen so that ${\hat{A}}_{3}$ has the same norm as ${\hat{A}}_{1}$ and ${\hat{A}}_{2}$ .

If ${\hat{A}}_{3}$ = 0, i.e., $\left[\hat{H},{\hat{A}}_{2}\right]$ is a linear combination of ${\hat{A}}_{1}$ and ${\hat{A}}_{2}$ , the procedure is finished. Then we have a system of commutator equations like Eq. ([Disp-formula Ch1.E3]), i.e., the usual POF.

*Evaluation of the nth commutator*: $\left[\hat{H},{\hat{A}}_{n}\right]$

Similarly, it will be expanded into a series of operators known from the previous commutator evaluations and a remainder:

22 $$\left[\hat{H},{\hat{A}}_{n}\right]=\lambda_{n1}{\hat{A}}_{1}+\ldots +\lambda_{n,n}{\hat{A}}_{n}+\lambda_{n,n+1}{\hat{A}}_{n+1},$$

with the condition that ${\hat{A}}_{n+1}$ is orthogonal to all of the ${\hat{A}}_{k}$ values with k∈1…n . Again, the coefficient $\lambda_{n,n+1}$ is chosen so that ${\hat{A}}_{n+1}$ has the same norm as the other operators of this set.

*End of the procedure.* If for a certain n = N the commutator is a linear combination of the previously determined ${\hat{A}}_{k}$ without any remainder, the iteration is finished. The set of pairwise orthogonal operators $\left\{{\hat{A}}_{1}\ldots {\hat{A}}_{N}\right\}$ spans a Liouvillian-invariant subspace of the entire Liouville space. Its dimension is N .

*Possible modification of the procedure.* The remainder of any commutator evaluation can be written as the sum of two or more operators, all of which must be orthogonal to the other operators. In some cases, this can simplify the coefficient matrix.

### 3.3 Second step: Liouvillian matrix

The result of the whole procedure is a system of equations like the following:

23 $$\begin{array}{rl} \left(\begin{array}{c} \left[\hat{H},{\hat{A}}_{1}\right]\\ \left[\hat{H},{\hat{A}}_{2}\right]\\ \left[\hat{H},{\hat{A}}_{3}\right]\\ \ldots \\ \left[\hat{H},{\hat{A}}_{N-2}\right]\\ \left[\hat{H},{\hat{A}}_{N-1}\right]\\ \left[\hat{H},{\hat{A}}_{N}\right] \end{array}\right)= & \;\left(\begin{array}{ccccccc} \lambda_{11} & \lambda_{12} & 0 & \ldots & 0 & 0 & 0\\ \lambda_{21} & \lambda_{22} & \lambda_{23} & \ldots & 0 & 0 & 0\\ 0 & \lambda_{32} & \lambda_{33} & \ldots & 0 & 0 & 0\\ \ldots & \ldots & \ldots & \ldots & \ldots & \ldots & \ldots \\ 0 & 0 & 0 & \ldots & \lambda_{N-2,N-2} & \lambda_{N-2,N-1} & 0\\ 0 & 0 & 0 & \ldots & \lambda_{N-1,N-2} & \lambda_{N-1,N-1} & \lambda_{N-1,N}\\ 0 & 0 & 0 & \ldots & 0 & \lambda_{N,N-1} & \lambda_{N,N} \end{array}\right)\\ & \;\left(\begin{array}{c} {\hat{A}}_{1}\\ {\hat{A}}_{2}\\ {\hat{A}}_{3}\\ \ldots \\ {\hat{A}}_{N-2}\\ {\hat{A}}_{N-1}\\ {\hat{A}}_{N} \end{array}\right). \end{array}$$

As shown in Sect. S1.1, the coefficient matrix is the transposition of the Liouvillian matrix. This system of equations implies that the action of the Liouvillian on any ${\hat{A}}_{i}\;(i\in \{1\ldots N\})$ leads to a linear combination of ${\hat{A}}_{i}$ . In other words, the subspace spanned by the operators ${\hat{A}}_{1}\;\ldots \;{\hat{A}}_{n}$ is both Liouvillian-invariant and propagator-invariant. The density operator, once located in this subspace, will not leave it as long as the interaction does not vary. This explains why the POF can be applied successfully as a 2-D problem, even if the complete Liouville space has a much higher dimension.

The zeros in the upper triangle of the coefficient matrix result from the above: if the remainder of the n th commutator is identified with only one new operator, then $\lambda_{n,n+1}$ and ${\hat{A}}_{n+1}$ are determined. $\lambda_{n,n+2},\ldots$ and ${\hat{A}}_{n+2},\ldots$ are still unknown in this step; the matrix elements to the right of them remain zero. In the case of the modified procedure mentioned above, the matrix structure in Eq. ([Disp-formula Ch1.E23]) changes. Then, $\lambda_{n,n+2}\ldots$ can also be nonzero. Due to the hermiticity of the Liouvillian, i.e., $L_{ij}=L_{ji}^{*}$ , the lower triangle matrix has the same pattern of zeros as the upper one. The coefficient matrix has a band structure for the unmodified version of the procedure.

When the basis operators are Hermitian, all elements are purely imaginary. This means that all elements of the main diagonal must be zero. However, if the basis contains non-Hermitian operators, the main diagonal will contain nonzero elements, but they must be real numbers.

### 3.4 Third step: estimation of the propagator matrix and propagation rules

In principle, the propagator matrix can be obtained by evaluating the matrix exponential according to Eq. ([Disp-formula Ch1.E12]):

24 $$U=\mathrm{exp}\left(-iLt\right)=\left(\begin{array}{cccc} U_{11} & U_{12} & \ldots & U_{1N}\\ U_{21} & U_{22} & \ldots & U_{2N}\\ \ldots & \ldots & \ldots & \ldots \\ U_{N1} & U_{N2} & \ldots & U_{NN} \end{array}\right).$$

L is a pure imaginary matrix for a Hermitian operator basis. In this case, U only contains real elements and is orthogonal. This means that the norms of all the rows and columns of this matrix are unity:

25 $$\sum_{i=1}^{N}U_{ij}^{2}=\sum_{j=1}^{N}U_{ij}^{2}=1.$$

The propagation rules can be obtained from the elements of the transposed propagator matrix (for proof, see Sect. S1.1):

26 $$\begin{array}{c} {\hat{A}}_{1}\overset{\hat{H}t}{⟶}{\hat{A}}_{1}\;U_{11}+{\hat{A}}_{2}\;U_{21}+\ldots +{\hat{A}}_{N}\;U_{N1},\\ {\hat{A}}_{2}\overset{\hat{H}t}{⟶}{\hat{A}}_{1}\;U_{12}+{\hat{A}}_{2}\;U_{22}+\ldots +{\hat{A}}_{N}\;U_{N2},\\ \ldots \end{array}$$

However, it is not necessary to recompute the matrix exponential for each new situation. Instead, for low-dimensional subspaces, the generic propagation formulae shown in the next section can be used as a template. Here the elements of the Liouvillian matrix have to be inserted directly, without the need to perform the matrix exponentialization. This was done above for the POF example, where the constant λ resulting from the commutator equations could be used directly as the oscillation frequency.

## 4 Special cases

The situations in the examples shown below are characterized by different initial states and different Hamiltonians. The Hamiltonians are listed in Appendix B.

A detailed analytical consideration of each example can be found in the Supplement.

### 4.1 Reduction to a 1-D subspace

In this case, the commutator of the Hamiltonian with the density operator at t=0 , i.e., ${\hat{A}}_{1}$ , is proportional to ${\hat{A}}_{1}$ itself,

27 $$\left[\hat{H},{\hat{A}}_{1}\right]=\lambda {\hat{A}}_{1},$$

and only occurs if ${\hat{A}}_{1}$ is non-Hermitian, e.g., ${\hat{A}}_{1}={\hat{I}}_{+}\equiv {\hat{I}}_{x}+i{\hat{I}}_{y}$ as used for the characterization of the complex free induction decay (FID) [Bibr bib1.bibx1]. Then the coefficient matrix only consists of one scalar λ . The propagator is also a scalar:

28 $$U_{1D}=e^{-i\lambda t}.$$

The corresponding propagation rule is

29 $${\hat{A}}_{1}\overset{\hat{H}t}{⟶}{\hat{A}}_{1}e^{-i\lambda t}.$$

*Example.* A rotating frame, resonance offset Δω , and complex transversal magnetization, which is represented by ${\hat{I}}_{+}$ :

30 $${\hat{I}}_{+}\overset{-\Delta \omega {\hat{I}}_{z}\;t}{⟶}\;\;\;{\hat{I}}_{+}e^{-i\Delta \omega t}.$$

### 4.2 Case of reduction to a 2-D subspace

The corresponding equations are like Eqs. ([Disp-formula Ch1.E3]) and ([Disp-formula Ch1.E6]) and belong to the POF. The Liouvillian matrix and the propagator matrix are the transpositions of the matrices in Eqs. ([Disp-formula Ch1.E5]) and ([Disp-formula Ch1.E6]):

31 $$\begin{array}{l} L_{2D}=\left(\begin{array}{cc} 0 & -i\lambda \\ i\lambda & 0 \end{array}\right),\\ U_{2D}=\mathrm{exp}\left(-iL_{2D}t\right)=\left(\begin{array}{cc} \mathrm{cos}\lambda t & -\mathrm{sin}\lambda t\\ \mathrm{sin}\lambda t & \mathrm{cos}\lambda t \end{array}\right). \end{array}$$

The propagation formulae are given by Eq. ([Disp-formula Ch1.E2]), which describes an oscillatory behavior between the initial state and another state described by the commutator of the Hamiltonian, with the operator corresponding to the density operator at the beginning.

In addition to the cases known from numerous POF applications, there are other situations that can be described as time evolution in a 2-D subspace. All of them are well known; they are listed here for the sake of completeness:

- FID of an ensemble of isolated pairs of equal spins ( $I_{1}$ , $I_{2}$ ) after a π/2 pulse and homonuclear dipolar interaction within the spin pairs; we observe the transversal magnetization represented by the operator sum ${\hat{I}}_{1x}+{\hat{I}}_{2x}$ :
32 $$\begin{array}{rl} {\hat{I}}_{1x} & \;+{\hat{I}}_{2x}\overset{{\hat{H}}_{\mathrm{II}}\;t}{⟶}\left({\hat{I}}_{1x}+{\hat{I}}_{2x}\right)\mathrm{cos}\frac{3}{2}D_{\mathrm{II}}t\\ & \;\;\;\;-2\left({\hat{I}}_{1z}{\hat{I}}_{2y}+{\hat{I}}_{1y}{\hat{I}}_{2z}\right)\mathrm{sin}\frac{3}{2}D_{\mathrm{II}}t. \end{array}$$
- FID of an ensemble of isolated pairs of nonequal spins ( I,S ) after a π/2 pulse in the I channel and heteronuclear dipolar interaction within the spin pairs; we observe the transversal I magnetization represented by the operator ${\hat{I}}_{x}$ :
33 $${\hat{I}}_{x}\;\overset{{\hat{H}}_{\mathrm{IS}}\;t}{⟶}\;{\hat{I}}_{x}\mathrm{cos}D_{\mathrm{IS}}t-2{\hat{I}}_{y}{\hat{S}}_{z}\mathrm{sin}D_{\mathrm{IS}}t.$$
- FID of an ensemble of spins I=1 (e.g., ${}^{2}H$ or ${}^{14}N$ ) under quadrupolar interaction; we again follow the transversal magnetization:
34 $${\hat{I}}_{x}\;\overset{{\hat{H}}_{Q}\;t}{⟶}\;{\hat{I}}_{x}\mathrm{cos}\omega_{Q}t+\left({\hat{I}}_{z}{\hat{I}}_{y}+{\hat{I}}_{y}{\hat{I}}_{z}\right)\mathrm{sin}\omega_{Q}t.$$
- Ensemble of pairs of homonuclearly coupled equal spins ( $I_{1}$ , $I_{2}$ ) with spin quantum number 1/2 , where initially spin 1 is oriented parallel to $B_{0}$ and spin 2 is oriented antiparallel to that. We follow the difference z magnetization, which is represented by ${\hat{I}}_{1z}-{\hat{I}}_{2z}$ :
35 $$\begin{array}{rl} {\hat{I}}_{1z} & \;-{\hat{I}}_{2z}\;\overset{{\hat{H}}_{\mathrm{II}}\;t}{⟶}\;\left({\hat{I}}_{1z}-{\hat{I}}_{2z}\right)\mathrm{cos}D_{\mathrm{II}}t\\ & \;\;\;\;+2\left({\hat{I}}_{1x}{\hat{I}}_{2y}-{\hat{I}}_{1y}{\hat{I}}_{2x}\right)\mathrm{sin}D_{\mathrm{II}}t. \end{array}$$
- Cross-polarization within pairs of antiparallel nonequal spins ( I , S ): both spins are locked in resonant rf fields with equal nutation frequencies $\omega_{1I}=\omega_{1S}\gg D_{\mathrm{IS}}$ (Hartmann–Hahn (HH) condition). The Hamiltonian and the state operators are given in the doubly rotating frame following [Bibr bib1.bibx7], where the z direction is along the rf irradiation. If initially the S spins are oriented parallel to the locking field and the I spins are antiparallel to that, the time evolution can be described by following ${\hat{S}}_{z}-{\hat{I}}_{z}$ :
36 $$\begin{array}{rl} {\hat{S}}_{z} & \;-{\hat{I}}_{z}\overset{{\hat{H}}_{\mathrm{HH}}\;t}{⟶}\;\left({\hat{S}}_{z}-{\hat{I}}_{z}\right)\mathrm{cos}D_{\mathrm{IS}}t\\ & \;\;\;\;\;\;+2\left({\hat{I}}_{x}{\hat{S}}_{y}-{\hat{I}}_{y}{\hat{S}}_{x}\right)\mathrm{sin}D_{\mathrm{IS}}t. \end{array}$$
  The last equation describes the behavior of the difference between I and S polarizations, not the individual polarizations themselves. The time evolution of the latter requires at least a 3-D approach (see below).

The oscillation takes place in the first three examples between observable transversal magnetization and antiphase states, in the last two examples between longitudinal difference magnetization and zero and double-quantum coherences. These examples show an effect of the dimension reduction: to obtain a 2-D problem, the operators characterizing the states of the spin system have a more complicated structure than in the simple cases above. For example, it would be possible to consider the right-hand side of Eq. ([Disp-formula Ch1.E32]) to be a linear combination of the four states ${\hat{I}}_{1x}$ , ${\hat{I}}_{2x}$ , $2{\hat{I}}_{1z}{\hat{I}}_{2y}$ , and $2{\hat{I}}_{1y}{\hat{I}}_{2z}$ if a more illustrative notation is desired.

### 4.3 Case of reduction to a 3-D subspace

#### 4.3.1 Generic notation

Here we deal with those cases where the procedure described above reaches the cancellation condition after three commutator equations of the forms

37 $$\begin{array}{l} \left[\hat{H},\hat{A}\right]=\;\;\;\;ia\hat{B},\\ \left[\hat{H},\hat{B}\right]=-ia\hat{A}+ib\hat{C},\\ \left[\hat{H},\hat{C}\right]=-ib\hat{B}, \end{array}$$

where a , b∈R . In step 2, we determine the Liouvillian matrix as the transposed coefficient matrix of Eq. ([Disp-formula Ch1.E37]):

38 $$L_{3D}=\left(\begin{array}{ccc} 0 & -ia & 0\\ ia & 0 & -ib\\ 0 & ib & 0 \end{array}\right).$$

From this we determine the matrix of the superpropagator as the matrix exponential corresponding to Eq. ([Disp-formula Ch1.E24]):

39 $$\begin{array}{rl} U_{3D} & \;=\mathrm{exp}\left(-iL_{3D}t\right)\\ & \;=\frac{1}{q^{2}}\left(\begin{array}{ccc} b^{2}+a^{2}\mathrm{cos}qt & -aq\mathrm{sin}qt & ab(1-\mathrm{cos}qt)\\ aq\mathrm{sin}qt & q^{2}\mathrm{cos}qt & -bq\mathrm{sin}qt\\ ab(1-\mathrm{cos}qt) & bq\mathrm{sin}qt & a^{2}+b^{2}\mathrm{cos}qt \end{array}\right), \end{array}$$

with $q^{2}\;:=\;a^{2}+b^{2}$ . The orthogonality of $U_{3D}$ , i.e., the validity of Eq. ([Disp-formula Ch1.E25]), can be verified immediately.

In step (3), according to Eq. ([Disp-formula Ch1.E26]), the following propagation rules are obtained from the columns of $U_{3D}$ :

40A^⟶H^tA^⋅b2+a2cos⁡qtq2+B^⋅aqsin⁡qt+C^⋅abq2(1-cos⁡qt),41B^⟶H^tB^⋅cos⁡qt+C^⋅bqsin⁡qt-A^⋅aqsin⁡qt,42C^⟶H^tC^⋅a2+b2cos⁡qtq2+A^⋅abq2(1-cos⁡qt)-B^⋅bqsin⁡qt,

This can be seen as an extension of the POF to 3-D problems.

If all the basis operators are Hermitian, one of the eigenvalues of $L_{3D}$ is zero because of $\text{det}L_{3D}=0$ . Therefore, the solution of the Liouville–von Neumann equation may contain a nonzero constant beyond the oscillating terms. The propagator-matrix element ${\left(U_{3D}\right)}_{11}$ contains the time evolution of the initial state $\hat{A}$ . The constant term $b^{2}/(a^{2}+b^{2})$ shows that the oscillations do not take place around zero as in the 2-D case, but around another level. Moreover, its amplitude is reduced to $a^{2}/(a^{2}+b^{2})$ , while the frequency increases more the smaller the amplitude is (Fig. [Fig Ch1.F1]).

**Figure 1 Ch1.F1:**
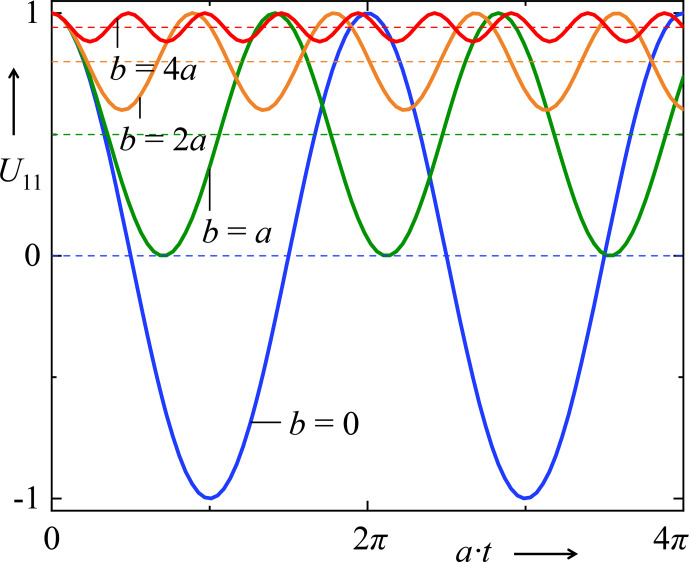
Time evolution of the prefactor of $\hat{A}$ in Eq. (40) for different ratios b/a . The particular case b=0 leads to the 2-D case; therefore, the corresponding curve is a pure oscillation around 0. With increasing b , the average levels of the related oscillations (dashed horizontal lines) increase, whereas the corresponding amplitudes decrease.

There is one important special case: a=b . Here the propagation formulae are simplified to

43A^⟶H^tA^⋅cos⁡2qt2+B^⋅12sin⁡qt+C^⋅sin⁡2qt2,44B^⟶H^tB^⋅cos⁡qt+C^⋅12sin⁡qt-A^⋅12sin⁡qt,45C^⟶H^tC^⋅cos⁡2qt2+A^⋅sin⁡2qt2-B^⋅12sin⁡qt.

This is applied in the description of cross-polarization and polarization transfer (see the corresponding examples below).

#### 4.3.2 Group 1 of experiments leading to 3-D subspaces: magnetization initially aligned parallel to $B_{0}$

- *Off-resonance nutation.* This involves rf irradiation with strength $\omega_{1}$ on an ensemble of isolated spins under resonance offset Δω :
46 $$\begin{array}{rl} {\hat{I}}_{z} & \;\overset{\left({\hat{H}}_{\mathrm{Ix}}+{\hat{H}}_{\Delta }\right)\;t}{⟶}\;{\hat{I}}_{z}\cdot \frac{\Delta \omega^{2}+\omega_{1}^{2}\mathrm{cos}qt}{q^{2}}\\ & \;\;+{\hat{I}}_{y}\cdot \frac{\omega_{1}}{q}\mathrm{sin}qt+{\hat{I}}_{x}\cdot \frac{\omega_{1}\Delta \omega }{q^{2}}(1-\mathrm{cos}qt), \end{array}$$
  with $q^{2}$ = $\omega_{1}^{2}+\Delta \omega^{2}$ .
- *Nutation and dipolar interaction.* The observed spin I , which is heteronuclearly coupled to the spin S , is additionally irradiated with rf of strength $\omega_{1I}$ :
47 $$\begin{array}{rl} {\hat{I}}_{z} & \;\overset{\left({\hat{H}}_{\mathrm{Ix}}+{\hat{H}}_{\mathrm{IS}}\right)\;t}{⟶}\;{\hat{I}}_{z}\cdot \frac{D^{2}+\omega_{1I}^{2}\mathrm{cos}qt}{q^{2}}\\ & \;\;+{\hat{I}}_{y}\cdot \frac{\omega_{1I}}{q}\mathrm{sin}qt+2{\hat{I}}_{x}{\hat{S}}_{z}\cdot \frac{\omega_{1I}D}{q^{2}}(1-\mathrm{cos}qt), \end{array}$$
  with $q^{2}\;=\;\omega_{1I}^{2}+D^{2}$ .

(The case of *homonuclear* interaction under rf irradiation leads to a 4-D problem; see below.)

The similarity of the two Eqs. ([Disp-formula Ch1.E46]) and ([Disp-formula Ch1.E47]) is obvious. For Δω→0 and $D_{\mathrm{IS}}\to 0$ , respectively, they merge into an equation describing a rotation in the y – z plane. Both equations reflect the well-known fact that a total inversion of the magnetization with a single rectangular pulse is only possible if the offset or the coupling is zero. In addition, the coupling and resonance offset change both the pulse duration $\tau_{\pi /2}$ required to reach maximum y magnetization and the pulse duration $\tau_{\pi }$ required to reach zero y magnetization to shorter times:

48 $$\tau_{\pi /2}=\frac{\pi }{2\sqrt{\omega_{1I}^{2}+C^{2}}};\;\;\;\tau_{\pi }=\frac{\pi }{\sqrt{\omega_{1I}^{2}+C^{2}}},$$

with C=Δω for the off-resonance nutation (Eq. [Disp-formula Ch1.E46]) and $C=D_{\mathrm{IS}}$ for the nutation under heteronuclear dipolar interaction.

#### 4.3.3 Group 2 of experiments leading to 3-D subspaces: FID under both rf irradiation and dipolar interaction

- *Decoupling* experiment: this involves an ensemble of spin pairs {IS} , heteronuclear dipolar interaction between I and S , rf irradiation on the S channel with finite rf power of strength $\omega_{1S}$ , and observations of spin I .
49 $$\begin{array}{rl} {\hat{I}}_{x} & \;\overset{\left({\hat{H}}_{\mathrm{Sx}}+{\hat{H}}_{\mathrm{IS}}\right)\;t}{⟶}\;{\hat{I}}_{x}\cdot \frac{\omega_{1S}^{2}+D_{\mathrm{IS}}^{2}\mathrm{cos}qt}{q^{2}}\\ & \;-2{\hat{I}}_{y}{\hat{S}}_{z}\;\cdot \;\frac{D_{\mathrm{IS}}}{q}\mathrm{sin}qt\\ & \;-2{\hat{I}}_{x}{\hat{S}}_{z}\cdot \frac{\omega_{1S}D_{\mathrm{IS}}}{q^{2}}(1-\mathrm{cos}qt), \end{array}$$
  with $q^{2}$ = $\omega_{1S}^{2}+D_{\mathrm{IS}}^{2}$ . To obtain the corresponding equation for the J coupling, replace $D_{\mathrm{IS}}$ with -πJ . This equation describes a *partial* exchange of polarization between x magnetization and two antiphase states.
  The decoupling effect is explained as follows: the observable part of the density operator – the prefactor of ${\hat{I}}_{x}$ – contains a constant part $\omega_{1S}^{2}/\left(\omega_{1S}^{2}+D_{\mathrm{IS}}^{2}\right)$ and an oscillating part with the amplitude $D_{\mathrm{IS}}^{2}/\left(\omega_{1S}^{2}+D_{\mathrm{IS}}^{2}\right)$ . Such oscillations were observed for instance in DIPSHIFT experiments [Bibr bib1.bibx13]. Powder-averaging leads to a rather fast decay of the oscillation, which gives a broad line (Pake doublet) after Fourier transformation, while the former gives a δ line. With increasing rf strength $\omega_{1S}$ , the prefactor of the broad peak decreases to zero for infinite rf power, while that of the δ line increases. The constant component is subject to relaxation damping and chemical-shift-induced oscillation on a longer timescale and produces a more or less narrow line.
- On-resonance *spin locking* and heteronuclear dipolar coupling:
50 $$\begin{array}{rl} {\hat{I}}_{x} & \;\overset{\left({\hat{H}}_{\mathrm{Ix}}+{\hat{H}}_{\mathrm{IS}}\right)\;t}{⟶}\;{\hat{I}}_{x}\cdot \frac{\omega_{1I}^{2}+D_{\mathrm{IS}}^{2}\mathrm{cos}qt}{q^{2}}\\ & \;-2{\hat{I}}_{y}{\hat{S}}_{z}\cdot \frac{D_{\mathrm{IS}}}{q}\mathrm{sin}qt\\ & \;+2{\hat{I}}_{z}{\hat{S}}_{z}\cdot \frac{\omega_{1I}D_{\mathrm{IS}}}{q^{2}}(1-\mathrm{cos}qt), \end{array}$$
  with $q^{2}$ = $\omega_{1I}^{2}+D_{\mathrm{IS}}^{2}$ . This oscillation frequency is indeed equal to that of the corresponding nutation experiment (Eq. [Disp-formula Ch1.E47]).
- On-resonance spin locking and homonuclear dipolar coupling:
51 $$\begin{array}{rl} {\hat{I}}_{1x} & \;+{\hat{I}}_{2x}\;\overset{\left({\hat{H}}_{\mathrm{Ix}}+{\hat{H}}_{\mathrm{II}}\right)\;t}{⟶}\left({\hat{I}}_{1x}+{\hat{I}}_{2x}\right)\cdot \frac{4\omega_{1I}^{2}+\frac{9}{4}D_{\mathrm{II}}^{2}\mathrm{cos}qt}{q^{2}}\\ & \;-2\left({\hat{I}}_{1y}{\hat{I}}_{2z}+{\hat{I}}_{1z}{\hat{I}}_{2y}\right)\cdot \frac{3D_{\mathrm{II}}}{2q}\mathrm{sin}qt\\ & \;+2\left({\hat{I}}_{1z}{\hat{I}}_{2z}-{\hat{I}}_{1y}{\hat{I}}_{2y}\right)\cdot \frac{3\omega_{1I}D_{\mathrm{II}}}{q^{2}}(1-\mathrm{cos}qt), \end{array}$$
  with $q^{2}$ → $4\omega_{1I}^{2}+\frac{9}{4}D_{\mathrm{IS}}^{2}$ .

Comments on the spin-locking examples (Eqs. [Disp-formula Ch1.E50] and [Disp-formula Ch1.E51]):

- Analogous to the other 3-D examples, the oscillation takes place around a level that increases with $\omega_{1I}$ . The latter corresponds to the spin-locked part of the transversal magnetization. At the same time, the amplitude of the oscillation is reduced.
- These oscillations are observed at the beginning of the spin-locking experiments [Bibr bib1.bibx10] and have been described theoretically by [Bibr bib1.bibx6] and [Bibr bib1.bibx17]. Due to their orientation dependence, they decay quite rapidly in a powder sample but can be refocused in MAS experiments.
- The propagation Eq. ([Disp-formula Ch1.E50]) is related to the same Hamiltonian as Eq. ([Disp-formula Ch1.E47]). As a consequence, the oscillation frequencies are the same. However, the different initial states lead to different subspaces and thus to different propagation formulae.

#### 4.3.4 Group 3 of experiments leading to 3-D subspaces: polarization transfer

The rf field strengths are assumed to be much larger than the corresponding coupling frequencies. This situation is very similar to the polarization transfer treated as a 2-D case. The difference lies in the initial states. Instead of the antiparallel orientation above, here one spin of the pairs is polarized and the other is not. The initial states are now only described by ${\hat{I}}_{1z}$ and ${\hat{S}}_{z}$ . Since these operators are not elements of the 2-D subspaces of the polarization difference examples above, the motion now takes place in other subspaces, which turn out to be 3-D.

- Equal spins:
52 $$\begin{array}{rl} {\hat{I}}_{1z} & \;\overset{{\hat{H}}_{\mathrm{II}}\;t}{⟶}\;{\hat{I}}_{1z}\cdot {\mathrm{cos}}^{2}\left(\frac{D_{\mathrm{II}}t}{2}\right)\\ & \;+\left({\hat{I}}_{1x}{\hat{I}}_{2y}+{\hat{I}}_{1y}{\hat{I}}_{2x}\right)\cdot \mathrm{sin}D_{\mathrm{II}}t+{\hat{I}}_{2z}\cdot {\mathrm{sin}}^{2}\left(\frac{D_{\mathrm{II}}t}{2}\right). \end{array}$$
- Pair of unequal spins I,S under the Hartmann–Hahn condition in the doubly rotating frame:
53 $$\begin{array}{rl} {\hat{S}}_{z} & \;\overset{{\hat{H}}_{\mathrm{HH}}\;t}{⟶}\;{\hat{S}}_{z}\cdot {\mathrm{cos}}^{2}\left(\frac{D_{\mathrm{IS}}t}{2}\right)\\ & \;+\left({\hat{I}}_{x}{\hat{S}}_{y}+{\hat{I}}_{y}{\hat{S}}_{x}\right)\cdot \mathrm{sin}D_{\mathrm{IS}}t+{\hat{I}}_{z}\cdot {\mathrm{sin}}^{2}\left(\frac{D_{\mathrm{IS}}t}{2}\right). \end{array}$$
  [Bibr bib1.bibx18] were the first to experimentally demonstrate this oscillatory exchange of polarization during cross-polarization.
- Depolarization of I spins in an ensemble of spin triples $\left\{I,S_{1},S_{2}\right\}$ under the Hartmann–Hahn condition in the doubly rotating frame: the coupling frequencies for the $I-S_{1}$ and $I-S_{2}$ interactions are $D_{1}$ and $D_{2}$ , respectively. The interaction between $S_{1}$ and $S_{2}$ is assumed to be zero. This can be realized experimentally by irradiating the S spins with a resonance offset which is $1/\sqrt{2}$ times the rf strength, known as the Lee–Goldburg condition [Bibr bib1.bibx14].
54 $$\begin{array}{rl} {\hat{I}}_{z} & \;\overset{{\hat{H}}_{\mathrm{HH}2}\cdot t}{⟶}{\hat{I}}_{z}\cdot {\mathrm{cos}}^{2}\left(\frac{\sqrt{D_{1}^{2}+D_{2}^{2}}}{2}t\right)\\ & -\frac{D_{1}\left({\hat{I}}_{x}{\hat{S}}_{1y}-{\hat{I}}_{y}{\hat{S}}_{1x}\right)+D_{2}\left({\hat{I}}_{x}{\hat{S}}_{2y}-{\hat{I}}_{y}{\hat{S}}_{2x}\right)}{\sqrt{D_{1}^{2}+D_{2}^{2}}}\;\mathrm{sin}\sqrt{D_{1}^{2}+D_{2}^{2}}t\\ & \;+\frac{D_{1}^{2}{\hat{S}}_{1z}+D_{2}^{2}{\hat{S}}_{2z}-4D_{1}D_{2}\;{\hat{I}}_{z}\left({\hat{S}}_{1x}{\hat{S}}_{2x}+{\hat{S}}_{1y}{\hat{S}}_{2y}\right)}{D_{1}^{2}+D_{2}^{2}}{\mathrm{sin}}^{2}\left(\frac{\sqrt{D_{1}^{2}+D_{2}^{2}}}{2}t\right) \end{array}$$
  Comments on the third item: the oscillation frequency is the geometric sum of the individual frequencies. The oscillation takes place between the initial state and a mixture of observable and unobservable states.

Note the first two propagation rules of the third group: in addition to the orthogonality relations (Eq. [Disp-formula Ch1.E25]), the linear sum of the prefactors of the first and third terms is 1. Both terms represent z magnetization. This condition reflects the fact that the sum of the z polarizations is constant for cross-polarization and polarization transfer. This is supported by the fact that ${\hat{I}}_{1z}+{\hat{I}}_{2z}$ commutes with ${\hat{H}}_{\mathrm{II}}$ and ${\hat{I}}_{z}+{\hat{S}}_{z}$ commutes with ${\hat{H}}_{\mathrm{HH}}$ . Figure [Fig Ch1.F2] shows the time evolution of the three prefactors in these propagation rules.

**Figure 2 Ch1.F2:**
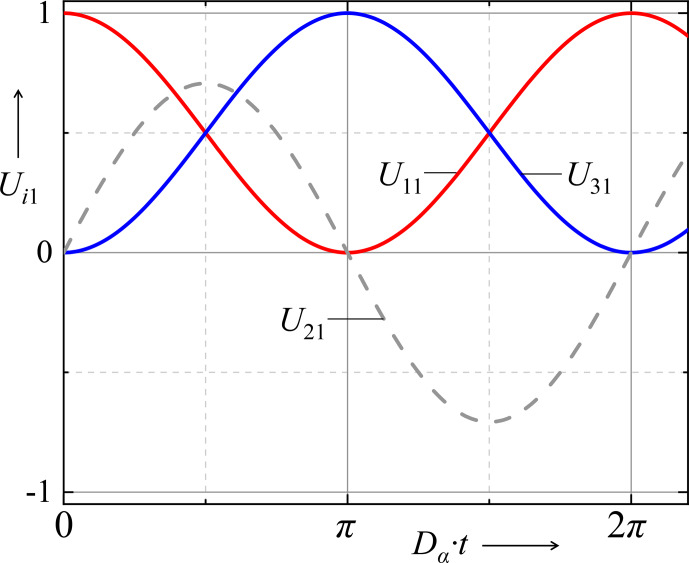
Time evolution of the three prefactors in the propagation rules shown in Eqs. (52) and (53).

#### 4.3.5 Group 4 of 3-D examples: cross-polarization, finite rf power, and possible deviation from the HH condition

- Considering the polarization difference in the rotating frame,
55 $$\begin{array}{rl} {\hat{S}}_{x} & \;-\;{\hat{I}}_{x}\overset{\left({\hat{H}}_{\mathrm{Ix}}+{\hat{H}}_{\mathrm{Sx}}+{\hat{H}}_{\mathrm{IS}}\right)\;t}{⟶}\left({\hat{S}}_{x}-{\hat{I}}_{x}\right)\cdot \frac{\omega_{\Delta }^{2}+D_{\mathrm{IS}}^{2}\mathrm{cos}q_{\Delta }t}{q_{\Delta }^{2}}\\ & \;-2\left({\hat{I}}_{z}{\hat{S}}_{y}-{\hat{I}}_{y}{\hat{S}}_{z}\right)\cdot \frac{D_{\mathrm{IS}}}{q_{\Delta }}\mathrm{sin}q_{\Delta }t\\ & \;+2\left({\hat{I}}_{z}{\hat{S}}_{z}+{\hat{I}}_{y}{\hat{S}}_{y}\right)\cdot \frac{\omega_{\Delta }D_{\mathrm{IS}}}{q_{\Delta }^{2}}{\mathrm{sin}}^{2}q_{\Delta }t, \end{array}$$
  with $\omega_{\Delta }$ = $\omega_{1S}-\omega_{1I}$ and $q_{\Delta }^{2}$ = $D_{\mathrm{IS}}^{2}+\omega_{\Delta }^{2}$ , there are the following comments:
  - The CP oscillation frequency is no longer $D_{\mathrm{IS}}$ as calculated for infinite rf power (see above), but $\sqrt{D_{\mathrm{IS}}^{2}+{\left(\omega_{1I}-\omega_{1S}\right)}^{2}}$ , which increases with the difference in the two rf field strengths.
  - Moreover, only the relative part $D_{\mathrm{IS}}^{2}/q_{\Delta }^{2}$ of the total magnetization participates in the oscillation. That is, the greater the deviation from the Hartmann–Hahn condition, the lower the maximum transmitted polarization.
- Consider the polarization *sum*:
56 $$\begin{array}{rl} {\hat{S}}_{x} & \;+\;{\hat{I}}_{x}\;\overset{\left({\hat{H}}_{\mathrm{Ix}}+{\hat{H}}_{\mathrm{Sx}}+{\hat{H}}_{\mathrm{IS}}\right)\;t}{⟶}\;\left({\hat{S}}_{x}+{\hat{I}}_{x}\right)\cdot \frac{4\omega_{ø}^{2}+D_{\mathrm{IS}}^{2}\mathrm{cos}q_{ø}t}{q_{ø}^{2}}\\ & \;-2\left({\hat{I}}_{z}{\hat{S}}_{y}+{\hat{I}}_{y}{\hat{S}}_{z}\right)\cdot \frac{D_{\mathrm{IS}}}{q_{ø}}\mathrm{sin}q_{ø}t\\ & +2\left({\hat{I}}_{z}{\hat{S}}_{z}-{\hat{I}}_{y}{\hat{S}}_{y}\right)\cdot \frac{2\omega_{ø}D_{\mathrm{IS}}}{q_{ø}^{2}}{\mathrm{sin}}^{2}q_{ø}t, \end{array}$$
  with $\omega_{ø}$ = $\left(\omega_{1S}+\omega_{1I}\right)/2$ and $q_{ø}^{2}$ = $D_{\mathrm{IS}}^{2}+4\omega_{ø}^{2}$ .

In contrast to the relations obtained for infinite $\omega_{1}$ , the sum of both polarizations oscillates. The amplitude decreases with increasing rf power. This phenomenon is analogous to what happens with spin locking and decoupling (see the corresponding examples above).

### 4.4 Case of reduction to a 4-D subspace

#### 4.4.1 Generic notation

This group of situations can be described by commutator relations of the forms

57 $$\begin{array}{rl} \left[\hat{H},\hat{A}\right] & =\;\;\;\;ia\hat{B},\\ \left[\hat{H},\hat{B}\right] & =-ia\hat{A}+ib\hat{C},\\ \left[\hat{H},\hat{C}\right] & =-ib\hat{B}\mp ia\hat{D},\\ \left[\hat{H},\hat{D}\right] & =\pm ia\hat{C}, \end{array}$$

where $\hat{A}$ , $\hat{B}$ , $\hat{C}$ , and $\hat{D}$ are pairwise orthogonal operators with equal norms. According to step 2, we obtain the Liouvillian matrix from these rules as a transposed coefficient matrix

58 $$L_{4D}=\left(\begin{array}{cccc} 0 & -ia & 0 & 0\\ ia & 0 & -ib & 0\\ 0 & ib & 0 & \pm ia\\ 0 & 0 & \mp ia & 0 \end{array}\right)$$

and the superpropagator matrix as

59 $$\begin{array}{rl} & U_{4D}=\frac{1}{2W}\\ & \left(\begin{array}{cccc} q_{2}\mathrm{cos}q_{1}t+q_{1}\mathrm{cos}q_{2}t & -a(\mathrm{sin}q_{1}t+\mathrm{sin}q_{2}t) & a(\mathrm{cos}q_{1}t-\mathrm{cos}q_{2}t) & \mp q_{2}\mathrm{sin}q_{1}t\pm q_{1}\mathrm{sin}q_{2}t\\ a(\mathrm{sin}q_{1}t+\mathrm{sin}q_{2}t) & q_{1}\mathrm{cos}q_{1}t+q_{2}\mathrm{cos}q_{2}t & q_{2}\mathrm{sin}q_{2}t-q_{1}\mathrm{sin}q_{1}t & \pm a(\mathrm{cos}q_{2}t-\mathrm{cos}q_{1}t)\\ a(\mathrm{cos}q_{2}t-\mathrm{cos}q_{1}t) & q_{1}\mathrm{sin}q_{1}t-q_{2}\mathrm{sin}q_{2}t & q_{1}\mathrm{cos}q_{1}t+q_{2}\mathrm{cos}q_{2}t & \mp a(\mathrm{sin}q_{1}t+\mathrm{sin}q_{2}t)\\ \pm q_{2}\mathrm{sin}q_{1}t\mp q_{1}\mathrm{sin}q_{2}t & \pm a(\mathrm{cos}q_{2}t-\mathrm{cos}q_{1}t) & \pm a(\mathrm{sin}q_{1}t+\mathrm{sin}q_{2}t) & q_{2}\mathrm{cos}q_{1}t+q_{1}\mathrm{cos}q_{2}t \end{array}\right), \end{array}$$

with $W^{2}\;:=(a^{2}+b^{2})/4$ and $q_{1;2}\;:=W\mp b/2$ . The propagation rule for the case where $\hat{A}$ was the initial state can be read from the first column of $U_{4D}$ in Eq. ([Disp-formula Ch1.E59]):

60 $$\begin{array}{rl} \hat{A}\;\overset{\hat{H}\cdot t}{⟶} & \;\;\hat{A}\cdot \frac{q_{2}\mathrm{cos}q_{1}t+q_{1}\mathrm{cos}q_{2}t}{2W}\\ & \;+\hat{B}\cdot \frac{a(\mathrm{sin}q_{1}t+\mathrm{sin}q_{2}t)}{2W}\\ & \;+\hat{C}\cdot \frac{a(\mathrm{cos}q_{2}t-\mathrm{cos}q_{1}t)}{2W}\\ & \;\pm \hat{D}\cdot \frac{q_{2}\mathrm{sin}q_{1}t-q_{1}\mathrm{sin}q_{2}t}{2W}. \end{array}$$

In some cases, another form of this propagation formula may be appropriate for use; this is obtained from Eq. ([Disp-formula Ch1.E60]) by applying some trigonometric rules:

61A^⟶H^⋅tA^⋅cos⁡(Wt)cos⁡bt2+b2Wsin⁡(Wt)sin⁡bt2+B^⋅aWsin⁡(Wt)cos⁡bt262-C^⋅aWsin⁡(Wt)sin⁡bt2∓D^⋅cos⁡(Wt)sin⁡bt2+b2Wsin⁡(Wt)cos⁡bt2.

#### 4.4.2 Examples

- *AB spin system* with J coupling and distance of the two lines Δν:=Δω/(2π) ; the spectrometer frequency is assumed to be set at the midpoint between the two resonances:
63 $$\begin{array}{rl} & {\hat{I}}_{1x}+{\hat{I}}_{2x}\;\;\overset{2\pi J{\hat{I}}_{1z}{\hat{I}}_{2z}\;t}{⟶}\\ & \;\;\left({\hat{I}}_{1x}+{\hat{I}}_{2x}\right)\cdot \frac{q_{2}\mathrm{cos}q_{1}t+q_{1}\mathrm{cos}q_{2}t}{W}\\ & \;\;+\left({\hat{I}}_{1y}-{\hat{I}}_{2y}\right)\cdot \frac{\Delta \omega }{2}\frac{\mathrm{sin}q_{1}t+\mathrm{sin}q_{2}t}{W}\\ & \;\;+2\left({\hat{I}}_{1z}{\hat{I}}_{2x}-{\hat{I}}_{1x}{\hat{I}}_{2z}\right)\cdot \frac{\Delta \omega }{2}\frac{\mathrm{cos}q_{1}t-\mathrm{cos}q_{2}t}{W}\\ & \;\;-2\left({\hat{I}}_{1y}{\hat{I}}_{2z}+{\hat{I}}_{1z}{\hat{I}}_{2y}\right)\cdot \frac{q_{1}\mathrm{sin}q_{2}t-q_{2}\mathrm{sin}q_{1}t}{W}, \end{array}$$
  where $W=2\pi \sqrt{J^{2}+\Delta \nu^{2}}$ and $q_{1;2}=\pi \left(\sqrt{J^{2}+\Delta \nu^{2}}\mp J\right)$ . The cosine terms contain two frequencies that give four line positions $\pm q_{1}$ and $\pm q_{2}$ symmetrically around zero after complex Fourier transformation. The intensities are given by the prefactors of the corresponding trigonometric functions. The lower-frequency oscillation has the larger prefactor, which reflects the roof effect. See, e.g., [Bibr bib1.bibx1], Chap. XI, Sect. B. In this book, positions and intensities are calculated from transition frequencies and probabilities for the transitions between the levels. Figure [Fig Ch1.F3] shows the relationship between $q_{1}$ , $q_{2}$ , and the intensities and positions of the four lines of an AB spin system.
  For the case Δν=0 , the Hamiltonian commutes with the initial state, with the consequence that the density operator remains constant. The Fourier transform of this is just a resonance at zero frequency.
- *rf irradiation onto homonuclearly coupled spins* 1/2 which are initially in equilibrium (nutation):
64 $$\begin{array}{rl} & {\hat{I}}_{1z}+{\hat{I}}_{2z}\;\;\overset{\left({\hat{H}}_{\mathrm{II}}+{\hat{H}}_{\mathrm{Ix}}\right)t}{⟶}\left({\hat{I}}_{1z}+{\hat{I}}_{2z}\right)\\ & \;\;\cdot \left(\mathrm{cos}Wt\mathrm{cos}\frac{3D_{\mathrm{II}}}{4}t+\frac{3D_{II}}{4W}\mathrm{sin}Wt\mathrm{sin}\frac{3D_{\mathrm{II}}}{4}t\right)\\ & \;+\left({\hat{I}}_{1y}+{\hat{I}}_{2y}\right)\cdot \frac{\omega_{1I}}{W}\mathrm{sin}Wt\;\mathrm{cos}\frac{3D_{\mathrm{II}}}{4}t\\ & \;-2\left({\hat{I}}_{1x}{\hat{I}}_{2z}+{\hat{I}}_{1z}{\hat{I}}_{2x}\right)\cdot \frac{\omega_{1I}}{W}\mathrm{sin}Wt\;\mathrm{cos}\frac{3D_{\mathrm{II}}}{4}t\\ & \;-2\left({\hat{I}}_{1y}{\hat{I}}_{2x}+{\hat{I}}_{1x}{\hat{I}}_{2y}\right)\cdot \left(\mathrm{cos}Wt\mathrm{sin}\frac{3D_{\mathrm{II}}}{4}t-\frac{3D_{\mathrm{II}}}{4W}\mathrm{sin}Wt\mathrm{cos}\frac{3D_{\mathrm{II}}}{4}t\right), \end{array}$$
  where W = $\sqrt{\omega_{1I}^{2}+\frac{9}{16}D_{\mathrm{II}}^{2}}$ .
  This propagation formula describes the effect of a limited-power rf pulse on the equilibrium magnetization. The time evolution of the prefactor of ${\hat{I}}_{1y}+{\hat{I}}_{2y}$ is shown in Fig. [Fig Ch1.F4].
  Comments on Eq. ([Disp-formula Ch1.E64]):
  - As the coupling frequency increases, so does the nutation frequency W . However, this oscillation is modulated by half of the dipolar frequency (Fig. [Fig Ch1.F4]).
  - As a consequence, the π and π/2 conditions for achieving maximum and zero y magnetization are modified with respect to the coupling-free case. Similar to Eq. ([Disp-formula Ch1.E48]), this results in
65 $$\begin{array}{rl} & \tau_{\pi /2}=\frac{\pi }{2\sqrt{\omega_{1I}^{2}+\frac{9}{16}D_{\mathrm{II}}^{2}}},\\ & \tau_{\pi }=\frac{\pi }{\sqrt{\omega_{1I}^{2}+\frac{9}{16}D_{\mathrm{II}}^{2}}}. \end{array}$$
- *Nutation under quadrupolar interaction*, spin 1:
66 $$\begin{array}{rl} & {\hat{I}}_{z}\;\;\overset{\left({\hat{H}}_{Q}+{\hat{H}}_{\mathrm{Ix}}\right)t}{⟶}\\ & \;\;{\hat{I}}_{z}\cdot \left(\mathrm{cos}\frac{Wt}{2}\mathrm{cos}\frac{\omega_{Q}t}{2}+\frac{\omega_{Q}t}{W}\mathrm{sin}\frac{Wt}{2}\mathrm{sin}\frac{\omega_{Q}t}{2}\right)\\ & \;+{\hat{I}}_{y}\cdot \frac{2\omega_{1}}{W}\mathrm{sin}\frac{Wt}{2}\mathrm{sin}\frac{\omega_{Q}t}{2}-\left({\hat{I}}_{x}{\hat{I}}_{z}+{\hat{I}}_{z}{\hat{I}}_{x}\right)\\ & \;\cdot \frac{2\omega_{1}}{W}\mathrm{sin}\frac{Wt}{2}\mathrm{sin}\frac{\omega_{Q}t}{2}+\left({\hat{I}}_{y}{\hat{I}}_{x}+{\hat{I}}_{x}{\hat{I}}_{y}\right)\\ & \;\cdot \left(\mathrm{cos}\frac{Wt}{2}\mathrm{sin}\frac{\omega_{Q}t}{2}+\frac{\omega_{Q}t}{W}\mathrm{sin}\frac{Wt}{2}\mathrm{cos}\frac{\omega_{Q}t}{2}\right), \end{array}$$
  with W = $\sqrt{4\omega_{1}^{2}+\omega_{Q}^{2}}$ . This is consistent with the findings of [Bibr bib1.bibx4], [Bibr bib1.bibx3], and [Bibr bib1.bibx23]. Again, the result is that nutation occurs more quickly than $\omega_{1I}$ if there is an additional interaction.

**Figure 3 Ch1.F3:**
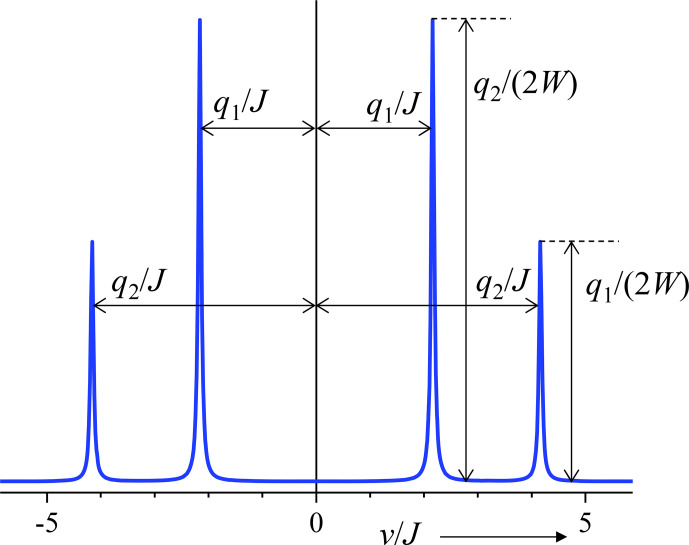
Connection between the two oscillation frequencies, the positions, and the intensities of lines in the spectrum of an AB spin system (example: Δν=3J ).

**Figure 4 Ch1.F4:**
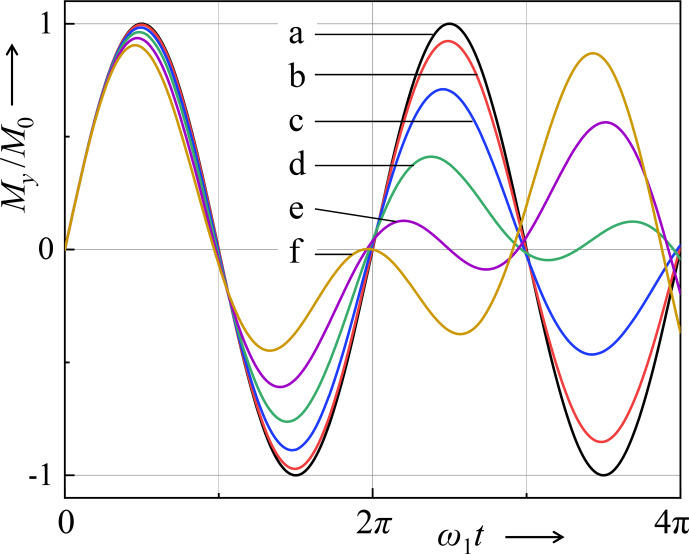
Nutation curves for different ratios of rf field strength and coupling strength. For homonuclear dipolar coupling, $D_{\mathrm{II}}/\omega_{1}$ = **(a)** 0, **(b)** 0.15, **(c)** 0.3, **(d)** 0.45, **(e)** 0.6, and **(f)** 0.75. For quadrupolar coupling, $\omega_{Q}/\omega_{1}$ = **(a)** 0, **(b)** 0.2, **(c)** 0.4, **(d)** 0.6, **(e)** 0.8, and **(f)** 1.

### 4.5 Example of a 5-D subspace

We consider cross-polarization under a finite rf power, assuming that the Hartmann–Hahn condition is satisfied: $\omega_{1I}=\omega_{1S}=\;:\omega_{1}$ . Other than in the corresponding 3-D examples shown above, the initial state consists of a transversally polarized S spin and a depolarized I spin, i.e., $\hat{\rho }(0)={\hat{S}}_{x}$ :

67 $$\begin{array}{rl} {\hat{S}}_{x} & \;\overset{({\hat{H}}_{\mathrm{Ix}}+{\hat{H}}_{\mathrm{Sx}}+{\hat{H}}_{\mathrm{IS}})\cdot t}{⟶}\;\frac{1}{2}[\left(\mathrm{cos}D_{\mathrm{IS}}t+\frac{4\omega_{1}^{2}+D_{\mathrm{IS}}^{2}\mathrm{cos}qt}{q^{2}}\right)\cdot {\hat{S}}_{x}\\ & \;-\left(\frac{D_{\mathrm{IS}}}{q}\mathrm{sin}qt+\mathrm{sin}D_{\mathrm{IS}}t\right)\cdot 2{\hat{I}}_{z}{\hat{S}}_{y}\\ & \;+4\frac{D_{\mathrm{IS}}\omega_{1}}{q^{2}}(1-\mathrm{cos}qt)\left({\hat{I}}_{z}{\hat{S}}_{z}-{\hat{I}}_{y}{\hat{S}}_{y}\right)\\ & \;+\left(\frac{D_{\mathrm{IS}}}{q}\mathrm{sin}qt-\mathrm{sin}D_{\mathrm{IS}}t\right)\cdot 2{\hat{I}}_{y}{\hat{S}}_{z}\\ & \;+\left(-\mathrm{cos}D_{\mathrm{IS}}t+\frac{4\omega_{1}^{2}+D_{\mathrm{IS}}^{2}\mathrm{cos}qt}{q^{2}}\right)\cdot {\hat{I}}_{x}]. \end{array}$$

Equation ([Disp-formula Ch1.E67]) describes the evolution of the magnetization of the initially polarized spin. That of the other spin evolves as given in Eq. ([Disp-formula Ch1.E68]):

68 $$\begin{array}{rl} {\hat{I}}_{x} & \;\overset{({\hat{H}}_{\mathrm{Ix}}+{\hat{H}}_{\mathrm{Sx}}+{\hat{H}}_{\mathrm{IS}})\cdot t}{⟶}\;\frac{1}{2}[{\hat{S}}_{x}\cdot \left(-\mathrm{cos}D_{\mathrm{IS}}t+\frac{4\omega_{1}^{2}+D_{\mathrm{IS}}^{2}\mathrm{cos}qt}{q^{2}}\right)\\ & \;-\left(\frac{D_{\mathrm{IS}}}{q}\mathrm{sin}qt-\mathrm{sin}D_{\mathrm{IS}}t\right)\cdot 2{\hat{I}}_{z}{\hat{S}}_{y}\\ & \;+4\frac{D_{\mathrm{IS}}\omega_{1}}{q^{2}}(1-\mathrm{cos}qt)\left({\hat{I}}_{z}{\hat{S}}_{z}-{\hat{I}}_{y}{\hat{S}}_{y}\right)\\ & \;-\left(\frac{D_{\mathrm{IS}}}{q}\mathrm{sin}qt+\mathrm{sin}D_{\mathrm{IS}}t\right)\cdot 2{\hat{I}}_{y}{\hat{S}}_{z}\\ & \;+\left(\mathrm{cos}D_{\mathrm{IS}}t+\frac{4\omega_{1}^{2}+D_{\mathrm{IS}}^{2}\mathrm{cos}qt}{q^{2}}\right)\cdot {\hat{I}}_{x}]. \end{array}$$

### 4.6 Examples of a 6-D subspace

- In the Hartmann–Hahn cross-polarization experiment with finite rf power and deviation from the Hartmann–Hahn condition, the initial state consists of a transversally polarized S spin and a depolarized I spin, i.e., $\hat{\rho }(0)={\hat{S}}_{x}$ . Using the variables defined in Sect. [Sec Ch1.S4.SS3.SSS5], we obtain
69 $$\begin{array}{rl} & {\hat{S}}_{x}\;\;\overset{({\hat{H}}_{\mathrm{Ix}}+{\hat{H}}_{\mathrm{Sx}}+{\hat{H}}_{\mathrm{IS}})\cdot t}{⟶}\\ & {\hat{S}}_{x}\cdot \frac{1}{2}\left(\frac{\omega_{\Delta }^{2}+D_{\mathrm{IS}}^{2}\mathrm{cos}q_{\Delta }t}{q_{\Delta }^{2}}+\frac{4\omega_{ø}^{2}+D_{\mathrm{IS}}^{2}\mathrm{cos}q_{ø}t}{q_{ø}^{2}}\right)\\ & -{\hat{I}}_{z}{\hat{S}}_{y}\cdot D_{\mathrm{IS}}\left(\frac{\mathrm{sin}q_{\Delta }t}{q_{\Delta }}+\frac{\mathrm{sin}q_{ø}t}{q_{ø}}\right)\\ & +2{\hat{I}}_{z}{\hat{S}}_{z}D_{\mathrm{IS}}\left[\frac{\omega_{\Delta }}{2q_{\Delta }}(1-\mathrm{cos}q_{\Delta }t)+\frac{\omega_{ø}}{q_{ø}}(1-\mathrm{cos}q_{ø}t)\right]\\ & +2{\hat{I}}_{y}{\hat{S}}_{y}D_{\mathrm{IS}}\left[\frac{\omega_{\Delta }}{2q_{\Delta }}(1-\mathrm{cos}q_{\Delta }t)-\frac{\omega_{ø}}{q_{ø}}(1-\mathrm{cos}q_{ø}t)\right]\\ & +{\hat{I}}_{y}{\hat{S}}_{z}\cdot D_{\mathrm{IS}}\left(\frac{\mathrm{sin}q_{\Delta }t}{q_{\Delta }}-\frac{\mathrm{sin}q_{ø}t}{q_{ø}}\right)\\ & +{\hat{I}}_{x}\cdot \frac{1}{2}\left(\frac{4\omega_{ø}^{2}+D_{\mathrm{IS}}^{2}\mathrm{cos}q_{ø}t}{q_{ø}^{2}}-\frac{\omega_{\Delta }^{2}+D_{\mathrm{IS}}^{2}\mathrm{cos}q_{\Delta }t}{q_{\Delta }^{2}}\right). \end{array}$$
  Figure [Fig Ch1.F5] shows the buildup curve for the I magnetization for the case where it was initially unpolarized and S was polarized. For zero deviation from the Hartmann–Hahn condition, the curve still looks similar to the squared sine derived for infinite rf power (Eq. [Disp-formula Ch1.E53]). Small deformations result from the fact that the rf power is finite. An increasing deviation from the Hartmann–Hahn condition leads to a strong loss of the polarization-transfer efficiency, in addition to further deviation from the ideal curve.
- *Hartmann–Hahn cross-polarization of an I spin from two S spins.* The Hamiltonian is the same as in Sect. [Sec Ch1.S4.SS3.SSS4], and we consider the problem in the doubly rotating frame. In this example, however, we want to follow the time evolution of all three spins individually which could not be separated in the 3-D example. The propagation rules for all three spins can be found in the Supplement. Here is the propagation rule for the case where the system starts with polarized S spins, i.e., $\hat{A}={\hat{S}}_{1z}+{\hat{S}}_{2z}$ and $\hat{H}={\hat{H}}_{\mathrm{HH}2}$ :
70 $$\begin{array}{rl} & {\hat{S}}_{1z}+{\hat{S}}_{2z}\;\;\overset{{\hat{H}}_{\mathrm{HH}2}\cdot t}{⟶}\;\;{\hat{I}}_{z}\cdot {\mathrm{sin}}^{2}\frac{qt}{2}\\ & \;\;+\left({\hat{S}}_{1z}+{\hat{S}}_{2z}\right)\cdot \frac{1+{\mathrm{cos}}^{2}\frac{qt}{2}}{2}\\ & \;\;+\left({\hat{S}}_{1z}-{\hat{S}}_{2z}\right)\cdot \frac{D_{2}^{2}-D_{1}^{2}}{2q^{2}}{\mathrm{sin}}^{2}\frac{qt}{2}\\ & \;\;+\left({\hat{I}}_{x}{\hat{S}}_{1y}-{\hat{I}}_{y}{\hat{S}}_{1x}\right)\frac{D_{1}}{q}\mathrm{sin}qt\\ & \;\;+\left({\hat{I}}_{x}{\hat{S}}_{2y}-{\hat{I}}_{y}{\hat{S}}_{2x}\right)\frac{D_{2}}{q}\mathrm{sin}qt\\ & \;\;-{\hat{I}}_{z}\left({\hat{S}}_{1x}{\hat{S}}_{2x}-{\hat{S}}_{1y}{\hat{S}}_{2y}\right)\cdot 4\frac{D_{1}D_{2}}{q^{2}}{\mathrm{sin}}^{2}\frac{qt}{2}, \end{array}$$
  with q = $\sqrt{D_{1}^{2}+D_{2}^{2}}$ . This propagation rule describes the oscillatory polarization exchange between the spin polarizations and three unobservable states on the one hand and the oscillatory polarization transfer between the spins on the other hand, as may happen, for example, in ^13^CH_2_ or ^15^NH_2_ groups.

**Figure 5 Ch1.F5:**
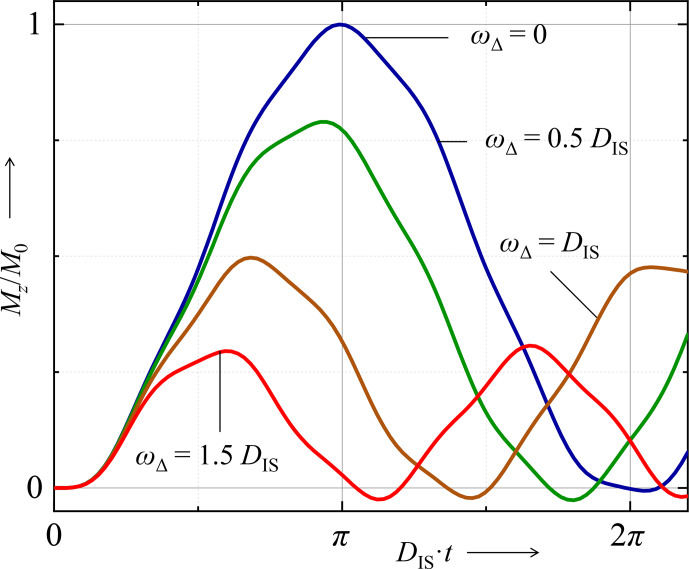
Examples of cross-polarization buildup curves for the I spins in an ensemble of spin pairs I , S corresponding to Eq. ([Disp-formula Ch1.E69]) with $\omega_{ø}=3D_{\mathrm{IS}}$ . The deviation $\omega_{\Delta }$ from the Hartmann–Hahn condition is varied.

## 5 Outlook: time-dependent Hamiltonians and Liouvillians

In order to obtain the propagator matrices and the propagation formulae of the respective Liouvillians, it was assumed in the above sections that the interactions and thus also the Hamiltonian and Liouvillian are time-invariant. However, the method can be extended to situations where the interaction constants vary with time, fluctuating due to thermal motion or periodically due to sample spinning. The commutator equations are also valid; i.e., steps (1) and (2) of the given procedure can be performed to obtain the relevant subspace and the Liouvillian matrix associated with that subspace. In other words, the Liouvillian matrices obtained for the above examples (see the Supplement) can also be used in the time-dependent situations. The Liouville–von Neumann equation now belongs to a system of linear differential equations but with time-dependent coefficients. There is no general scheme for their integration. The propagator matrix is no longer the matrix exponential of -iLt .

However, in some cases solutions are possible in the following ways: (i) use of the time-averaged Liouvillian for an exact solution when L depends linearly on a single time-dependent parameter and (ii) use of the Shirley method, based on the Floquet theorem, also for an analytical solution.

The first way can be justified as follows: for the Liouville–von Neumann equation in matrix form, one can try to find an effective Liouvillian matrix $L_{\mathrm{eff}}(t)$ , which is defined as that matrix which is constant in the interval (0,t) and which has the same effect as the actual L , i.e., $U(t)=\mathrm{exp}(-iL_{\mathrm{eff}}t)$ in matrix notation. This can be done using the Magnus expansion [Bibr bib1.bibx15]:

71 $$L_{\mathrm{eff}}=\int_{0}^{t}L(t_{1})\;dt_{1}+\frac{1}{2}\int_{0}^{t}\left[L(t_{1}),L(t_{2})\right]dt_{1}dt_{2}+O(L^{3}).$$

Although the convergence radius of this series is rather small [Bibr bib1.bibx16], it can nevertheless be used to check whether the effective Liouvillian can be replaced with the time-averaged Liouvillian (first term on the right-hand side of Eq. [Disp-formula Ch1.E71]). If the Liouvillian commutes at all times with itself, all higher-order terms vanish and only the zeroth-order term survives. In this case, the effective Liouvillian is equal to the time-averaged Liouvillian. This happens in addition to the case of a constant Liouvillian (see above) if the Liouvillian matrix can be written as a product of a scalar function λ(t) with a constant matrix:

72 $$L(t)=\lambda (t)\;A\;\;\;\to \;\;\;L_{\mathrm{eff}}=A\int_{0}^{t}\lambda (t_{1})\;dt_{1}.$$

In this case, the propagator matrix is exactly the matrix exponential

73 $$U(t)=\mathrm{exp}\left[-iA\int_{0}^{t}\lambda (t_{1})\;dt_{1}\right].$$

This concerns all 2-D cases. Equation ([Disp-formula Ch1.E31]) becomes

74 $$\begin{array}{l} L_{2D}=\lambda (t)\left(\begin{array}{cc} 0 & -i\\ i & 0 \end{array}\right),\\ \begin{array}{rl} U_{2D} & =\mathrm{exp}\left(-i\int_{0}^{t}L_{2D}(t_{1})\;dt_{1}\right)\\ & \;=\left(\begin{array}{cc} \mathrm{cos}\int_{0}^{t}\lambda (t_{1})\;dt_{1} & -\mathrm{sin}\int_{0}^{t}\lambda (t_{1})\;dt_{1}\\ \mathrm{sin}\int_{0}^{t}\lambda (t_{1})\;dt_{1} & \mathrm{cos}\int_{0}^{t}\lambda (t_{1})\;dt_{1} \end{array}\right). \end{array} \end{array}$$

The transformation to propagation formulae gives

75 $$\begin{array}{l} \hat{A}\;\overset{\hat{H}\;t}{⟶}\hat{A}\mathrm{cos}\int_{0}^{t}\lambda (t_{1})\;dt_{1}+\hat{B}\mathrm{sin}\int_{0}^{t}\lambda (t_{1})\;dt_{1},\\ \hat{B}\;\overset{\hat{H}\;t}{⟶}\hat{B}\mathrm{cos}\int_{0}^{t}\lambda (t_{1})\;dt_{1}-\hat{A}\mathrm{sin}\int_{0}^{t}\lambda (t_{1})\;dt_{1}. \end{array}$$

Equation ([Disp-formula Ch1.E75]) can be regarded as a rigorous extension of the POF to time-dependent systems, such as those caused by thermal motion or sample spinning. For higher-dimensional cases, Eq. ([Disp-formula Ch1.E72]) can only be satisfied if the two parameters a and b are equal. While for the 4-D, 5-D, and 6-D cases this would mean very special situations, the 3-D case includes with a=b the important case of cross-polarization. Then Eq. ([Disp-formula Ch1.E38]) changes to

76 $$L_{3D}=a(t)\left(\begin{array}{ccc} 0 & -i & 0\\ i & 0 & -i\\ 0 & i & 0 \end{array}\right)$$

if b is replaced with a . In the subsequent propagation formulae, we have to substitute qt with $\int_{0}^{t}q(t_{1})\;dt_{1}$ , with $q(t)=a(t)\sqrt{2}$ . This means that we obtain an exact solution if we replace the arguments of the trigonometric functions with integrals in the following examples from above:

- For Eqs. ([Disp-formula Ch1.E32]), ([Disp-formula Ch1.E35]), and ([Disp-formula Ch1.E52]), replace $D_{\mathrm{II}}t$ with $\int_{0}^{t}D_{\mathrm{II}}(t_{1})\;dt_{1}$ .
- For Eqs. ([Disp-formula Ch1.E33]), ([Disp-formula Ch1.E36]), and ([Disp-formula Ch1.E53]), replace $D_{\mathrm{IS}}t$ with $\int_{0}^{t}D_{\mathrm{IS}}(t_{1})\;dt_{1}$ .
- For Eq. ([Disp-formula Ch1.E34]), replace $\omega_{Q}t$ with $\int_{0}^{t}\omega_{Q}(t_{1})\;dt_{1}$ .

However, there is no general recipe for all the other cases. In some of the cases, the Shirley method using the Floquet theorem, which is applied, e.g., for numerical calculations of spin systems under MAS, may also be successful in obtaining analytical expressions. This will be the subject of a forthcoming paper.

## 6 Conclusions

Repeated application of the commutator of the Hamiltonian with the initial density operator gives a system of operator equations, the coefficient matrix of which can be used to establish a propagation rule for the spin system. This has been demonstrated in this paper with some examples. A more detailed analysis shows that the commutator relations define subspaces which are both Liouvillian-invariant and superpropagator-invariant. Therefore, the density operator propagates in such a subspace without leaving it. If its dimension is small enough, analytical expressions for the propagation law can be obtained.

The relevant subspace for a given problem is determined by the Hamiltonian and by the initial state. If the operator characterizing the initial state changes, then the new subspace is the same as the previous one if and only if the new initial state operator is an element of the previous subspace. Otherwise, the two subspaces have no intersection.

The set of problems can be divided into classes with respect to the dimension of the subspaces. Problems of the 2-D class can be treated easily by propagation formulae similar to those of the well-known product–operator formalism. The propagation formulae for the 3-D and 4-D classes are given in this paper, and an example is given for the 5-D and 6-D cases. If necessary, the method introduced and explained here can also be applied to cases with higher dimensions. The application examples demonstrate the same mathematical structure as some physically different problems.

In addition, this treatment can be applied to pulse sequences in a manner similar to the POF. In some cases, an algebraic language program may be helpful. Even numerical computations can use this framework by starting the numerical computations on the basis of existing analytical relations. In the N -dimensional wave-function space, the Liouville–von Neumann equation corresponds to a system of $N^{2}$ differential equations, which is significantly larger than that obtained by the dimension reduction with the method presented here. It may be advantageous to first apply this method on an analytical basis before starting the numerical implementation.

If the strength of any of the considered interactions depends on time, the first two steps of this method can be applied likewise. The third step (matrix exponentialization), however, has to be modified because there is no general recipe for solving a system of differential equations with time-dependent coefficients. In some cases, the time-averaged Liouvillian is suitable for getting an exact solution to the problem. Even for numerical calculations it can be helpful to start with systems of differential equations containing a reduced number of equations.

## Supplement

10.5194/mr-6-77-2025-supplement The supplement related to this article is available online at https://doi.org/10.5194/mr-6-77-2025-supplement.

## Supplement

10.5194/mr-6-77-2025-supplement
10.5194/mr-6-77-2025-supplement The supplement related to this article is available online at https://doi.org/10.5194/mr-6-77-2025-supplement.

## Data Availability

No data sets were used in this article.

## Appendix A Norms of spin operators

Consider a system of N spins 1/2 . As proven in the Supplement (Sect. S1.3), the norm of a product of the Cartesian components of n spins from this N -spin system (one operator for each spin) amounts to $2^{(N/2)-n}$ . Furthermore, for the sum of two orthogonal operators $\hat{A}$ and $\hat{B}$ , $∥\hat{A}+\hat{B}∥=\sqrt{∥\hat{A}{∥}^{2}+∥\hat{B}{∥}^{2}}$ . From this, we can derive the following rules ( α , β , γ , and δ∈{x,y,z} ):

- For single spins $I=\frac{1}{2}$ : $∥{\hat{I}}_{\alpha }∥=\frac{1}{\sqrt{2}}$ .
- For spin pairs $I=\frac{1}{2}$ , $S=\frac{1}{2}$ : $∥{\hat{I}}_{\alpha }∥=∥{\hat{S}}_{\alpha }∥=1$ , $∥{\hat{I}}_{\alpha }{\hat{S}}_{\beta }∥=\frac{1}{2},∥{\hat{I}}_{\alpha }{\hat{S}}_{\beta }\pm {\hat{I}}_{\gamma }{\hat{S}}_{\delta }∥=\frac{1}{\sqrt{2}}$ .
- For spin triples $I=\frac{1}{2}$ , $J=\frac{1}{2}$ , $S=\frac{1}{2}$ : $∥{\hat{I}}_{\alpha }∥=∥{\hat{J}}_{\alpha }∥=∥{\hat{S}}_{\alpha }∥=\sqrt{2}$ , $∥{\hat{I}}_{\alpha }{\hat{S}}_{\beta }∥=\frac{1}{\sqrt{2}}$ , $∥{\hat{I}}_{\alpha }{\hat{S}}_{\beta }\pm {\hat{I}}_{\gamma }{\hat{S}}_{\delta }∥=1$ , $∥{\hat{I}}_{\alpha }{\hat{J}}_{\beta }{\hat{S}}_{\gamma }∥=\frac{1}{2\sqrt{2}}$ .

Similarly, for a single spin, I=1 can be obtained:

$$∥{\hat{I}}_{\alpha }∥=\sqrt{2},\;\;∥{\hat{I}}_{\alpha }{\hat{I}}_{\beta }+{\hat{I}}_{\beta }{\hat{I}}_{\alpha }∥=\sqrt{2}.$$

## Appendix B Hamiltonians used in the main part and in the Supplement

- Interaction of the I and S spins with the rf field, i.e., strengths $\omega_{1I}$ and $\omega_{1S}$ , respectively, assumed to be constant and parallel to the x axis in the rotating frame:
  B1
  $${\hat{H}}_{Ix}=-\omega_{1I}{\hat{I}}_{x};\;\;{\hat{H}}_{Sx}=-\omega_{1S}{\hat{S}}_{x}.$$
  Unless indicated otherwise, the irradiation occurs at the Larmor frequencies of the respective spins. The rf phase is always such that $B_{1}$ is parallel to the x axis of the rotating frame, but the calculations can easily be modified to include other directions of the rf field.
- Homonuclear dipolar interaction between spin $I_{1}$ and spin $I_{2}$ (secular part):
  B2
  $${\hat{H}}_{\mathrm{II}}=D_{\mathrm{II}}\left({\hat{I}}_{1}{\hat{I}}_{2}-3{\hat{I}}_{1z}{\hat{I}}_{2z}\right),$$
  where $D_{\mathrm{II}}=\left(\mu_{0}\gamma_{I}^{2}\hbar /\left(4\pi r_{\mathrm{II}}^{3}\right)\right)\left(3{\mathrm{cos}}^{2}\theta_{\mathrm{II}}-1\right)$ , $r_{\mathrm{II}}$ is the length of the vector connecting both spins, $\theta_{\mathrm{II}}$ is the angle of this vector with the external magnetic field, $\gamma_{I}$ is the gyromagnetic ratio of the I spins, and $\mu_{0}$ is the permeability of the vacuum.
- Heteronuclear dipolar interaction between spin I and spin S (secular part):
  B3
  $${\hat{H}}_{\mathrm{IS}}=-D_{\mathrm{IS}}\;\cdot \;2{\hat{I}}_{z}{\hat{S}}_{z},$$
  where $D_{\mathrm{IS}}=\frac{\mu_{0}}{4\pi }\frac{\hbar \gamma_{I}\gamma_{S}}{r_{\mathrm{IS}}^{3}}\left(3{\mathrm{cos}}^{2}\theta_{\mathrm{IS}}-1\right)$ , $r_{\mathrm{IS}}$ is the length of the vector connecting both spins, $\theta_{\mathrm{IS}}$ is the angle of this vector with the external magnetic field, and $\gamma_{S}$ is the gyromagnetic ratio of the S spins. The case of J coupling between nonequal spins is mathematically equivalent to this; the corresponding formulae can be obtained by replacing $D_{\mathrm{IS}}$ with -πJ .
- Matched Hartmann–Hahn cross-polarization between spin I and spin S in the doubly rotating frame [Bibr bib1.bibx7],
  B4
  $${\hat{H}}_{\mathrm{HH}}=-D_{\mathrm{IS}}\;\cdot \;\left({\hat{I}}_{x}{\hat{S}}_{x}+{\hat{I}}_{y}{\hat{S}}_{y}\right),$$
  assuming $D_{\mathrm{IS}}\ll \omega_{1I}$ and $\omega_{1S}$ so that rapidly oscillating terms can be neglected.
- First-order interaction of the nuclear quadrupole moment with the electric field gradient (secular part):
  B5
  $${\hat{H}}_{Q}=\omega_{Q}\;\cdot \;\left({\hat{I}}_{z}^{2}-\frac{I(I+1)}{3}\right),$$
  where $\omega_{Q}$ is the quadrupolar frequency.
- Resonance offset Δω in the rotating frame:
  B6
  $${\hat{H}}_{\Delta }=-\Delta \omega \;{\hat{I}}_{z}.$$

The coupling frequencies are assumed to be unique, i.e., with no powder-averaging unless otherwise stated.

## Appendix

The supplement related to this article is available online at https://doi.org/10.5194/mr-6-77-2025-supplement.
