# Supplementary material for: Analytical expressions for the time evolution of spin systems affected by two or more interactions
Source: Magn Reson (Gott). 2025 Feb 27;6(1):77–92. doi: 10.5194/mr-6-77-2025 (PMC12247082; doi:10.5194/mr-6-77-2025)
Supplement: The supplement related to this article is available online at https://doi.org/10.5194/mr-6-77-2025-supplement. [file mr-6-77-2025-supplement.pdf]

Supplement of Magn. Reson., 6, 77–92, 2025
https://doi.org/10.5194/mr-6-77-2025-supplement
© Author(s) 2025. CC BY 4.0 License.

Supplement of

Analytical expressions for the time evolution of spin systems affected by two or more interactions

Günter Hempel

Correspondence to: Günter Hempel (guenter.hempel@physik.uni-halle.de)

The copyright of individual parts of the supplement might differ from the article licence.

Contents

S1 PROOFS OF PROPOSITIONS OCCURRING IN THE MAIN PART 2

S1.1 Transposition relations between different matrix representations 2
S1.1.1 Generic superoperator acting on the density operator 2
S1.1.2 Consequences for the relations between commutator equations, Liouvillian matrix, and propagator matrix and propagation formulae 3

S1.2 Orthogonality of Hermitian operators to its commutators 3

S1.3 Rules for trace calculations 4

S2 MATRIX EXPONENTIALS 4

S3 APPLICATION EXAMPLES 5

S3.1 "Zero-dimensional" case 5
S3.1.1 Example 0D-1: Total *z* magnetization under homonuclear dipolar interaction 5
S3.1.2 Example 0D-2: Cross polarization, sum of *I* and *S* polarization (large rf powers) 5

S3.2 Examples for the two-dimensional case 6
S3.2.1 Procedure as explained in the main part 6
S3.2.2 First group of examples: FID and spin interactions. 6
S3.2.3 Second group: Polarization exchange 7

S3.3 Examples for the three-dimensional case 8
S3.3.1 Procedure as explained in the main part 8
S3.3.2 First group: rf irradiation at the observe channel, initially equilibrium, magnetization along B_0. 9
S3.3.3 Second group: rf irradiation during FID influenced by another interaction, initial state: transversal magnetization 11
S3.3.4 Third group: Polarization transfer; the relevant field strengths are much larger than the coupling frequency 13

S3.4 Examples for the four-dimensional case 18
S3.4.1 Procedure as explained in the main part 18

S3.5 Example of a five-dimensional case 21

S3.6 Examples of a six-dimensional case 23

S3.7 If possible: Decomposition of the Hamiltonian 26

S4 REFERENCES IN THE SI 26

S1 Proofs of propositions occurring in the main part

S1.1 Transposition relations between different matrix representations

The propositions proved in this subsection are needed (i) for the transition from the systems of commutator equations to the Liouvillian matrices by transposition of the coefficient matrices (for example, Main Part: Eq. (19) to (22) as general form, Eqs. (33) to (34) and (53) to (54) for the 3D and 4D cases, respectively), and (ii) for obtaining the propagation formulae from the propagator matrix.

S1.1.1 Generic superoperator acting on the density operator

Proposition 1: Let $\mathbb{S}$ be an operator space of dimension N, $\hat{\rho} \in \mathbb{S}$ a density operator and $\hat{\hat{A}}$ a superoperator acting on $\mathbb{S}$. The corresponding matrices are denoted by $\boldsymbol{\rho}$ and $\mathbf{A}$, respectively. Furthermore, let $\mathbf{u}$ be a column vector whose elements $\hat{u}_i$ $(i \in \{1..N\})$ are the operators of a basis of $\mathbb{S}$. Then the action of the superoperator on the density operator can be represented in two equivalent ways: (i) multiplication $\mathbf{A}\cdot\boldsymbol{\rho}$, (ii) multiplication $\mathbf{A}^{\mathrm{T}}\cdot\mathbf{u}$.

Proof:

The expansion of the density operator into the operator basis $\{\hat{u}_i\}$ is described by Eq. (8) of the Main Part as

$$\hat{\rho} = \mathbf{u}^{\mathrm{T}} \cdot \boldsymbol{\rho} \tag{S1}$$

($\mathbf{m}^{\mathrm{T}}$ means the transposition of the matrix $\mathbf{m}$.) It states that the density *operator* can be written as a generalized scalar product between two column matrices. The first one contains the basis operators, the second one the expansion coefficients.

The action of a superoperator $\hat{\hat{A}}$ on the density operator can be mapped to (symbol: $\mapsto$) the matrix space as

$$\hat{\hat{A}}\hat{\rho} \quad \mapsto \quad \mathbf{u}^{\mathrm{T}} \cdot (\mathbf{A} \cdot \boldsymbol{\rho}) \tag{S2}$$

Eq. (S2) corresponds to the multiplication of the superoperator matrix with the density column matrix. Applying usual matrix calculus, we get

$$\mathbf{u}^{\mathrm{T}} \cdot (\mathbf{A} \cdot \boldsymbol{\rho}) = \left(\mathbf{A}^{\mathrm{T}} \cdot \mathbf{u}\right)^{\mathrm{T}} \cdot \boldsymbol{\rho} \tag{S3}$$

The right-hand side can be interpreted as multiplication of the transpose of the superoperator matrix with the column containing the basis operators.

Eq. (S3) completes the proof of proposition 1.

■

Remark: The product $\mathbf{A}\cdot\boldsymbol{\rho}$ belongs to the mapping of the operator space to the matrix space with a basis {(1,0,0,…,0), (0,1,0,…,0),…}. In contrast, $\mathbf{A}^{\mathrm{T}}\cdot\mathbf{u}$ belongs to the reversed mapping of the matrix space to the operator space with the basis $\{\hat{u}_1,...,\hat{u}_N\}$. That means, the space containing $\mathbf{A}$ is the dual space of the space containing $\mathbf{A}^{\mathrm{T}}$, and the transposition represents the well-known relation between corresponding matrices from both spaces.

S1.1.2 Consequences for the relations between commutator equations, Liouvillian matrix, and propagator matrix and propagation formulae

Proposition 2: The Liouvillian matrix is the transpose of the coefficient matrix of the system of commutator equations.

Proof: The action of the Liouvillian onto the density operator can be represented in matrix form by $\mathbf{L}\cdot\boldsymbol{\rho}$, the product of the Liouvillian matrix and the column of the density matrix elements. On the other hand, the action of the Liouvillian is to form the commutator with the Hamiltonian. The result of this action on the column $\mathbf{u}$ of basis operators can be formally described by the multiplication with a matrix $\mathbf{M}$ as $\mathbf{M}\cdot\mathbf{u}$. An explicit formulation of that is Eq. (19) in the Main Part.

Following Eq. (S3), we identifiy $\mathbf{M}$ with $\mathbf{L}^{\mathrm{T}}$. (19).

This proves proposition 2.

■

Proposition 3: The coefficient matrix of the propagation formulae is the transposed propagator matrix.

Proof: The action of the propagator $\mathbf{U}$ can be expressed by its action on the density column matrix as

$$\boldsymbol{\rho}(t) = \mathbf{U}\cdot\boldsymbol{\rho}_0 \qquad \text{(S4)}$$

where we use the column basis {(1,0,0,…,0), (0,1,0,…,0),…}.

In contrast, the propagation formulae use the dual basis $\{\hat{u}_1,...,\hat{u}_N\}$. Again, we describe the time evolution by the multiplication with a matrix $\mathbf{M}'$ as

$$\mathbf{u} \quad \rightarrow \quad \mathbf{M}'\cdot\mathbf{u} \qquad \text{(S5)}$$

Corresponding to proposition 1, we identify $\mathbf{M}'$ with the transpose $\mathbf{U}^{\mathrm{T}}$ of the propagator matrix. This proves proposition 3.

■

S1.2 Orthogonality of Hermitian operators to its commutators

Proposition 4: $\hat{A}$ and $\left[\hat{B},\hat{A}\right]$ are orthogonal if $\hat{A}$ and $\hat{B}$ are Hermitian.

Proof: $\left(\hat{A},\hat{B}\right) = \mathrm{Tr}\left\{\mathbf{A}^{\dagger}.\left(\mathbf{A}.\mathbf{B}-\mathbf{B}.\mathbf{A}\right)\right\} = \mathrm{Tr}\left\{\mathbf{A}.\mathbf{A}.\mathbf{B}\right\} - \mathrm{Tr}\left\{\mathbf{A}.\mathbf{B}.\mathbf{A}\right\} = 0$

because a cyclic permutation within a matrix product does not change its trace.

■

S1.3 Rules for trace calculations

Firstly, we consider a product of n Cartesian components of the individual spin operators, as for example $\hat{I}_x$, $\hat{I}_y\hat{S}_z$, or $\hat{I}_z\hat{S}_{1x}\hat{S}_{2x}$, in a system of N spins. The spins are numbered here by a superscript, i.e. we deal with the operators $\hat{1}^{(\alpha)}, \hat{I}_x^{(\alpha)}, \hat{I}_y^{(\alpha)}, \hat{I}_y^{(\alpha)}$ $(\alpha \in \{1..N\})$ for the Cartesian components of the α-th spin. $\hat{1}^{(\alpha)}$ is the unit operator in the subspace of the α-th spin. The matrix of a single-spin operator in the *whole* space can be written as $\mathbf{1}^{(1)} \otimes \mathbf{1}^{(2)} \otimes \ldots \otimes \mathbf{I}_\gamma^{(\alpha)} \otimes \ldots \otimes \mathbf{1}^{(N)}$ where the bold symbols denote the matrices assigned to the corresponding operators, $\otimes$ denotes the Kronecker product and $\gamma \in \{x, y, z\}$.

From the rule $\mathrm{Tr}(\mathbf{A} \otimes \mathbf{B}) = \mathrm{Tr}\,\mathbf{A} \cdot \mathrm{Tr}\,\mathbf{B}$ it will be clear that the trace of the matrix of any product of Cartesian angular momentum operators in odd powers is zero.

To calculate the norm, we have to calculate the traces of the matrix squares (all operators considered in this paragraph are Hermitian). In the single-spin subspaces we get:

$$\mathrm{Tr}\,\mathbf{1}^{(\alpha)} = 2\,; \quad \mathrm{Tr}\left(\mathbf{I}_x^{(\alpha)}\right)^2 = \mathrm{Tr}\left(\mathbf{I}_y^{(\alpha)}\right)^2 = \mathrm{Tr}\left(\mathbf{I}_z^{(\alpha)}\right)^2 = \mathrm{Tr}\left(\frac{1}{4}\cdot\mathbf{1}\right) = \frac{1}{2}$$

The matrix of a product of n spin operators in a N-spin space is the Kronecker product of n spin matrices and N-n unit matrices. Its trace is therefore $(1/2)^n \cdot 2^{N-n} = 2^{N-2n}$, the norm is the square root:

$$\left\|\hat{I}_\gamma^{(\alpha)}\right\| = 2^{\frac{N}{2}-1}\,; \qquad \left\|\hat{I}_\gamma^{(\alpha)}\hat{I}_\delta^{(\beta)}\right\| = 2^{\frac{N}{2}-2}\,; \qquad \left\|\hat{I}_\gamma^{(\alpha)}\hat{I}_\delta^{(\beta)}\hat{I}_\varepsilon^{(\omega)}\right\| = 2^{\frac{N}{2}-3} \quad \text{etc.}$$

The following table summarized the trace values for some N and n:

$N \rightarrow$ / $n \downarrow$	1	2	3	4
1	$\frac{1}{\sqrt{2}}$	1	$\sqrt{2}$	2
2		$\frac{1}{2}$	$\frac{1}{\sqrt{2}}$	1
3			$\frac{1}{2\sqrt{2}}$	$\frac{1}{2}$

Secondly, the norm of the sum of two orthogonal operators is

$$\left\|\hat{A}+\hat{B}\right\| = \sqrt{\mathrm{Tr}\left[\left(\mathbf{A}^\dagger + \mathbf{B}^\dagger\right)\left(\mathbf{A}+\mathbf{B}\right)\right]} = \sqrt{\mathrm{Tr}\left(\mathbf{A}^\dagger.\mathbf{A}\right) + \mathrm{Tr}\left(\mathbf{B}^\dagger.\mathbf{B}\right)} = \sqrt{\left\|\hat{A}\right\|^2 + \left\|\hat{B}\right\|^2}$$

S2 Matrix exponentials

For the two-dimensional case, we make use of the property that the square of the matrix in the exponent is proportional to the unit matrix:

$$\begin{pmatrix} 0 & \lambda t \\ -\lambda t & 0 \end{pmatrix}^2 = -\lambda^2 t^2 \begin{pmatrix} 1 & 0 \\ 0 & 1 \end{pmatrix}$$

We then obtain for higher powers

$$\begin{pmatrix} 0 & \lambda t \\ -\lambda t & 0 \end{pmatrix}^{2n} = \left(-\lambda^2 t^2\right)^n \begin{pmatrix} 1 & 0 \\ 0 & 1 \end{pmatrix} \quad \text{and} \quad \begin{pmatrix} 0 & \lambda t \\ -\lambda t & 0 \end{pmatrix}^{2n+1} = \lambda t \left(-\lambda^2 t^2\right)^n \begin{pmatrix} 0 & 1 \\ -1 & 0 \end{pmatrix}$$

and for the matrix exponential:

$$\begin{aligned} \exp\begin{pmatrix} 0 & \lambda t \\ -\lambda t & 0 \end{pmatrix} &= \sum_{n=0}^{\infty} \frac{1}{(2n)!} \begin{pmatrix} 0 & i\lambda t \\ -i\lambda t & 0 \end{pmatrix}^{2n} + \sum_{n=0}^{\infty} \frac{1}{(2n+1)!} \begin{pmatrix} 0 & i\lambda t \\ -i\lambda t & 0 \end{pmatrix}^{2n+1} \\ &= \begin{pmatrix} 1 & 0 \\ 0 & 1 \end{pmatrix} \sum_{n=0}^{\infty} \frac{(-)^n \lambda^{2n} t^{2n}}{(2n)!} + \begin{pmatrix} 0 & 1 \\ -1 & 0 \end{pmatrix} \sum_{n=0}^{\infty} \frac{(-)^n \lambda^{2n+1} t^{2n+1}}{(2n+1)!} \\ &= \begin{pmatrix} 1 & 0 \\ 0 & 1 \end{pmatrix} \cos\lambda t + \begin{pmatrix} 0 & 1 \\ -1 & 0 \end{pmatrix} \sin\lambda t = \underline{\underline{\begin{pmatrix} \cos\lambda t & \sin\lambda t \\ -\sin\lambda t & \cos\lambda t \end{pmatrix}}} \end{aligned}$$

Matrix exponentials of larger matrices can be obtained in a number of ways, for example using the Cayley-Hamilton theorem, the Putzer algorithm or Sylvester's formula. Computer algebra programs such as Mathematica may be useful.

For applications corresponding to subspaces with dimension up to 6, the corresponding results for the exponentialization of the Liouvillians shown in this paper can be used as a template.

S3 Application examples

S3.1 "Zero-dimensional" case

If the Hamiltonian commutes with the operator characterizing the state of the spin system, there is no change of the state. This holds for:

S3.1.1 Example 0D-1: Total *z* magnetization under homonuclear dipolar interaction

$$\left[\hat{H}_{II}, \hat{I}_{1z} + \hat{I}_{2z}\right] = 0 \quad \rightarrow \quad \hat{I}_{1z} + \hat{I}_{2z} \xrightarrow{\hat{H}_{II} \cdot t} \hat{I}_{1z} + \hat{I}_{2z} \tag{S6}$$

The statement is that the dipolar interaction within a pair of parallel spins ½ does not change this state, in contrast to antiparallel spins (see example 2D-4).

S3.1.2 Example 0D-2: Cross polarization, sum of *I* and *S* polarization (large rf powers)

$$\left[\hat{H}_{\mathrm{HH}}, \hat{S}_z + \hat{I}_z\right] = 0 \quad \rightarrow \quad \hat{S}_z + \hat{I}_z \xrightarrow{\hat{H}_{\mathrm{HH}} \cdot t} \hat{S}_z + \hat{I}_z \tag{S7}$$

Similar to the dipolar Hamiltonian, the Hartmann-Hahn Hamiltonian in the double-rotating frame does not change the sum of both states. However, this is not true if the rf power is limited, see example 3D-9.

These examples will play a role in the discussion of polarization transfer and cross-polarization effects below.

S3.2 Examples for the two-dimensional case

S3.2.1 Procedure as explained in the main part

$$\left|\begin{matrix}\left[\hat{H},\hat{A}\right]= i\lambda\hat{B} \\ \left[\hat{H},\hat{B}\right]=-i\lambda\hat{A}\end{matrix}\right| \rightarrow \boxed{\hat{A} \xrightarrow{\hat{H}\cdot t} \hat{A}\cos\lambda t+\hat{B}\sin\lambda t} \tag{S8}$$

S3.2.2 First group of examples: FID and spin interactions.

The initial state is parallel magnetization of spins I_1 and I_2, aligned transversal to $\mathbf{B}_0$.

S3.2.2.1 Example 2D-1: Homonuclear dipolar interaction between spins 1/2

Initial state and Hamiltonian: $\hat{A}=\hat{I}_{1x}+\hat{I}_{2x}$, $\hat{H}=\hat{H}_{II}$

Commutator equations:

$$\begin{aligned}\left[\hat{H}_{II},\hat{I}_{x1}+\hat{I}_{x2}\right] &= -\tfrac{3}{2}iD_{II}\left(2\hat{I}_{1y}\hat{I}_{2z}+2\hat{I}_{1z}\hat{I}_{2y}\right)\\ \left[\hat{H}_{II},2\hat{I}_{1y}\hat{I}_{2z}+2\hat{I}_{1z}\hat{I}_{2y}\right] &= \tfrac{3}{2}iD_{II}\left(\hat{I}_{x1}+\hat{I}_{x2}\right)\end{aligned} \tag{S9}$$

In a system of two spins ½, $\hat{I}_{1x}+\hat{I}_{2x}$ has the norm $\sqrt{2}$, $\hat{I}_{1y}\hat{I}_{2z}+\hat{I}_{1z}\hat{I}_{2y}$ has the norm $1/\sqrt{2}$. To ensure that the norms of both state operators are equal, we choose the assignment $\lambda=-\tfrac{3}{2}D_{II}$, $\hat{B}=2\hat{I}_{1y}\hat{I}_{2z}+2\hat{I}_{1z}\hat{I}_{2y}$. Insertion into Eq. (S8):

$$\boxed{\hat{I}_{1x}+\hat{I}_{2x} \xrightarrow{\hat{H}_{II}\cdot t} \left(\hat{I}_{1x}+\hat{I}_{2x}\right)\cos\frac{3}{2}D_{II}t \;-\; 2\left(\hat{I}_{1z}\hat{I}_{2y}+\hat{I}_{1y}\hat{I}_{2z}\right)\sin\frac{3}{2}D_{II}t} \tag{S10}$$

S3.2.2.2 Example 2D-2: Heteronuclear dipolar interaction between spins 1/2:

Commutator equations:

$$\begin{aligned}\left[\hat{H}_{IS},\hat{I}_x\right] &= -iD_{IS}\cdot 2\hat{I}_y\hat{S}_z\\ \left[\hat{H}_{IS},2\hat{I}_y\hat{S}_z\right] &= iD_{IS}\cdot\hat{I}_x\end{aligned} \tag{S11}$$

In a system of two spins ½, $\hat{I}_x$ has the norm 1 and $\hat{I}_y\hat{S}_z$ has the norm ½. To ensure that the norms of both state operators are equal, we choose the assignment $\lambda=-D_{IS}$, $\hat{B}=2\hat{I}_y\hat{S}_z$. Insertion into Eq. (S8):

$$\boxed{\hat{I}_x \xrightarrow{\hat{H}_{IS}\cdot t} \hat{I}_x \cos D_{IS}t \ - \ 2\hat{I}_y\hat{S}_z \sin D_{IS}t} \tag{S12}$$

S3.2.2.3 Example 2D-3: Quadrupolar interaction for spin 1:

Initial state and Hamiltonian: $\hat{A} = \hat{I}_x, \ \hat{H} = \hat{H}_Q$; commutator equations:

$$\begin{aligned} \left[\hat{H}_Q, \hat{I}_x\right] &= \omega_Q\left(\hat{I}_y\hat{I}_z + \hat{I}_z\hat{I}_y\right) \\ \left[\hat{H}_Q, \hat{I}_y\hat{I}_z + \hat{I}_z\hat{I}_y\right] &= -\omega_Q\,\hat{I}_x \end{aligned} \tag{S13}$$

For single spins 1, both $\hat{I}_x$ and $\hat{I}_y\hat{I}_z + \hat{I}_z\hat{I}_y$ have norm $\sqrt{2}$. We choose the assignment $\lambda = \omega_Q, \ \hat{B} = \hat{I}_y\hat{I}_z + \hat{I}_z\hat{I}_y$. Insertion into Eq. (S8):

$$\boxed{\hat{I}_x \xrightarrow{\hat{H}_Q\cdot t} \hat{I}_x \cos\omega_Q t \ + \ \left(\hat{I}_z\hat{I}_y + \hat{I}_y\hat{I}_z\right)\sin\omega_Q t} \tag{S14}$$

In all three cases of this first group of 2D examples there is an oscillation between an observable transversal magnetization and an antiphase state.

Remark: If we consider isotropic samples, which contain an orientation dependence of the angular frequencies of the oscillations, the total magnetization in the previous examples shows a strongly damped oscillation, which is the result of the so-called powder averaging.

S3.2.3 Second group: Polarization exchange

Polarization exchange occurs during both spin diffusion and cross polarization. Antiparallel coupled spins exchange their states when the total energy is maintained. This applies to spins whose resonance frequencies are the same, either like spins in the laboratory frame or unlike spins in the rotating frame under rf irradiation where the Hartmann-Hahn condition is fulfilled.

That is, the initial state in both cases is assumed to be an ensemble of pairs of antiparallel spins $\hat{\mathbf{I}}_1 \uparrow\downarrow \hat{\mathbf{I}}_2$ and $\hat{\mathbf{I}} \uparrow\downarrow \hat{\mathbf{S}}$ $(I_1, I_2, I, S = 1/2)$, characterized in the operator representation by $\hat{I}_{1z} - \hat{I}_{2z}$ and $\hat{S}_z - \hat{I}_z$, respectively. In this subsection, it is assumed that the relevant fields (external magnetic field in the first case, rf fields in the second case) are very large compared to the coupling frequency.

S3.2.3.1 Example 2D-4: Antiparallel like spins:

Initial state and Hamiltonian: $\hat{A} = \hat{I}_{1z} - \hat{I}_{2z}, \ \hat{H} = \hat{H}_{II}$ (secular part of the Hamiltonian; its use requires the assumption that the static magnetic field is large compared to the field exerted by one spin on the other.)

Commutator equations:

$$\begin{aligned} \left[\hat{H}_{II}, \hat{I}_{1z} - \hat{I}_{2z}\right] &= iD_{II}\left(2\hat{I}_{1x}\hat{I}_{2y} - 2\hat{I}_{1y}\hat{I}_{2x}\right) \\ \left[\hat{H}_{II}, 2\hat{I}_{1x}\hat{I}_{2y} - 2\hat{I}_{1y}\hat{I}_{2x}\right] &= -iD_{II}\left(\hat{I}_{1z} - \hat{I}_{2z}\right) \end{aligned} \tag{S15}$$

In a system of two spins ½, $\hat{I}_{1z} - \hat{I}_{2z}$ has the norm $\sqrt{2}$ and $\hat{I}_{1x}\hat{I}_{2y} - \hat{I}_{1y}\hat{I}_{2x}$ has the norm $1/\sqrt{2}$. To ensure that the norms of both state operators are equal, we choose $\lambda = D_{II}$, $\hat{B} = 2\hat{I}_{1x}\hat{I}_{2y} - 2\hat{I}_{1y}\hat{I}_{2x}$. Insertion into Eq. (S8):

$$\boxed{\hat{I}_{1z} - \hat{I}_{2z} \xrightarrow{\hat{H}_{II}\cdot t} \left(\hat{I}_{1z} - \hat{I}_{2z}\right)\cos D_{II}t \;+\; 2\left(\hat{I}_{1x}\hat{I}_{2y} - \hat{I}_{1y}\hat{I}_{2x}\right)\sin D_{II}t} \qquad \text{(S16)}$$

Note that the angular frequency of the oscillation is $-D_{II}$, not -(3/2) D_{II} as in the case of a FID under the same interaction (example 2D-1).

S3.2.3.2 Example 2D-5: Antiparallel unlike spins under Hartmann-Hahn condition:

Initial state and Hamiltonian: $\hat{A} = \hat{S}_z - \hat{I}_z$, $\hat{H} = \hat{H}_{\mathrm{HH}}$.

Commutator equations:

$$\begin{aligned} \left[\hat{H}_{\mathrm{HH}}, \hat{S}_z - \hat{I}_z\right] &= iD_{IS}\left(2\hat{I}_x\hat{S}_y - 2\hat{I}_y\hat{S}_x\right) \\ \left[\hat{H}_{\mathrm{HH}}, 2\hat{I}_x\hat{S}_y - 2\hat{I}_y\hat{S}_x\right] &= -iD_{IS}\left(\hat{S}_z - \hat{I}_z\right) \end{aligned} \qquad \text{(S17)}$$

In a system of two spins ½, $\hat{S}_z - \hat{I}_z$ has the norm $\sqrt{2}$ and $\hat{I}_x\hat{S}_y - \hat{I}_y\hat{S}_x$ has the norm $1/\sqrt{2}$ (see appendix). To ensure that the norms of both state operators are equal, we choose $\lambda = D_{IS}$, $\hat{B} = 2\hat{I}_x\hat{S}_y - 2\hat{I}_y\hat{S}_x$. Insertion into Eq. (S8):

$$\boxed{\hat{S}_z - \hat{I}_z \xrightarrow{\hat{H}_{\mathrm{HH}}\cdot t} \left(\hat{S}_z - \hat{I}_z\right)\cos D_{IS}t \;+\; 2\left(\hat{I}_x\hat{S}_y - \hat{I}_y\hat{S}_x\right)\sin D_{IS}t} \qquad \text{(S18)}$$

Note that the sum of the polarizations of both spins within a pair is constant under these circumstances, because the sums of the *z* components of both spin operators commute with the corresponding Hamiltonians, see examples 0D-1 and 0D-2.

Example 2D-4 can be regarded as an elementary step of the spin diffusion. It represents the ensemble average over the individual spin flip processes. Although the spin diffusion generally exhibits a non-oscillatory course, oscillations can be detected at the beginning. For longer times, however, the multi-spin interactions will suppress these oscillations (Zhang et al. 1992, Roos et al. 2012).

The use of this form of the Hamiltonian (see Main Part, Appendix, Eq. (B4) is associated with the assumptions that (i) the Hartmann-Hahn condition is fulfilled, and (ii) the rf field strengths at both channels are large compared to the coupling fields. If we consider that at least one of these assumptions is not fulfilled, we encounter a problem leading to a subspace with dimensions larger than 2, see below.

S3.3 Examples for the three-dimensional case

S3.3.1 Procedure as explained in the main part

The system of commutator equations

$$\begin{aligned}
\left[\hat{H},\hat{A}\right] &= && i a \cdot \hat{B} && \\
\left[\hat{H},\hat{B}\right] &= -i a \cdot \hat{A} && && +i b \cdot \hat{C} \\
\left[\hat{H},\hat{C}\right] &= && -i b \cdot \hat{B} &&
\end{aligned} \tag{S19}$$

gives the following propagation rules (with $q = \sqrt{a^2 + b^2}$):

$$\boxed{\begin{array}{lll}
\hat{A} & \xrightarrow{\hat{H}\cdot t} & \hat{A}\cdot\dfrac{b^2 + a^2\cos qt}{q^2} + \hat{B}\cdot\dfrac{a}{q}\sin qt + \hat{C}\cdot\dfrac{ab}{q^2}(1-\cos qt) \\
\hat{B} & \xrightarrow{\hat{H}\cdot t} & \hat{B}\cdot\cos qt + \hat{C}\cdot\dfrac{b}{q}\sin qt - \hat{A}\cdot\dfrac{a}{q}\sin qt \\
\hat{C} & \xrightarrow{\hat{H}\cdot t} & \hat{C}\cdot\dfrac{a^2 + b^2\cos qt}{q^2} + \hat{A}\cdot\dfrac{ab}{q^2}(1-\cos qt) - \hat{B}\cdot\dfrac{b}{q}\sin qt
\end{array}} \tag{S20}$$

For the special case *a* = *b* we obtain the following propagation formulae:

$$\boxed{\begin{array}{lll}
\hat{A} & \xrightarrow{\hat{H}\cdot t} & \hat{A}\cdot\cos^2\dfrac{qt}{2} + \hat{B}\cdot\dfrac{1}{\sqrt{2}}\sin qt + \hat{C}\cdot\sin^2\dfrac{qt}{2} \\
\hat{B} & \xrightarrow{\hat{H}\cdot t} & \hat{B}\cdot\cos qt + \hat{C}\cdot\dfrac{1}{\sqrt{2}}\sin qt - \hat{A}\cdot\dfrac{1}{\sqrt{2}}\sin qt \\
\hat{C} & \xrightarrow{\hat{H}\cdot t} & \hat{C}\cdot\cos^2\dfrac{qt}{2} + \hat{A}\cdot\cos^2\dfrac{qt}{2} - \hat{B}\cdot\dfrac{1}{\sqrt{2}}\sin qt
\end{array}} \tag{S21}$$

S3.3.2 First group: rf irradiation at the observe channel, initially equilibrium, magnetization along B_0.

Here we follow the magnetization of the *I* spins during rf irradiation under the influence of another interaction.

S3.3.2.1 Example 3D-1: rf irradiation on isolated spins under resonance offset (off-resonance nutation experiment)

Initial state and Hamiltonian to be replaced in Eq. (S34): $\hat{A} = \hat{I}_z,\ \hat{H} = \hat{H}_\Delta + \hat{H}_{Ix}$.

$$\begin{aligned}
\left[\hat{H}_\Delta + \hat{H}_{Ix}, \hat{I}_z\right] &= i\omega_1 \hat{I}_y \\
\left[\hat{H}_\Delta + \hat{H}_{Ix}, \hat{I}_y\right] &= -i\omega_1 \hat{I}_z + i\Delta\omega\hat{I}_x \\
\left[\hat{H}_\Delta + \hat{H}_{Ix}, \hat{I}_x\right] &= -i\Delta\omega \hat{I}_y
\end{aligned} \tag{S22}$$

After the third application of the commutator with the Hamiltonian, the result contains only $\hat{I}_y$ which appears already in the first commutator equation. So the iteration stops here.

In this single-spin system, the norm of all three operators is $1/\sqrt{2}$. Hence, we assign $\hat{B}=\hat{I}_y,\ \hat{C}=\hat{I}_x,\ a=\omega_1$ and $b=\Delta\omega$. Inserting this into the first of the propagation rules in Eq. (S20), we obtain the following propagation rule for $\hat{I}_z$:

$$\hat{I}_z \xrightarrow{\left(\hat{H}_{Ix}+\hat{H}_{\Delta\omega}\right)\cdot t} \hat{I}_z\cdot\frac{\Delta\omega^2+\omega_{1I}^2\cos qt}{q^2} + \hat{I}_y\cdot\frac{\omega_{1I}}{q}\sin qt + \hat{I}_x\cdot\frac{\omega_{1I}\Delta\omega}{q^2}\left(1-\cos qt\right) \tag{S23}$$

with $q=\sqrt{\omega_{1I}^2+\Delta\omega^2}$.

S3.3.2.2 Example 3D-2: rf irradiation under heteronuclear dipolar interaction

Initial state and Hamiltonian: $\hat{A}=\hat{I}_z,\ \hat{H}=\hat{H}_{Ix}+\hat{H}_{IS}$.

Commutator equations corresponding to Eq. (S19):

$$\begin{aligned}\left[\hat{H}_{Ix}+\hat{H}_{IS},\hat{I}_z\right] &= i\omega_{1I}\hat{I}_y \\ \left[\hat{H}_{Ix}+\hat{H}_{IS},\hat{I}_y\right] &= -i\omega_{1I}\hat{I}_z + iD_{IS}\cdot 2\hat{I}_x\hat{S}_z \\ \left[\hat{H}_{Ix}+\hat{H}_{IS},2\hat{I}_x\hat{S}_z\right] &= i\pi J\hat{I}_y\end{aligned} \tag{S24}$$

In a system of two spins ½, $\hat{I}_z$ and $\hat{I}_y$ have the norm 1 and $\hat{I}_x\hat{S}_z$ has the norm ½. To ensure equal norms of all state operators, we assign $\hat{B}=\hat{I}_y,\ \hat{C}=2\hat{I}_x\hat{S}_z,\ a=\omega_{1I},\ b=D_{IS}$. This is inserted into Eq. (S20):

$$\hat{I}_z \xrightarrow{\left(\hat{H}_{Ix}+\hat{H}_{IS}\right)\cdot t} \hat{I}_z\cdot\frac{D_{IS}^2+\omega_{1I}^2\cos qt}{q^2} + \hat{I}_y\cdot\frac{\omega_{1I}}{q}\sin qt + 2\hat{I}_x\hat{S}_z\cdot\frac{\omega_{1I}D_{IS}}{q^2}\left(1-\cos qt\right) \tag{S25}$$

with $q=\sqrt{\omega_{1I}^2+D_{IS}^2}$.

(The case of homonuclear interaction under rf irradiation leads to a 4D problem, see example 4D-2.) The similarity of both equations (S23) and (S25) is evident. For $\Delta\omega\to 0$ and $D_{IS}\to 0$, respectively, they merge into an equation describing a rotation in the *yz* plane.

It should be mentioned that Eq. (S23) can also be obtained in two other ways: (i) Classical vector model: the motion of the magnetization is regarded as a precession around the effective field $\mathbf{B}_{\mathrm{eff}}=\mathbf{B}_1+\Delta\mathbf{B}_0$, and (ii) Coordinate transformation as rotation around the *y* axis by an angle of $\arctan\left(\Delta\omega/\omega_{1I}\right)$, and subsequent application of the POF rules.

Both Eqs. (S23) and (S25) reflect the well-known fact that a total inversion of the magnetization with a single rectangular pulse is only possible if the offset or the coupling is zero. In addition, coupling and resonance offset change the pulse duration required to reach both maximum and zero *y* magnetization to shorter times:

$$\sqrt{\omega_{1I}^2 + C^2} \cdot \tau = \begin{cases} \pi/2 & \text{for maximum } M_y \\ \pi & \text{for zero-crossing of } M_y \end{cases} \qquad \text{(S26)}$$

where $C = \Delta\omega$ for Eq. (S23) and $C = D_{IS}$ for Eq. (S25).

To obtain the corresponding scalar-interaction equation, replace D_{IS} with $-\pi J$.

S3.3.3 Second group: rf irradiation during FID influenced by another interaction, initial state: transversal magnetization

S3.3.3.1 Example 3D-3: Heteronuclear cw decoupling with finite rf power

Here we consider ensembles of isolated pairs of coupled unequal spins *I*, *S*. The process starts with *I* magnetization along the *x* axis in the rotating frame. The *S* spins are rf irradiated.

Initial state and Hamiltonian: $\hat{A} = \hat{I}_x$, $\hat{H} = \hat{H}_{Sx} + \hat{H}_{IS}$.

Commutator equations corresponding to Eq. (S19):

$$\begin{aligned} \left[\hat{H}_{Sx} + \hat{H}_{IS}, \hat{I}_x\right] &= & -iD_{\mathrm{IS}} \cdot 2\hat{I}_y\hat{S}_z & \\ \left[\hat{H}_{Sx} + \hat{H}_{IS}, 2\hat{I}_y\hat{S}_z\right] &= iD_{\mathrm{IS}} \cdot \hat{I}_x & & +i\omega_{1S} \cdot 2\hat{I}_y\hat{S}_y \\ \left[\hat{H}_{Sx} + \hat{H}_{IS}, 2\hat{I}_y\hat{S}_y\right] &= & -i\omega_{1S} \cdot 2\hat{I}_y\hat{S}_z & \end{aligned} \qquad \text{(S27)}$$

In a system of two spins ½, $\hat{I}_x$ has the norm 1, $\hat{I}_y\hat{S}_z$ and $\hat{I}_y\hat{S}_y$ have the norm ½. To ensure that the norms of all state operators are the same, we assign $\hat{B} = \hat{I}_y\hat{S}_z$, $\hat{C} = 2\hat{I}_y\hat{S}_y$, $a = -D_{IS}$ and $b = \omega_{1S}$. Inserting that into Eq. (S20) gives

$$\boxed{\hat{I}_x \xrightarrow{\left(\hat{H}_{IS} + \hat{H}_{Sx}\right)\cdot t} \hat{I}_x \cdot \frac{\omega_{1S}^2 + D_{\mathrm{IS}}^2 \cos qt}{q^2} - 2\hat{I}_y\hat{S}_z \cdot \frac{D_{\mathrm{IS}}}{q} \sin q_3 t \quad - 2\hat{I}_x\hat{S}_z \cdot \frac{\omega_{1S} D_{\mathrm{IS}}}{q^2}\left(1 - \cos qt\right)} \qquad \text{(S28)}$$

with $q = \sqrt{\omega_{1S}^2 + D_{IS}^2}$.

Equation (S28) describes a partial exchange of polarization between *x* magnetization and two antiphase states.

The decoupling effect is explained as follows: As ω_{1S} increases, the oscillation captures a decreasing fraction $D_{1S}^2 / \left(\omega_{1S}^2 + D_{\mathrm{IS}}^2\right)$ of the prefactor of $\hat{I}_x$, i.e. of the *x* magnetization, due to the fact that the average level grows proportionally to $\omega_{1S}^2 / \left(\omega_{1S}^2 + D_{\mathrm{IS}}^2\right)$ asymptotically to 1 for $\omega_{IS} \to \infty$. (For $\omega_{1S} \to 0$, Eq. (S28) approaches Eq. (S12) in example 2D-2. In other words, it

describes the FID for the case of heteronuclear dipolar interaction without decoupling, see also the black curve in Fig. 1 of the main part.) Orientational averaging leads to a fast decay of the oscillation which produces a broad resonance after Fourier transformation. Such an oscillation has been observed in DIPSHIFT experiments (Kurz et al., 2013), albeit under MAS conditions. This is illustrated in Fig. 1 (main part) by the curves for different *b* (corresponding to ω_{1S} in this example). The constant component is subject to relaxation damping and chemical-shift-induced oscillation on a longer time scale and produces a more or less narrow line.

For obtaining the corresponding equation for the *J* coupling, replace D_{IS} with $-\pi J$.

S3.3.3.2 Example 3D-4: In-resonance spin locking and heteronuclear dipolar coupling

Again, we consider ensembles of isolated pairs of coupled nonequal spins *I*, *S* starting with *I* magnetization along *x* axis in the rotating frame. Then the evolution under rf irradiation along *x* in the observe channel is recorded.

Initial state and Hamiltonian: $\hat{A} = \hat{I}_x,\ \hat{H} = \hat{H}_{Ix} + \hat{H}_{IS}$.

Commutator equations corresponding to Eq. (S19):

$$\begin{aligned}
\left[\hat{H}_{Ix} + \hat{H}_{IS}, \hat{I}_x\right] &= -iD_{IS} \cdot 2\hat{I}_y\hat{S}_z \\
\left[\hat{H}_{Ix} + \hat{H}_{IS}, 2\hat{I}_y\hat{S}_z\right] &= iD_{IS} \cdot \hat{I}_x - i\omega_{1I} \cdot 2\hat{I}_z\hat{S}_z \\
\left[\hat{H}_{Ix} + \hat{H}_{IS}, 2\hat{I}_z\hat{S}_z\right] &= i\omega_{1I} \cdot 2\hat{I}_y\hat{S}_z
\end{aligned} \tag{S29}$$

In a system of two spins ½, $\hat{I}_x$ has the norm 1, $\hat{I}_y\hat{S}_z$ and $\hat{I}_z\hat{S}_z$ have the norm ½. To ensure that the norms of all state operators are equal, we assign $\hat{B} = \hat{I}_y\hat{S}_z,\ \hat{C} = 2\hat{I}_z\hat{S}_z,\ a = -D_{IS}$ and $b = -\omega_{1I}$. Inserting that into Eq. (S20) gives

$$\boxed{\hat{I}_x \xrightarrow{\left(\hat{H}_{Ix} + \hat{H}_{IS}\right)\cdot t} \hat{I}_x \cdot \frac{\omega_{1I}^2 + D_{IS}^2 \cos qt}{q^2} - 2\hat{I}_y\hat{S}_z \cdot \frac{D_{IS}}{q}\sin qt + 2\hat{I}_z\hat{S}_z \cdot \frac{\omega_{1I}D_{IS}}{q^2}\left(1 - \cos qt\right)} \tag{S30}$$

(The oscillation frequency is indeed equal to that for the nutation example 3D-2, see equation (S25).)

S3.3.3.3 Example 3D-5: In-resonance spin locking and homonuclear dipolar coupling

Here we consider ensembles of isolated pairs of coupled equal spins $I_1 = I_2 = ½$. The process starts again with *I* magnetization along the *x*-axis in the rotating frame. Then the evolution under rf irradiation along *x* in the observe channel is recorded.

Initial state and Hamiltonian: $\hat{A} = \hat{I}_{1x} + \hat{I}_{2x},\ \hat{H} = \hat{H}_{Ix} + \hat{H}_{II}$.

Commutator equations corresponding to Eq. (S19):

$$\left[\hat{H}_{Ix}+\hat{H}_{II},\hat{I}_{1x}+\hat{I}_{2x}\right] = -\frac{3}{2}iD_{\mathrm{ii}}\left(2\hat{I}_{1y}\hat{I}_{2z}+2\hat{I}_{1z}\hat{I}_{2y}\right)$$
$$\left[\hat{H}_{Ix}+\hat{H}_{II},2\hat{I}_{1y}\hat{I}_{2z}+2\hat{I}_{1z}\hat{I}_{2y}\right] = \frac{3}{2}iD_{\mathrm{ii}}\left(\hat{I}_{1x}+\hat{I}_{2x}\right) - 2i\omega_1\left(2\hat{I}_{1z}\hat{I}_{2z}-2\hat{I}_{1y}\hat{I}_{2y}\right) \tag{S31}$$
$$\left[\hat{H}_{Ix}+\hat{H}_{II},2\hat{I}_{1z}\hat{I}_{2z}-2\hat{I}_{1y}\hat{I}_{2y}\right] = 2i\,\omega_1\left(2\hat{I}_{1y}\hat{I}_{2z}+2\hat{I}_{1z}\hat{I}_{2y}\right)$$

System of spins ½: $\hat{I}_{1x}+\hat{I}_{2x}$ has norm $\sqrt{2}$, $\hat{I}_{1y}\hat{I}_{2z}+\hat{I}_{1z}\hat{I}_{2y}$ and $\hat{I}_{1z}\hat{I}_{2z}-\hat{I}_{1y}\hat{I}_{2y}$ have norm $1/\sqrt{2}$. To ensure that the norms of all state operators are equal, we assign $\hat{B}=2\hat{I}_{1y}\hat{I}_{2z}+2\hat{I}_{1z}\hat{I}_{2y},\ \hat{C}=2\hat{I}_{1z}\hat{I}_{2z}-2\hat{I}_{1y}\hat{I}_{2y},\ a=-\frac{3}{2}D_{IS}$ and $b=-2\omega_1$. Inserting that into Eq. (S20) gives

$$\hat{I}_{1x}+\hat{I}_{2x} \xrightarrow{\left(\hat{H}_{Ix}+\hat{H}_{II}\right)\cdot t} \left(\hat{I}_{1x}+\hat{I}_{2x}\right)\cdot\frac{4\omega_{1I}^2+\frac{9}{4}D_{II}^2\cos qt}{q^2} -2\left(\hat{I}_{1y}\hat{I}_{2z}+\hat{I}_{1z}\hat{I}_{2y}\right)\cdot\frac{3D_{II}}{2q}\sin qt +2\left(\hat{I}_{1z}\hat{I}_{2z}-\hat{I}_{1y}\hat{I}_{2y}\right)\cdot\frac{3\omega_{1I}D_{II}}{q^2}\left(1-\cos qt\right) \tag{S32}$$

with $q=\sqrt{\frac{9}{4}D_{\mathrm{II}}^2+4\omega_{1I}^2}$.

Comments on examples 3D-4 and 3D-5:

- In the limiting case $\omega_{1I}\rightarrow 0$, both equations, as expected, approach the corresponding rf-free FID's given by examples 2D-1 and 2D-2, respectively.
- Analogous to the other 3D examples, the oscillation takes place around a level that increases with ω_{1I}. The latter corresponds to the spin-locked part of the transversal magnetization. At the same time, the amplitude of the oscillation is reduced.
- These oscillations are observed at the onset of spin-lock experiments (Krushelnitsky et al. 2018, 2023) and have been described theoretically by Garroway (1979) and McArthur et al (1969). Due to their orientation dependence, they decay rather quickly in a powder sample, but can be refocussed in MAS experiments.
- The propagation formulae (S25) and (S30) refer to the same Hamiltonian. As a consequence, the oscillation frequencies are the same. However, the different initial states lead to different subspaces and thus to different propagation formulae.

S3.3.4 Third group: Polarization transfer; the relevant field strengths are much larger than the coupling frequency

This situation is very similar to those treated as 2D cases (examples 2D-4 and 2D-5). The difference is again in the initial states. Instead of the antiparallel initial orientation as above, here one spin of the pairs is polarized, the other is not. The initial states are now described by $\hat{I}_{1z}$ and $\hat{S}_z$, respectively. Since they are not elements of the 2D subspaces of the examples above, the motion now takes place in other subspaces, which turn out to be three-dimensional.

S3.3.4.1 Example 3D-6: Equal spins:

Initial state and Hamiltonian: $\hat{A}=\hat{I}_{1z},\ \hat{H}=\hat{H}_{II}$

Commutator equations corresponding to Eq. (S19):

$$\begin{aligned}
\left[\hat{H}_{II},\hat{I}_{1z}\right] &= && iD_{II}\left(\hat{I}_{1x}\hat{I}_{2y}-\hat{I}_{1y}\hat{I}_{2x}\right) \\
\left[\hat{H}_{II},\hat{I}_{1x}\hat{I}_{2y}-\hat{I}_{1y}\hat{I}_{2x}\right] &= -\frac{i}{2}D_{II}\hat{I}_{1z} && && +\frac{i}{2}D_{II}\hat{I}_{2z} \\
\left[\hat{H}_{II},\hat{I}_{2z}\right] &= && -iD_{II}\left(\hat{I}_{1x}\hat{I}_{2y}-\hat{I}_{1y}\hat{I}_{2x}\right)
\end{aligned} \tag{S33}$$

System of two spins ½: $\hat{I}_{1z}$ and $\hat{I}_{2z}$ have the norm 1, $\hat{I}_{1x}\hat{I}_{2y}-\hat{I}_{1y}\hat{I}_{2x}$ has the norm $1/\sqrt{2}$. To ensure that the norms of all state operators are equal, we assign $\hat{B}=\sqrt{2}\left(\hat{I}_{1x}\hat{I}_{2y}-\hat{I}_{1y}\hat{I}_{2x}\right),\ \hat{C}=\hat{I}_{2z},\ a=b=D_{II}/\sqrt{2},\ q=D_{II}$. Inserting that into Eq. (S21) gives

$$\boxed{\begin{aligned}\hat{I}_{1z}\ \xrightarrow{\hat{H}_{II}\cdot t}\ & \hat{I}_{1z}\cdot\frac{1}{2}\left(1+\cos D_{II}t\right) \\ & +\left(\hat{I}_{1x}\hat{I}_{2y}-\hat{I}_{1y}\hat{I}_{2x}\right)\sin D_{II}t\ +\hat{I}_{2z}\cdot\frac{1}{2}\left(1-\cos D_{II}t\right)\end{aligned}} \tag{S34}$$

S3.3.4.2 Example 3D-7: Unequal spins under Hartmann-Hahn condition, infinite rf power

Initial state and Hamiltonian: $\hat{A}=\hat{S}_{z},\ \hat{H}=\hat{H}_{\mathrm{HH}}$.

Commutator equations corresponding to Eq. (S19):

$$\begin{aligned}
\left[\hat{H}_{\mathrm{HH}},\hat{S}_{z}\right] &= && iD_{\mathrm{IS}}\left(\hat{I}_{x}\hat{S}_{y}-\hat{I}_{y}\hat{S}_{x}\right) \\
\left[\hat{H}_{\mathrm{HH}},\hat{I}_{x}\hat{S}_{y}-\hat{I}_{y}\hat{S}_{x}\right] &= -\frac{i}{2}D_{\mathrm{IS}}\hat{S}_{z} && && +\frac{i}{2}D_{\mathrm{IS}}\hat{I}_{z} \\
\left[\hat{H}_{\mathrm{HH}},\hat{I}_{z}\right] &= && -iD_{\mathrm{IS}}\left(\hat{I}_{x}\hat{S}_{y}-\hat{I}_{y}\hat{S}_{x}\right)
\end{aligned} \tag{S35}$$

System of two spins ½: $\hat{I}_{z}$ and $\hat{S}_{z}$ have the norm 1, $\hat{I}_{x}\hat{S}_{y}-\hat{I}_{y}\hat{S}_{x}$ has the norm $1/\sqrt{2}$. To ensure that the norms of all state operators are equal, we assign $\hat{B}=\sqrt{2}\left(\hat{I}_{x}\hat{S}_{y}-\hat{I}_{y}\hat{S}_{x}\right),\ \hat{C}=\hat{I}_{z},\ a=b=D_{IS}/\sqrt{2},\ q=D_{IS}$. Inserting that into Eq. (S21) gives

$$\boxed{\hat{S}_{z}\ \xrightarrow{\hat{H}_{\mathrm{HH}}\cdot t}\ \hat{S}_{z}\cdot\cos^{2}\frac{D_{IS}t}{2}+\left(\hat{I}_{x}\hat{S}_{y}-\hat{I}_{y}\hat{S}_{x}\right)\sin D_{IS}t+\hat{I}_{z}\cdot\sin^{2}\frac{D_{IS}t}{2}} \tag{S36}$$

Examples 3D-6 and 3D-7 deal with special case of the three-dimensional situation: The system of commutator equations (S19) contains here the case $a = b$ which has to be identified with the respective coupling frequency. The time evolution of the three coefficients is plotted in Fig. 2 of the main part.

These propagation rules can be obtained also as sum of rules obtained for other examples: [(S16) + (S6)]/2 → (S34) and [(S18) + (S7)]/2 → (S36).

S3.3.4.3 Example 3D-8: Cross polarization, finite rf power, possible deviation from Hartmann-Hahn condition, considering the difference of both polarizations

The double-rotating frame used in the paper of Hartmann and Hahn (1962) is unfavorable for the execution of this calculation, because the Hamiltonian contains time-varying terms. We can no longer assume that these can be neglected due to their high frequency. Instead, we work in the single rotating frame where there are no time-dependent terms in the Hamiltonian.

The Hamiltonian here is composed of the components belonging to the heteronuclear dipolar interaction as well as to the interaction between the rf radiation and the spin system: $\hat{H} \to \hat{H}_{Ix} + \hat{H}_{Sx} + \hat{H}_{IS}$. Both I = 1/2 and S = 1/2 spins are irradiated at their Larmor frequency along the x direction of their rotating frames. The antiparallel initial state is assigned as $\hat{A} = \hat{S}_x - \hat{I}_x$.

Commutator equations corresponding to Eq. (S19):

$$\begin{aligned}
\left[\hat{H}_{Ix} + \hat{H}_{Sx} + \hat{H}_{IS}, \hat{S}_x - \hat{I}_x\right] &= -iD_{\mathrm{IS}} \cdot 2\left(\hat{I}_z\hat{S}_y - \hat{I}_y\hat{S}_z\right) \\
\left[\hat{H}_{Ix} + \hat{H}_{Sx} + \hat{H}_{IS}, 2\hat{I}_z\hat{S}_y - 2\hat{I}_y\hat{S}_z\right] &= iD_{\mathrm{IS}}\left(\hat{S}_x - \hat{I}_x\right) - 2i\omega_\Delta\left(\hat{I}_z\hat{S}_z + \hat{I}_y\hat{S}_y\right) \\
\left[\hat{H}_{Ix} + \hat{H}_{Sx} + \hat{H}_{IS}, \hat{I}_z\hat{S}_z + \hat{I}_y\hat{S}_y\right] &= i\omega_\Delta\left(\hat{I}_z\hat{S}_y - \hat{I}_y\hat{S}_z\right)
\end{aligned} \tag{S37}$$

with the abbreviation $\omega_\Delta := \omega_{1S} - \omega_{1I}$.

System of two spins ½: $\hat{S}_x - \hat{I}_x$ has the norm $\sqrt{2}$, $\hat{I}_z\hat{S}_y - \hat{I}_y\hat{S}_z$ and $\hat{I}_z\hat{S}_z + \hat{I}_y\hat{S}_y$ have the norm $1/\sqrt{2}$. To ensure that the norms of all state operators are equal, we assign $\hat{B} = 2\left(\hat{I}_z\hat{S}_y - \hat{I}_y\hat{S}_z\right)$, $\hat{C} = 2\left(\hat{I}_z\hat{S}_z + \hat{I}_y\hat{S}_y\right)$, $a = D_{IS}$, $b = \omega_\Delta$, $q_\Delta = \sqrt{D_{\mathrm{IS}}^2 + \omega_\Delta^2}$. Inserting that into Eq. (S20) gives

$$\begin{aligned}
\hat{S}_x - \hat{I}_x \xrightarrow{\left(\hat{H}_{IS} + \hat{H}_{Ix} + \hat{H}_{Sx}\right)\cdot t} & \left(\hat{S}_x - \hat{I}_x\right) \cdot \frac{\omega_\Delta^2 + D_{\mathrm{IS}}^2 \cos q_\Delta t}{q_\Delta^2} \\
& - \left(\hat{I}_z\hat{S}_y - \hat{I}_y\hat{S}_z\right) \cdot 2\frac{D_{\mathrm{IS}}}{q_\Delta} \sin q_\Delta t \\
& + \left(\hat{I}_z\hat{S}_z + \hat{I}_y\hat{S}_y\right) \cdot \frac{\omega_\Delta D_{\mathrm{IS}}}{q_\Delta^2}\left(1 - \cos q_\Delta t\right)
\end{aligned} \tag{S38}$$

Comments:

- The CP oscillation frequency is no longer D_{IS} as in examples 2D-5 and 3D-7 but $q_\Delta = \sqrt{D_{\mathrm{IS}}^2 + \omega_\Delta^2}$ which increases with the difference of both rf field strengths.

- Moreover, only the relative part $D_{\mathrm{IS}}^2/q_\Delta^2$ of the total magnetization participates in the oscillation, i.e. the greater the deviation from the Hartmann-Hahn condition, the lower the maximum transmitted polarization.

In contrast to the case where the rf fields are very large (see examples 0D-1 and 0D-2), the sum of the two polarizations is no longer constant, as shown in the next example.

S3.3.4.4 Example 3D-9: Cross polarization, finite rf power, possible deviation from Hartmann-Hahn condition, considering the sum of both polarizations

Of course, the Hamiltonian is the same as in the preceding example. The difference is again in the initial state: $\hat{A} \to \hat{S}_x + \hat{I}_x, \hat{H} = \hat{H}_{Ix} + \hat{H}_{Sx} + \hat{H}_{IS}$.

Commutator equations corresponding to Eq. (S19):

$$\begin{aligned}
\left[\hat{H}_{Ix} + \hat{H}_{Sx} + \hat{H}_{IS}, \hat{S}_x + \hat{I}_x\right] &= -iD_{\mathrm{IS}} \cdot 2\left(\hat{I}_z\hat{S}_y + \hat{I}_y\hat{S}_z\right) \\
\left[\hat{H}_{Ix} + \hat{H}_{Sx} + \hat{H}_{IS}, 2\hat{I}_z\hat{S}_y + 2\hat{I}_y\hat{S}_z\right] &= iD_{\mathrm{IS}}\left(\hat{S}_x + \hat{I}_x\right) - 4i\omega_\varnothing\left(\hat{I}_z\hat{S}_z - \hat{I}_y\hat{S}_y\right) \\
\left[\hat{H}_{Ix} + \hat{H}_{Sx} + \hat{H}_{IS}, \hat{I}_z\hat{S}_z - \hat{I}_y\hat{S}_y\right] &= 2i\omega_\varnothing\left(\hat{I}_z\hat{S}_y + \hat{I}_y\hat{S}_z\right)
\end{aligned} \quad \text{(S39)}$$

with $\omega_\varnothing := (\omega_S + \omega_I)/2$. In system of two spins ½, $\hat{S}_x + \hat{I}_x$ has the norm $\sqrt{2}$, $\hat{I}_z\hat{S}_y + \hat{I}_y\hat{S}_z$ and $\hat{I}_z\hat{S}_z - \hat{I}_y\hat{S}_y$ have the norm $1/\sqrt{2}$. To ensure that the norms of all state operators are equal, we assign $\hat{B} = 2\left(\hat{I}_z\hat{S}_y + \hat{I}_y\hat{S}_z\right)$, $\hat{C} = 2\left(\hat{I}_z\hat{S}_z - \hat{I}_y\hat{S}_y\right)$, $a = D_{IS}$, $b = 2\omega_\varnothing$. Inserting that into Eq. (S20) gives

$$\hat{S}_x + \hat{I}_x \xrightarrow{\left(\hat{H}_{IS} + \hat{H}_{Ix} + \hat{H}_{Sx}\right)\cdot t} \left(\hat{S}_x + \hat{I}_x\right) \cdot \frac{4\omega_\varnothing^2 + D_{\mathrm{IS}}^2 \cos q_\varnothing t}{q_\varnothing^2} - \left(\hat{I}_z\hat{S}_y + \hat{I}_y\hat{S}_z\right) \cdot 2\frac{D_{\mathrm{IS}}}{q_\varnothing}\sin q_\varnothing t + \left(\hat{I}_z\hat{S}_z - \hat{I}_y\hat{S}_y\right) \cdot \frac{2\omega_\varnothing D_{\mathrm{IS}}}{q_\varnothing^2}\left(1 - \cos q_\varnothing t\right) \quad \text{(S40)}$$

(with the abbreviation $q_\varnothing := \sqrt{D_{\mathrm{IS}}^2 + 4\omega_\varnothing^2}$). The amplitude of this oscillation decreases if $\omega_\varnothing$ increases, see Fig. 1 (main part). This phenomenon is analogous to that which occurs during spin-lock and decoupling, see the corresponding examples above.

S3.3.4.5 Example 3D-10: LGCP in a three-spin system of the type IS_2, infinite rf power depolarization of I

This problem is treated again in the double rotating frame. Observed spin: I, two coupling spins: S_1 and S_2; dipolar frequencies D_1 for I-S_1 coupling, D_2 for I-S_2 coupling; no coupling between S_1 and S_2 (for example due to Lee-Goldburg irradiation).

Hamiltonian and initial state: $\hat{A} = \hat{I}_z$, $\hat{H} = \hat{H}_{\mathrm{HH2}} = -D_1\left(\hat{I}_x\hat{S}_{1x} + \hat{I}_y\hat{S}_{1y}\right) - D_2\left(\hat{I}_x\hat{S}_{2x} + \hat{I}_y\hat{S}_{2y}\right)$

Commutator equations corresponding to Eq. (S19):

$$\left[\hat{H},\hat{I}_z\right] = -iD_1\left(\hat{I}_x\hat{S}_{1y}-\hat{I}_y\hat{S}_{1x}\right)-iD_2\left(\hat{I}_x\hat{S}_{2y}-\hat{I}_y\hat{S}_{2x}\right)$$

$$\left[\hat{H},D_1\left(\hat{I}_x\hat{S}_{1y}-\hat{I}_y\hat{S}_{1x}\right)+D_2\left(\hat{I}_x\hat{S}_{2y}-\hat{I}_y\hat{S}_{2x}\right)\right] = \frac{i}{2}\left(D_1^2+D_2^2\right)\hat{I}_z-\frac{i}{2}\left(D_1^2\hat{S}_{1z}+D_2^2\hat{S}_{2z}\right) + 2i\,D_1D_2\,\hat{I}_z\left(\hat{S}_{1x}\hat{S}_{2x}+\hat{S}_{1y}\hat{S}_{2y}\right)$$

$$\left[\hat{H},D_1^2\hat{S}_{1z}+D_2^2\hat{S}_{2z}-4D_1D_2\,\hat{I}_z\left(\hat{S}_{1x}\hat{S}_{2x}+\hat{S}_{1y}\hat{S}_{2y}\right)\right] = i\left(D_1^2+D_2^2\right)\left[D_1\left(\hat{I}_x\hat{S}_{1y}-\hat{I}_y\hat{S}_{1x}\right) + D_2\left(\hat{I}_x\hat{S}_{2y}-\hat{I}_y\hat{S}_{2x}\right)\right]$$

This system contains basis operators with a more complex structure than above. In a system of three spins ½, the norms of the basis operators are:

$$\left\|\hat{I}_z\right\| = \sqrt{2}$$

$$\left\|D_1\left(\hat{I}_x\hat{S}_{1y}-\hat{I}_y\hat{S}_{1x}\right)+D_2\left(\hat{I}_x\hat{S}_{2y}-\hat{I}_y\hat{S}_{2x}\right)\right\| = \sqrt{D_1^2+D_2^2}$$

$$\left\|D_1^2\hat{S}_{1z}+D_2^2\hat{S}_{2z}-4D_1D_2\,\hat{I}_z\left(\hat{S}_{1x}\hat{S}_{2x}+\hat{S}_{1y}\hat{S}_{2y}\right)\right\| = \sqrt{2}\left(D_1^2+D_2^2\right)$$

To ensure that the norms of all state operators are equal, we assign

$$\hat{B}\rightarrow\frac{D_1\left(\hat{I}_x\hat{S}_{1y}-\hat{I}_y\hat{S}_{1x}\right)+D_2\left(\hat{I}_x\hat{S}_{2y}-\hat{I}_y\hat{S}_{2x}\right)}{\sqrt{D_1^2+D_2^2}}\sqrt{2}$$

$$\hat{C}\rightarrow\frac{D_1^2\hat{S}_{1z}+D_2^2\hat{S}_{2z}-4D_1D_2\,\hat{I}_z\left(\hat{S}_{1x}\hat{S}_{2x}+\hat{S}_{1y}\hat{S}_{2y}\right)}{D_1^2+D_2^2}.$$

$$a\rightarrow-\frac{1}{\sqrt{2}}\sqrt{D_1^2+D_2^2},\ b\rightarrow-\frac{1}{\sqrt{2}}\sqrt{D_1^2+D_2^2}$$

Inserting that into Eq. (S21), we get the propagation formula as

$$\hat{I}_z \xrightarrow{\hat{H}\cdot t} \hat{I}_z\cdot\cos^2\frac{\sqrt{D_1^2+D_2^2}}{2}t - \frac{D_1\left(\hat{I}_x\hat{S}_{1y}-\hat{I}_y\hat{S}_{1x}\right)+D_2\left(\hat{I}_x\hat{S}_{2y}-\hat{I}_y\hat{S}_{2x}\right)}{\sqrt{D_1^2+D_2^2}}\sin\sqrt{D_1^2+D_2^2}t + \frac{D_1^2\hat{S}_{1z}+D_2^2\hat{S}_{2z}-4D_1D_2\,\hat{I}_z\left(\hat{S}_{1x}\hat{S}_{2x}+\hat{S}_{1y}\hat{S}_{2y}\right)}{D_1^2+D_2^2}\sin^2\frac{\sqrt{D_1^2+D_2^2}}{2}t$$

This propagation formula shows the oscillatory exchange between the initial polarization and two states which are described in a more complex way using the spin operators. The description of the polarization of I by initially polarized S_1 and S_2 is not possible by this 3D consideration because $\hat{S}_{1z}$ and $\hat{S}_{2z}$ are not basis operators of this particular subspace.

S3.4 Examples for the four-dimensional case

S3.4.1 Procedure as explained in the main part

The system of commutator equations

$$\begin{aligned}
\left[\hat{H},\hat{A}\right] &= \quad i\,a\cdot\hat{B} \\
\left[\hat{H},\hat{B}\right] &= -i\,a\cdot\hat{A} \quad +i\,b\cdot\hat{C} \\
\left[\hat{H},\hat{C}\right] &= \quad -i\,b\cdot\hat{B} \quad \mp i\,a\cdot\hat{D} \\
\left[\hat{H},\hat{D}\right] &= \quad \pm i\,a\cdot\hat{C}
\end{aligned} \tag{S41}$$

gives the propagation rules in two different forms:

$$\begin{aligned}
\hat{A} \;\rightarrow\;& \hat{A}\cdot\frac{q_2\cos q_1 t + q_1\cos q_2 t}{2W} + \hat{B}\cdot\frac{a\left(\sin q_1 t + \sin q_2 t\right)}{2W} \\
&+ \hat{C}\cdot\frac{a\left(\cos q_2 t - \cos q_1 t\right)}{2W} \pm \hat{D}\cdot\frac{q_2\sin q_1 t - q_1\sin q_2 t}{2W} \qquad \text{(Version 1)} \\
=\;& \hat{A}\cdot\left(\cos Wt\,\cos\frac{bt}{2} + \frac{b}{2W}\sin Wt\,\sin\frac{bt}{2}\right) + \hat{B}\cdot\frac{a}{W}\sin Wt\,\cos\frac{bt}{2} \\
&- \hat{C}\cdot\frac{a}{W}\sin Wt\,\sin\frac{bt}{2} \mp \hat{D}\cdot\left(\cos Wt\,\sin\frac{bt}{2} - \frac{b}{2W}\sin Wt\,\cos\frac{bt}{2}\right) \qquad \text{(V. 2)}
\end{aligned} \tag{S42}$$

S3.4.1.1 Example 4D-1: J coupling, AB spin system

The two spins of this system have different positions in the spectrum, whereby their distance $\Delta v := \Delta\omega/(2\pi)$ is comparable to or smaller than the coupling constant J. The spectrometer frequency is assumed to be set at the midpoint between the two resonances. Then the initial state is represented by $\hat{A} = \hat{I}_{1x} + \hat{I}_{2x}$; the Hamiltonian is $\hat{H} = 2\pi J\,\mathbf{I}_1\mathbf{I}_2 + \frac{\Delta\omega}{2}\hat{I}_{1z} - \frac{\Delta\omega}{2}\hat{I}_{2z}$.

Commutator equations corresponding to Eq. (S41):

$$\begin{aligned}
\left[\hat{H},\hat{I}_{1x}+\hat{I}_{2x}\right] &= \frac{i\Delta\omega}{2}\left(\hat{I}_{1y}-\hat{I}_{2y}\right) \\
\left[\hat{H},\hat{I}_{1y}-\hat{I}_{2y}\right] &= -i\frac{\Delta\omega}{2}\left(\hat{I}_{1x}+\hat{I}_{2x}\right) + i2\pi J\left(2\hat{I}_{1z}\hat{I}_{2x}-2\hat{I}_{1x}\hat{I}_{2z}\right) \\
\left[\hat{H},2\hat{I}_{1z}\hat{I}_{2x}-2\hat{I}_{1x}\hat{I}_{2z}\right] &= -i2\pi J\left(\hat{I}_{1y}-\hat{I}_{2y}\right) - i\frac{\Delta\omega}{2}\left(2\hat{I}_{1y}\hat{I}_{2z}+2\hat{I}_{1z}\hat{I}_{2y}\right) \\
\left[\hat{H},2\hat{I}_{1y}\hat{I}_{2z}+2\hat{I}_{1z}\hat{I}_{2y}\right] &= i\frac{\Delta\omega}{2}\left(2\hat{I}_{1z}\hat{I}_{2x}-2\hat{I}_{1x}\hat{I}_{2z}\right)
\end{aligned} \tag{S43}$$

In this system of spins ½, $\hat{I}_{1x}+\hat{I}_{2x}$ and $\hat{I}_{1y}-\hat{I}_{2y}$ have the norm $\sqrt{2}$, $\hat{I}_{1z}\hat{I}_{2x}-\hat{I}_{1x}\hat{I}_{2z}$ and $\hat{I}_{1y}\hat{I}_{2z}+\hat{I}_{1z}\hat{I}_{2y}$ have norm $1/\sqrt{2}$. To ensure that the norms of all state operators are equal, we

assign $\hat{B}=\hat{I}_{1y}-\hat{I}_{2y}$, $\hat{C}=2\hat{I}_{1z}\hat{I}_{2x}-2\hat{I}_{1x}\hat{I}_{2z}$, $\hat{D}=2\hat{I}_{1y}\hat{I}_{2z}+2\hat{I}_{1z}\hat{I}_{2y}$, $a=\frac{1}{2}\Delta\omega$ and $b=2\pi J$. Inserting that into Eq. (S42), version 1, gives

$$\begin{aligned}\hat{I}_{1x}+\hat{I}_{2x} \quad \xrightarrow{\hat{H}\cdot t} \quad & \left(\hat{I}_{1x}+\hat{I}_{2x}\right)\cdot\frac{q_2\cos q_1 t+q_1\cos q_2 t}{W} \\ + \quad & \left(\hat{I}_{1y}-\hat{I}_{2y}\right)\cdot\frac{\Delta\omega}{2}\,\frac{\sin q_1 t+\sin q_2 t}{W} \\ + \quad & 2\left(\hat{I}_{1z}\hat{I}_{2x}-\hat{I}_{1x}\hat{I}_{2z}\right)\cdot\frac{\Delta\omega}{2}\,\frac{\cos q_1 t-\cos q_2 t}{W} \\ - \quad & 2\left(\hat{I}_{1y}\hat{I}_{2z}+\hat{I}_{1z}\hat{I}_{2y}\right)\cdot\frac{q_1\sin q_2 t-q_2\sin q_1 t}{W}\end{aligned} \tag{S44}$$

where $W=2\pi\sqrt{J^2+\Delta\nu^2}$ and $q_{1;2}=\pi\left(\sqrt{J^2+\Delta\nu^2}\mp J\right)$.

Antiphase terms appear as intermediate states during the time evolution. After Fourier transformation of this FID we obtain a spectrum with four resonances at the positions $\pm q_{1,2}/(2\pi)$, see Fig. S1. The prefactors $q_{1,2}/W$ describe the sums of the relative intensities of the outer and the inner two lines, respectively ("roof effect"); see e.g. Abragam (1962), chapter XI, section B. In this book, positions and intensities are calculated from transition frequencies and probabilities for the transitions between the levels.

For the case $\Delta\nu = 0$, the Hamiltonian commutes with the initial state with the consequence that the density operator remains constant; the Fourier transform of this is just one resonance positioned at zero frequency.

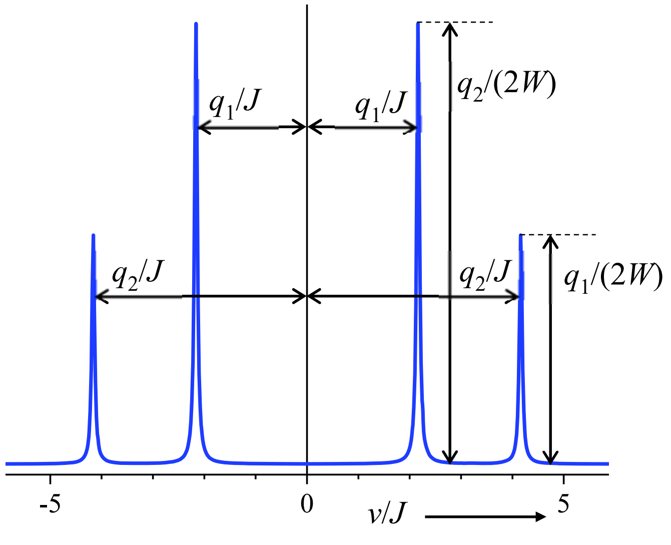

Fig. S1: Line quartet for two J-coupled spins of the type AB with $\Delta\nu = 3J$. Line intensities and positions are drawn in by the variables occurring in the propagation formula (S44).

S3.4.1.2 Example 4D-2: Nutation experiment under influence of homonuclear dipolar interaction

We consider an ensemble of spin pairs $I_1 = ½$ and $I_2 = ½$ with equal resonance positions and homonuclear dipolar interaction which are irradiated at their Larmor frequency and start parallel to **B**0. Then we have the initial assignments $\hat{A} = \hat{I}_{1z} + \hat{I}_{2z}$, $\hat{H} = \hat{H}_{Ix} + \hat{H}_{II}$.

Commutator equations corresponding to Eq. (S41):

$$\begin{aligned}
\left[\hat{H},\left(\hat{I}_{1z}+\hat{I}_{2z}\right)\right] &= i\omega_1\cdot\left(\hat{I}_{1y}+\hat{I}_{2y}\right)\\
\left[\hat{H},\left(\hat{I}_{1y}+\hat{I}_{2y}\right)\right] &= -i\omega_1\left(\hat{I}_{1z}+\hat{I}_{2z}\right) + \frac{3}{2}iD_{\text{ii}}\left(2\hat{I}_{1x}\hat{I}_{2z}+2\hat{I}_{1z}\hat{I}_{2x}\right)\\
\left[\hat{H},2\left(\hat{I}_{1x}\hat{I}_{2z}+\hat{I}_{1z}\hat{I}_{2x}\right)\right] &= -\frac{3}{2}iD_{\text{ii}}\left(\hat{I}_{2y}+\hat{I}_{1y}\right) + i\omega_1\left(2\hat{I}_{1y}\hat{I}_{2x}+2\hat{I}_{1x}\hat{I}_{2y}\right)\\
\left[\hat{H},2\left(\hat{I}_{1y}\hat{I}_{2x}+\hat{I}_{1x}\hat{I}_{2y}\right)\right] &= -i\omega_1\left(2\hat{I}_{1z}\hat{I}_{2x}+2\hat{I}_{1x}\hat{I}_{2z}\right)
\end{aligned} \tag{S45}$$

System of spins ½: $\hat{I}_{1z}+\hat{I}_{2z}$ and $\hat{I}_{1y}+\hat{I}_{2y}$ have the norm $\sqrt{2}$, $\hat{I}_{1x}\hat{I}_{2z}-\hat{I}_{1z}\hat{I}_{2x}$ and $\hat{I}_{1y}\hat{I}_{2x}+\hat{I}_{1x}\hat{I}_{2y}$ have the norm $1/\sqrt{2}$. To ensure that the norms of all state operators are equal, we assign $\hat{B}=\hat{I}_{1y}+\hat{I}_{2y}$, $\hat{C}=2\hat{I}_{1z}\hat{I}_{2x}-2\hat{I}_{1x}\hat{I}_{2z}$, $\hat{D}=2\hat{I}_{1y}\hat{I}_{2z}+2\hat{I}_{1z}\hat{I}_{2y}$, $a=\omega_1$ and $b=\frac{3}{2}D_{II}$. Inserting that into Eq. (S42), version 2, gives

$$\begin{aligned}
\hat{I}_{1z}+\hat{I}_{2z} \xrightarrow{\left(\hat{H}_{II}+\hat{H}_{Ix}\right)t} &\left(\hat{I}_{1z}+\hat{I}_{2z}\right)\cdot\left(\cos Wt\cos\frac{3D_{\text{II}}}{4}t+\frac{3D_{\text{II}}}{4W}\sin Wt\sin\frac{3D_{\text{II}}}{4}t\right)\\
&+\left(\hat{I}_{1y}+\hat{I}_{2y}\right)\cdot\frac{\omega_1}{W}\sin Wt\cos\frac{3D_{\text{II}}t}{4}\\
&-2\left(\hat{I}_{1x}\hat{I}_{2z}+\hat{I}_{1z}\hat{I}_{2x}\right)\cdot\frac{\omega_1}{W}\sin Wt\sin\frac{3D_{\text{II}}t}{4}\\
&-2\left(\hat{I}_{1y}\hat{I}_{2x}+\hat{I}_{1x}\hat{I}_{2y}\right)\cdot\left(\cos Wt\sin\frac{3D_{\text{II}}t}{4}-\frac{3D_{\text{II}}}{4W}\sin\frac{Wt}{2}\cos\frac{3D_{\text{II}}t}{4}\right)
\end{aligned} \tag{S46}$$

where $W=\sqrt{\omega_{1I}^2+9D_{II}^2/16}$.

This propagation formula describes the effect of an rf pulse with a limited power on the equilibrium magnetization.

Comments on Equation (S46):

- It will approximate the coupling-free nutation case for $\omega_1 \gg D_{II}$.
- As the coupling frequency increases, so does the nutation frequency W. However, this oscillation is modulated by half of the dipolar frequency.
- As a consequence, the π and $\pi/2$ conditions for achieving maximum and zero y magnetization, respectively, are modified with respect to the coupling-free case. Similar to Equation (S26), this results in

$$\sqrt{\omega_{1I}^2 + 9D_{II}^2/16} \cdot \tau = \begin{cases} \pi/2 & \text{for maximum } M_y \\ \pi & \text{for zero-crossimg of } M_y \end{cases} \tag{S47}$$

- The propagation formula contains expressions for temporary both antiphase and double-quantum coherences.

S3.4.1.3 Example 4D-3: Nutation under quadrupolar interaction, spin 1

We consider an ensemble of isolated $I = 1$ spins (for example ^{2}H or ^{14}N) which are exposed to the interaction of their quadrupole moment with the electric field gradient of the electron cloud together with an rf irradiation.

Initial state and Hamiltonian: $\hat{A} = \hat{I}_{1z} + \hat{I}_{2z}$, $\hat{H} = \hat{H}_{Ix} + \hat{H}_Q$.

Commutator equations corresponding to Eq. (S41):

$$\begin{aligned}
\left[\hat{H}_{Ix} + \hat{H}_Q, \hat{I}_z\right] &= i\omega_1 \hat{I}_y \\
\left[\hat{H}_{Ix} + \hat{H}_Q, \hat{I}_y\right] &= -i\omega_1 \hat{I}_z \quad -i\omega_Q\left(\hat{I}_x\hat{I}_z + \hat{I}_z\hat{I}_x\right) \\
\left[\hat{H}_{Ix} + \hat{H}_Q, \hat{I}_x\hat{I}_z + \hat{I}_z\hat{I}_x\right] &= +i\omega_Q \cdot \hat{I}_y \quad +i\omega_1\left(\hat{I}_x\hat{I}_y + \hat{I}_y\hat{I}_x\right) \\
\left[\hat{H}_{Ix} + \hat{H}_Q, \hat{I}_x\hat{I}_y + \hat{I}_y\hat{I}_x\right] &= -i\omega_1\left(\hat{I}_x\hat{I}_z + \hat{I}_z\hat{I}_x\right)
\end{aligned} \tag{S48}$$

For single spin 1, $\hat{I}_z$, $\hat{I}_y$, $\hat{I}_x\hat{I}_z + \hat{I}_z\hat{I}_x$ and $\hat{I}_x\hat{I}_y + \hat{I}_y\hat{I}_x$ have norm $\sqrt{2}$. Therefore, we assign $\hat{B} = \hat{I}_y$, $\hat{C} = \hat{I}_x\hat{I}_z + \hat{I}_z\hat{I}_x$, $\hat{D} = \hat{I}_x\hat{I}_y + \hat{I}_y\hat{I}_x$, $a = \omega_1$ and $b = \omega_Q$. Inserting that into Eq. (S42), version 2, gives

$$\begin{aligned}
\hat{I}_z \xrightarrow{\left(\hat{H}_Q + \hat{H}_{1x}\right)t} \; & \hat{I}_z \cdot \left(\cos\frac{Wt}{2}\cos\frac{\omega_Q t}{2} + \frac{\omega_Q}{W}\sin\frac{Wt}{2}\sin\frac{\omega_1 t}{2}\right) + \hat{I}_y \cdot \frac{2\omega_1}{W}\sin\frac{Wt}{2}\cos\frac{\omega_Q t}{2} \\
& - \hat{J}_y \cdot \frac{2\omega_1}{W}\sin\frac{Wt}{2}\sin\frac{\omega_Q t}{2} + \hat{J}_z \cdot \left(\cos\frac{Wt}{2}\sin\frac{\omega_Q t}{2} - \frac{\omega_1}{W}\sin\frac{Wt}{2}\cos\frac{\omega_Q t}{2}\right)
\end{aligned} \tag{S49}$$

with $W = \sqrt{4\omega_1^2 + \omega_Q^2}$. This is consistent with the findings of Bloom, Davis and Valic (1980) and Vega and Luz (1987). Again, the result is that nutation takes place faster than ω_{1I} if an interaction is present.

S3.5 Example of a five-dimensional case

Here one example is shown. Again we are dealing with cross polarization, considering finite rf power and assuming that the Hartmann-Hahn condition is satisfied: $\omega_{1I} = \omega_{1S} =: \omega_1$. The Hamiltonian and the frame are the same as for Eqs. (S38) and (S40). The difference lies in the initial state: Here we assume that one spin is polarized and the other is not. The corresponding operator is now $\hat{S}_x$, and the Hamiltonian is as in the example 3D-8:

$\hat{H} = \hat{H}_{Ix} + \hat{H}_{Sx} + \hat{H}_{IS}$.

Commutator equations:

$$
\begin{array}{llllll|ll}
\left[\hat{H},\hat{A}\right]= & & -iD_{\mathrm{IS}}\,\hat{B} & & & & \hat{A}:=\hat{S}_x & \\
\left[\hat{H},\hat{B}\right]= & iD_{\mathrm{IS}}\,\hat{A} & & -i\sqrt{2}\,\omega_1\,\hat{C} & & & \hat{B}:=2\hat{I}_z\hat{S}_y & \\
\left[\hat{H},\hat{C}\right]= & & i\sqrt{2}\,\omega_1\,\hat{B} & & i\sqrt{2}\,\omega_1\,\hat{D} & & \hat{C}:=\sqrt{2}\left(\hat{I}_z\hat{S}_z-\hat{I}_y\hat{S}_y\right) & \text{(S50)} \\
\left[\hat{H},\hat{D}\right]= & & & -i\sqrt{2}\,\omega_1\hat{C} & & iD_{\mathrm{IS}}\,\hat{E} & \hat{D}:=2\hat{I}_y\hat{S}_z & \\
\left[\hat{H},\hat{E}\right]= & & & & -iD_{\mathrm{IS}}\,\hat{D} & & \hat{E}:=\hat{I}_x &
\end{array}
$$

The coefficients are chosen so that all operators have norm 1.

Step 2: The Liouvillian matrix is

$$
\mathbf{L}=i\begin{pmatrix} 0 & D_{IS} & 0 & 0 & 0 \\ -D_{IS} & 0 & \sqrt{2}\,\omega_1 & 0 & 0 \\ 0 & -\sqrt{2}\,\omega_1 & 0 & -\sqrt{2}\,\omega_1 & 0 \\ 0 & 0 & \sqrt{2}\,\omega_1 & 0 & -D_{IS} \\ 0 & 0 & 0 & D_{IS} & 0 \end{pmatrix} \tag{S51}
$$

Step 3: We obtain the superpropagator matrix $\mathbf{U}=\exp(-i\mathbf{L}t)$ with the elements

$$
U_{11}=U_{55}=\frac{1}{2}\left[\left(\cos D_{\mathrm{IS}}t+\frac{4\omega_1^2+D_{\mathrm{IS}}^2\cos qt}{q^2}\right)\right.\quad;\quad U_{15}=U_{51}=\frac{1}{2}\left[\left(-\cos D_{\mathrm{IS}}t+\frac{4\omega_1^2+D_{\mathrm{IS}}^2\cos qt}{q^2}\right)\right.
$$

$$
U_{12}=-U_{21}=-U_{45}=U_{54}=\frac{1}{2}\left(\frac{D_{\mathrm{IS}}}{q}\sin qt+\sin D_{\mathrm{IS}}t\right)
$$

$$
U_{13}=U_{31}=U_{35}=U_{53}=\frac{D_{\mathrm{IS}}\omega_1}{q^2}(1-\cos qt)\quad;\quad U_{33}=\frac{D_{IS}^2+4\omega_1^2\cos qt]}{q^2}
$$

$$
U_{14}=-U_{41}=-U_{25}=U_{52}=\frac{1}{2}\left(\frac{D_{\mathrm{IS}}\sin qt}{q}-\sin D_{\mathrm{IS}}t\right)\quad;\quad U_{23}=-U_{32}=-U_{34}=U_{43}=\frac{2\omega_1\sin qt]}{q}
$$

$$
U_{22}=U_{44}=\frac{1}{2}(\cos D_{\mathrm{IS}}t+\cos qt]))\quad;\quad U_{24}=U_{42}=\frac{1}{2}(-\cos D_{\mathrm{IS}}t+\cos qt])
$$

where $q^2:=D_{IS}^2+4\omega_1^2$. From the propagator matrix we get the following propagation formula for the initially polarized spin:

$$
\boxed{\begin{aligned}
\hat{S}_x \xrightarrow{\left(\hat{H}_{IS}+\hat{H}_{Ix}+\hat{H}_{Sx}\right)\cdot t}\ & \frac{1}{2}\left[\left(\cos D_{\mathrm{IS}}t+\frac{4\omega_1^2+D_{\mathrm{IS}}^2\cos qt}{q^2}\right)\hat{S}_x\right. \\
& -\left(\frac{D_{\mathrm{IS}}}{q}\sin qt+\sin D_{\mathrm{IS}}t\right)2\hat{I}_z\hat{S}_y \;+\; 4\frac{D_{\mathrm{IS}}\omega_1}{q^2}(1-\cos qt)\left(\hat{I}_z\hat{S}_z-\hat{I}_y\hat{S}_y\right) \\
& \left.+\left(\frac{D_{\mathrm{IS}}}{q}\sin qt-\sin D_{\mathrm{IS}}t\right)2\hat{I}_y\hat{S}_z \;+\; \left(-\cos D_{\mathrm{IS}}t+\frac{4\omega_1^2+D_{\mathrm{IS}}^2\cos qt}{q^2}\right)\hat{I}_x\right]
\end{aligned}} \tag{S52}
$$

For the cross-polarization experiment, the behaviour of the initially unpolarized spin is also of interest:

$$\begin{aligned} \hat{I}_x \xrightarrow{\left(\hat{H}_{IS}+\hat{H}_{Ix}+\hat{H}_{Sx}\right)\cdot t} & \frac{1}{2}\Bigg[\left(-\cos D_{\mathrm{IS}}t+\frac{4\omega_1^2+D_{\mathrm{IS}}^2\cos qt}{q^2}\right)\hat{S}_x \\ & -\left(\frac{D_{\mathrm{IS}}}{q}\sin qt-\sin D_{\mathrm{IS}}t\right)2\hat{I}_z\hat{S}_y + 4\frac{D_{\mathrm{IS}}\omega_1}{q^2}(1-\cos qt)\left(\hat{I}_z\hat{S}_z-\hat{I}_y\hat{S}_y\right) \\ & -\left(\frac{D_{\mathrm{IS}}}{q}\sin qt+\sin D_{\mathrm{IS}}t\right)2\hat{I}_y\hat{S}_z + \left(\cos D_{\mathrm{IS}}t+\frac{4\omega_1^2+D_{\mathrm{IS}}^2\cos qt}{q^2}\right)\hat{I}_x\Bigg] \end{aligned} \quad \text{(S53)}$$

The sum and difference of formulae (S52) and (S53) give Equations (S38) and (S40), respectively, when the latter are used under Hartmann-Hahn condition.

S3.6 Examples of a six-dimensional case

S3.6.1.1 Example 6D-1: CP for IS, finite rf power

This example contains the same situation as in the 5D case except that the Hartmann-Hahn condition does not need to be satisfied:

$$\begin{array}{llllllll|l} \left[\hat{H},\hat{A}\right]= & & -iD_{\mathrm{IS}}\hat{B} & & & & & & \hat{A}:=\hat{S}_x \\ \left[\hat{H},\hat{B}\right]= & iD_{\mathrm{IS}}\hat{A} & & -i\omega_{1S}\hat{C} & +i\omega_{1I}\hat{D} & & & & \hat{B}:=2\hat{I}_z\hat{S}_y \\ \left[\hat{H},\hat{C}\right]= & & i\omega_{1S}\hat{B} & & & +i\omega_{1I}\hat{E} & & & \hat{C}:=2\hat{I}_z\hat{S}_z \\ \left[\hat{H},\hat{D}\right]= & & -i\omega_{1I}\hat{B} & & & -i\omega_{1S}\hat{E} & & & \hat{D}:=2\hat{I}_y\hat{S}_y \\ \left[\hat{H},\hat{E}\right]= & & & -i\omega_{1I}\hat{C} & +i\omega_{1S}\hat{D} & & iD_{\mathrm{IS}}\hat{F} & & \hat{E}:=2\hat{I}_y\hat{S}_z \\ \left[\hat{H},\hat{F}\right]= & & & & & -iD_{\mathrm{IS}}\hat{E} & & & \hat{F}:=\hat{I}_x \end{array} \quad \text{(S54)}$$

Here the second commutator deviates from the principles mentioned above: The term from which $\hat{A}$ was separated was not adopted as a new basis operator in its entirety, but continued to be used in two parts. The Liouvillian matrix is

$$\mathbf{L}=i\begin{pmatrix} 0 & D_{\mathrm{IS}} & 0 & 0 & 0 & 0 \\ -D_{\mathrm{IS}} & 0 & \omega_{1S} & -\omega_{1I} & 0 & 0 \\ 0 & -\omega_{1S} & 0 & 0 & -\omega_{1I} & 0 \\ 0 & \omega_{1I} & 0 & 0 & \omega_{1S} & 0 \\ 0 & 0 & \omega_{1I} & -\omega_{1S} & 0 & -D_{\mathrm{IS}} \\ 0 & 0 & 0 & 0 & D_{\mathrm{IS}} & 0 \end{pmatrix} \quad \text{(S55)}$$

With the definition of the 3D section we obtain from the propagator matrix:

$$
\begin{aligned}
\hat{S}_x \quad & \xrightarrow{\left(\hat{H}_{IS}+\hat{H}_{Ix}+\hat{H}_{Sx}\right)\cdot t} \quad \hat{S}_x \cdot \frac{1}{2}\left(\frac{\omega_\Delta^2 + D_{\mathrm{IS}}^2 \cos q_\Delta t}{q_\Delta^2} + \frac{4\omega_\emptyset^2 + D_{\mathrm{IS}}^2 \cos q_\emptyset t}{q_\emptyset^2}\right) \\
& - \; \hat{I}_z \hat{S}_y \cdot D_{\mathrm{IS}} \left(\frac{\sin q_\Delta t}{q_\Delta} + \frac{\sin q_\emptyset t}{q_\emptyset}\right) \\
& + \; 2\hat{I}_z \hat{S}_z \cdot D_{\mathrm{IS}} \left[\frac{\omega_\Delta}{2q_\Delta^2}\left(1-\cos q_\Delta t\right) + \frac{\omega_\emptyset}{q_\emptyset^2}\left(1-\cos q_\emptyset t\right)\right] \\
& + \; 2\hat{I}_y \hat{S}_y \cdot D_{\mathrm{IS}} \left[\frac{\omega_\Delta}{2q_\Delta^2}\left(1-\cos q_\Delta t\right) - \frac{\omega_\emptyset}{q_\emptyset^2}\left(1-\cos q_\emptyset t\right)\right] \\
& + \; \hat{I}_y \hat{S}_z \cdot D_{\mathrm{IS}} \left(\frac{\sin q_\Delta t}{q_\Delta} - \frac{\sin q_\emptyset t}{q_\emptyset}\right) \\
& + \; \hat{I}_x \cdot \frac{1}{2}\left(\frac{4\omega_\emptyset^2 + D_{\mathrm{IS}}^2 \cos q_\emptyset t}{q_\emptyset^2} - \frac{\omega_\Delta^2 + D_{\mathrm{IS}}^2 \cos q_\Delta t}{q_\Delta^2}\right)
\end{aligned}
\qquad \text{(S56)}
$$

This relationship results without restrictions from the sum of Equations (S38) and (S40).

S3.6.1.2 Example 6D-2: LGCP within a triple of one spin *I* and two spins *S*

The problem is similar to example 3D-10. The Hamiltonian is the same: $\hat{H} = \hat{H}_{\mathrm{HH2}} = -D_1\left(\hat{I}_x\hat{S}_{1x} + \hat{I}_y\hat{S}_{1y}\right) - D_2\left(\hat{I}_x\hat{S}_{2x} + \hat{I}_y\hat{S}_{2y}\right)$. However in this example we want to follow the evolution of the magnetizations of the three spins individually. Therefore we define the corresponding operators as the first three basis operators: $\hat{A} = \hat{S}_{1z}$, $\hat{B} = \hat{S}_{2z}$ and $\hat{C} = \hat{I}_z$:

$$
\begin{aligned}
\left[\hat{H}_{\mathrm{HH2}}, \hat{S}_{1z}\right] &\equiv \hat{\hat{L}}_{\mathrm{HH2}}\,\hat{S}_{1z} &&= i\,D_1\hat{R}_1 \\
\left[\hat{H}_{\mathrm{HH2}}, \hat{S}_{2z}\right] &\equiv \hat{\hat{L}}_{\mathrm{HH2}}\,\hat{S}_{2z} &&= i\,D_2\hat{R}_2 \\
\left[\hat{H}_{\mathrm{HH2}}, \hat{I}_z\right] &\equiv \hat{\hat{L}}_{\mathrm{HH2}}\,\hat{I}_z &&= -i\,D_1\hat{R}_1 - i\,D_2\hat{R}_2 \\
\left[\hat{H}_{\mathrm{HH2}}, \hat{R}_1\right] &\equiv \hat{\hat{L}}_{\mathrm{HH2}}\,\hat{R}_1 &&= \frac{i}{2}D_1\left(\hat{I}_z - \hat{S}_{1z}\right) + iD_2\,\hat{Q} \\
\left[\hat{H}_{\mathrm{HH2}}, \hat{R}_2\right] &\equiv \hat{\hat{L}}_{\mathrm{HH2}}\,\hat{R}_2 &&= \frac{i}{2}D_2\left(\hat{I}_z - \hat{S}_{2z}\right) + iD_1\,\hat{Q} \\
\left[\hat{H}_{\mathrm{HH2}}, \hat{Q}\right] &\equiv \hat{\hat{L}}_{\mathrm{HH2}}\,\hat{Q} &&= -\frac{i}{4}\left(D_2\hat{R}_1 + D_1\hat{R}_2\right)
\end{aligned}
$$

with $\hat{R}_\alpha := \hat{I}_x\hat{S}_{\alpha y} - \hat{I}_y\hat{S}_{\alpha x}$; $\alpha \in \{1,2\}$; $\hat{Q} := \hat{I}_z\left(\hat{S}_{1x}\hat{S}_{2x} + \hat{S}_{1y}\hat{S}_{2y}\right)$. The six basis operators have the norms $\left\|\hat{S}_{1z}\right\| = \left\|\hat{S}_{2z}\right\| = \left\|\hat{I}_z\right\| = \sqrt{2}$; $\left\|\hat{R}_1\right\| = \left\|\hat{R}_2\right\| = 1$; $\left\|\hat{Q}\right\| = 1/2$. This gives the Liouvillian matrix

$$\mathbf{L} = i\begin{pmatrix} 0 & 0 & 0 & -D_1/\sqrt{2} & 0 & 0 \\ 0 & 0 & 0 & 0 & -D_2/\sqrt{2} & 0 \\ 0 & 0 & 0 & D_1/\sqrt{2} & D_2/\sqrt{2} & 0 \\ D_1/\sqrt{2} & 0 & -D_1/\sqrt{2} & 0 & 0 & -D_2/2 \\ 0 & D_2/\sqrt{2} & -D_2/\sqrt{2} & 0 & 0 & -D_1/2 \\ 0 & 0 & 0 & D_2/2 & D_1/2 & 0 \end{pmatrix}.$$

and via propagator $\mathbf{U} = \exp(-i\mathbf{L}t)$ the propagation formulae

$$\hat{I}_z \to \hat{I}_z \cdot \cos^2\frac{qt}{2} + \hat{S}_{1z} \cdot \frac{D_1^2}{q^2}\sin^2\frac{qt}{2} + \hat{S}_{2z} \cdot \frac{D_2^2}{q^2}\sin^2\frac{qt}{2} - \hat{R}_1 \cdot \frac{D_1}{q}\sin qt$$
$$- \hat{R}_2 \cdot \frac{D_2}{q}\sin qt - 4\hat{Q} \cdot \frac{D_1 D_2}{q^2}\sin^2\frac{qt}{2}$$

$$\hat{S}_{1z} \to \hat{S}_{1z} \cdot \frac{\left(D_2^2 + D_1^2\cos\frac{qt}{2}\right)^2}{q^4} + \hat{S}_{2z} \cdot \frac{4D_1^2 D_2^2 \sin^4\frac{qt}{4}}{q^4} + \hat{I}_z \cdot \frac{D_1^2}{q^2}\sin^2\frac{qt}{2} + \hat{R}_1 \cdot \frac{2D_1\left(D_2^2 + D_1^2\cos\frac{qt}{2}\right)\sin\frac{qt}{2}}{q^3}$$
$$-\hat{R}_2 \cdot \frac{8D_1^2 D_2 \cos\frac{qt}{4}\sin^3\frac{qt}{4}}{q^3} + \hat{Q} \cdot \frac{8D_1 D_2\left(D_2^2 + D_1^2\cos\frac{qt}{2}\right)\sin^2\frac{qt}{4}}{q^4}$$

$$\hat{S}_{2z} \to \hat{S}_{1z} \cdot \frac{4D_1^2 D_2^2 \sin^4\frac{qt}{4}}{q^4} + \hat{S}_{2z} \cdot \frac{\left(D_1^2 + D_2^2\cos\frac{qt}{2}\right)^2}{q^4} + \hat{I}_z \cdot \frac{D_2^2}{q^2}\sin^2\frac{qt}{2} - \hat{R}_1 \cdot \frac{8D_2^2 D_1 \cos\frac{qt}{4}\sin^3\frac{qt}{4}}{q^3}$$
$$+\hat{R}_2 \cdot \frac{2D_2\left(D_1^2 + D_2^2\cos\frac{qt}{2}\right)\sin\frac{qt}{2}}{q^3} + \hat{Q} \cdot \frac{8D_1 D_2\left(D_1^2 + D_2^2\cos\frac{qt}{2}\right)\sin^2\frac{qt}{4}}{q^4}$$

Assuming an initial state where the two S spins are polarized and *I* not, we obtain by adding the last two propagation rules:

$$\boxed{\begin{aligned} \hat{S}_{1z} + \hat{S}_{2z} \to \hat{I}_z \cdot \sin^2\frac{qt}{2} + \hat{S}_{1z} \cdot \frac{D_2^2 + D_1^2\cos^2\frac{qt}{2}}{q^2} + \hat{S}_{2z} \cdot \frac{D_1^2 + D_2^2\cos^2\frac{qt}{2}}{q^2} \\ + \hat{R}_1 \cdot \frac{D_1}{q}\sin qt + \hat{R}_2 \cdot \frac{D_2}{q}\sin qt - \hat{Q} \cdot 4\frac{D_1 D_2}{q^2}\sin^2\frac{qt}{2} \end{aligned}}$$

This propagation rule can be reformulated as

$$\hat{S}_{1z} + \hat{S}_{2z} \to \hat{I}_z \cdot \sin^2\frac{qt}{2} + \left(\hat{S}_{1z} + \hat{S}_{2z}\right) \cdot \frac{1 + \cos^2\frac{qt}{2}}{2} + \left(\hat{S}_{1z} - \hat{S}_{2z}\right) \cdot \frac{D_2^2 - D_1^2}{2q^2}\sin^2\frac{qt}{2}$$
$$+ \hat{R}_1 \cdot \frac{D_1}{q}\sin qt + \hat{R}_2 \cdot \frac{D_2}{q}\sin qt - \hat{Q} \cdot 4\frac{D_1 D_2}{q^2}\sin^2\frac{qt}{2}$$

This rule describes an oscillatory exchange of polarization between the spins and some non-observable states.

S3.7 If possible: Decomposition of the Hamiltonian

If the total Hamiltonian consists of two parts $\hat{H} = \hat{H}_1 + \hat{H}_2$ and if these parts commute with each other and satisfy the condition $\left[\hat{H}_1, \hat{H}_2\right] = 0$, then these parts can be considered as acting separately.

If, in addition, both $\hat{H}_1$ and $\hat{H}_2$ satisfy together with $\hat{A}$ the relations

$$\left[\hat{H}_{1;2}, \hat{A}\right] = i\lambda_{1;2}\, \hat{B}_{1;2} \quad \text{and} \quad \left[\hat{H}_{1;2}, \hat{B}_{1;2}\right] = -i\lambda_{1;2}\, \hat{A}$$

the propagation formula will be

$$\begin{aligned}
\hat{A} &\xrightarrow{\hat{H}_1 t} \hat{A}\cos\lambda_1 t + \hat{B}_1 \sin\lambda_1 t \\
&\xrightarrow{\hat{H}_2 t} \left(\hat{A}\cos\lambda_1 t + \hat{B}_1 \sin\lambda_1 t\right)\cos\lambda_2 t \quad + \quad \left(\hat{B}_2 \cos\lambda_1 + \left[\hat{H}_2, \hat{B}_1\right]\sin\lambda_1 t\right)\sin\lambda_2 t \\
&= \frac{1}{2}\left\{\begin{array}{l} \hat{A}\left[\cos(\lambda_1 + \lambda_2)t + \cos(\lambda_1 - \lambda_2)t\right] \\ +\left(\hat{B}_1 + \hat{B}_2\right)\sin(\lambda_1 + \lambda_2)t + \left(\hat{B}_1 - \hat{B}_2\right)\sin(\lambda_1 - \lambda_2)t + \left[\hat{H}_2, \hat{B}_1\right]\sin\lambda_1 t \sin\lambda_2 t \end{array}\right\}
\end{aligned} \tag{S57}$$

The last expression must be symmetric with respect to an exchange of spins 1 and 2. This is also fulfilled, because $\left[\hat{H}_1, \hat{B}_2\right] = \left[\hat{H}_2, \hat{B}_1\right]$ for $\left[\hat{H}_1, \hat{H}_2\right] = 0$.

S4 References in the SI

Bloom, M., Davis, J. H., and Valic, M. I., Spectral distortion effects Due to Finite Pulse Widths in Deuterium Nuclear Magnetic Resonance Spectroscopy, Canad. J. Phys., 58, 1510-1517, 1980.

Garroway, A. N.: Dipolar Spin Fluctuations in the Rotating Frame: A Relaxation Mechanism, J. Magn. Res., 34, 283-293, 1979.

Krushelnitsky, A., Gauto, D., Camargo, D. C. R., Schanda, P., Saalwächter, K.: Microsecond motions probed by near-rotary-resonance $R_{1\rho}$ ^{15}N MAS NMR experiments: the model case of protein overall-rocking in crystals, J. Biomol. NMR, 71, 53-67, 2018.

Krushelnitsky, A., Hempel, G., Jurack, H., and Ferreira,, T.M.: Rocking motion in solid proteins studies bythe ^{15}N proton-decoupled $R_{1\rho}$ relaxometry, PCCP, 25, pp. 15885-15896, DOI: 10.1039/d3cp00444a, 2023.

Kurz, R., Cobo, M. F., deAzevedo, E. R., Sommer, M., Wicklein, A., Thelakkat, M., Hempel, G., and Saalwächter, K.: ChemPhysChem, vol. 14, no. 13, pp. 3146-3155, DOI: 10.1002/cphc.201300255, 2013.

McArthur, D. A., Hahn, E.L., and Walsted, R.E., Phys. Rev., 188, 609-638, 1969.

Roos, M., Micke, P., and Hempel, G.: Monitoring nuclear spin-flip processes and measuring spin-diffusion constants via hole burning into the magnetization, Chem. Phys. Letters, 536, 147-154, http://dx.doi.org/10.1016/j.cplett.2012.03.074, 2012.

Vega, A. J., and Luz, Z., Quadrupole Echo Distortions as Tool for Dynamic NMR – Application to Molecular Reorientation in Solid Trimethylamine, J. Chem. Phys. 86, 1803-1813, 1987.

Zhang, S., Meier, B. H., and Ernst, R. R.: Polarization Echoes in Nmr, Phys. Rev. Letters, 69, 2149-2151, 1992.
